# The Functioning of the *Drosophila* CPEB Protein Orb Is Regulated by Phosphorylation and Requires Casein Kinase 2 Activity

**DOI:** 10.1371/journal.pone.0024355

**Published:** 2011-09-19

**Authors:** Li Chin Wong, Alexandre Costa, Ian McLeod, Ali Sarkeshik, John Yates, Saw Kyin, David Perlman, Paul Schedl

**Affiliations:** 1 Department of Molecular Biology, Princeton University, Princeton, New Jersey, United States of America; 2 Department of Chemical Physiology, The Scripps Research Institute, La Jolla, California, United States of America; Indiana University, United States of America

## Abstract

The Orb CPEB protein regulates translation of localized mRNAs in *Drosophila* ovaries. While there are multiple hypo- and hyperphosphorylated Orb isoforms in wild type ovaries, most are missing in *orb^F303^*, which has an amino acid substitution in a buried region of the second RRM domain. Using a proteomics approach we identified a candidate Orb kinase, Casein Kinase 2 (CK2). In addition to being associated with Orb *in vivo*, we show that *ck2* is required for *orb* functioning in *gurken* signaling and in the autoregulation of *orb* mRNA localization and translation. Supporting a role for *ck2* in Orb phosphorylation, we find that the phosphorylation pattern is altered when *ck2* activity is partially compromised. Finally, we show that the Orb hypophosphorylated isoforms are in slowly sedimenting complexes that contain the translational repressor Bruno, while the hyperphosphorylated isoforms assemble into large complexes that co-sediment with polysomes and contain the Wisp poly(A) polymerase.

## Introduction

Translational regulation of localized mRNAs plays an essential role in morphological patterning and synaptic plasticity [1]. One mechanism for controlling translation of localized mRNAs is cytoplasmic polyadenylation. Polyadenylation depends upon several key elements in the 3′ UTR of the mRNA. One is a U-rich cytoplasmic polyadenylation element (CPE) that is a target for cytoplasmic polyadenylation element binding proteins (CPEBs) [2], [3]. CPEBs are RRM-type RNA-binding proteins which function in many different contexts including oogenesis in *Xenopus* and *Drosophila* [3], synaptic plasticity in the rat hippocampus [4] , and long-term memory in *Aplysia* [5]. In addition to the CPEBs, other factors which activate or block translation of CPE-containing mRNAs have been identified. Factors promoting translation include poly(A) binding protein (PABP), poly(A) polymerase Gld2, and the cap protein eIF4E, while factors like poly(A) ribonuclease (PARN) and Maskin repress translation [6], [7].

While the mechanisms involved in determining whether (and when) poly(A) tails are added to CPE-containing mRNAs appear to be stage- and mRNA-specific, it is thought that a key regulatory step is CPEB phosphorylation. One model suggests that poly(A) tails remain short when CPEB is unphosphorylated because the ribonuclease activity of PARN exceeds the polymerase activity of Gld2 [8]. Upon CPEB phosphorylation, PARN ribonuclease is expelled from the RNP complex resulting in the lengthening of the poly(A) tail by the Gld2 polymerase and the activation of translation. In another model, Maskin inhibits translation by interacting simultaneously with unphosphorylated CPEB and eIF4E [9]. Phosphorylation of CPEB leads to polyadenylation, while phosphorylation of Maskin releases it from eIF4E. Key phosphorylation sites in the N-terminal domains of the *Xenopus* (Ser174) and mouse (Thr171) CPEB proteins have been identified [10], [11]. It was initially thought that Aurora is responsible for CPEB phosphorylation [12]; however, recent studies have implicated other kinases (MAPK and CamKII) and raised the possibility that different kinases may be deployed in regulating different mRNAs [13], [14].

The *Drosophila* CPEB Orb plays a critical role in oogenesis and functions at multiple steps during egg development [15], [16]. In the null allele, *orb^F343^*, oogenesis arrests before the formation of the 16-cell cyst [16]. Although the 16-cell cyst is formed in the strong loss-of-function allele, *orb^F303^*, oocyte specfication is disrupted and development arrests shortly thereafter. The presumptive oocyte initially accumulates *oskar* (*osk*) and *orb* mRNAs and BicD protein; however, these markers for oocyte fate disappear once the chamber pinches off from the germarium [16], [17]. Other markers for oocyte fate such as *BicD* and *K(10)* mRNAs, and Orb protein, never properly localize to the presumptive oocyte in the mutant. In the hypomorphic allele *orb^mel^*, early steps in oogenesis appear normal; however, specification of the dorsal-ventral (DV) axis is disrupted, as are subsequent steps in AP polarity. Orb is thought to function in AP axis-specification in mid-to-late oogenesis by binding to *osk* mRNA which is localized to the posterior pole of the oocyte and promoting translation by polyadenylation [15], [18], [19]. In DV polarity, Orb is required for the localized translation of *grk* mRNA at the dorsal-anterior corner of the oocyte. In *orb^mel^* ovaries, *grk* mRNA is mislocalized and little or no Grk protein is produced [15], [19]. In addition to regulating translation of mRNAs needed for egg chamber development and axis formation, *orb* is also required to localize and activate the translation of *orb* mRNAs in the oocyte [20]. This positive autoregulatory activity ensures that high levels of Orb accumulate specifically in the oocyte, where *orb's* function is required. These autoregulatory activities are mediated through Orb recognition sequences in the *orb* 3′ UTR. Consistent with this idea, oocyte localization and translation of a hybrid *LacZ*-*orb* 3′ UTR mRNA depends upon *orb* activity [20].

*orb* mRNA in the female germline is predicted to encode either a 897 or 915 amino acid protein. Consistent with a possible use of both start sites, there are two closely spaced Orb isoforms in Western blots of standard SDS-PAGE gels [16]. However, it was also possible that the two isoforms are generated by differential modification. In either case, there was evidence that the more slowly migrating isoform is important for *orb* function, as it is absent in the *orb^F303^* mutant. In the studies reported here, we have investigated the origin and function of the two isoforms.

## Results

### The *orb^F303^* mutation alters a Tyr residue in the second RRM domain of Orb

**Figure 1:**
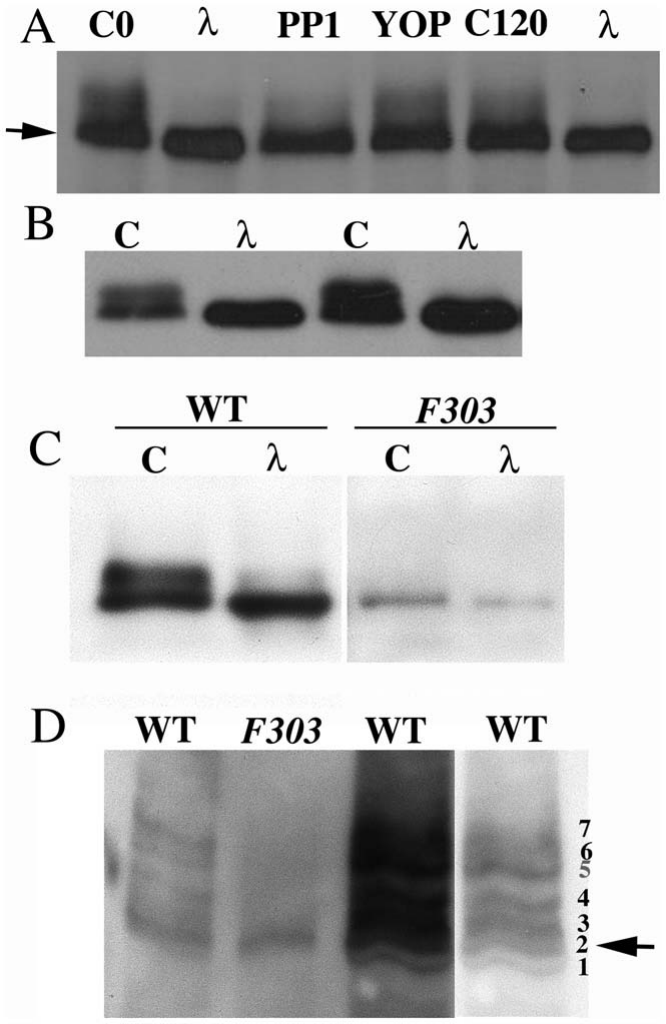
Orb is phosphorylated. (A): Ovary extracts treated with phosphatases as indicated. C0: untreated. λ: λ phosphatase; note difference in migration (arrowhead). PP1: Phosphatase 1; slow isoforms decrease but do not disappear. YOP: Tyrosine Phosphatase. C120: incubated for 2 hrs. (B): C: Untreated extract at two different concentrations. λ: extract treated with λ phosphatase. Note absence of slow band, and shift in the mobility of fast band. (C): Wild type (WT) and *orb^F303^* (*F303*) extracts treated with λ phosphatase (λ). Note increased mobility of *F303* after treatment. (D): Wild type (WT) and *orb^F303^* (*F303*) extracts analyzed using Phos-Tag gel. The two WT phosphoisoforms in SDS-PAGE gels resolve into multiple bands. The *F303* pattern is much simpler. Different WT lanes show different exposures.

In Western blots of ovary extracts, the ∼100 kD Orb protein can be resolved into slow and fast migrating isoforms (Fig. 1A, lane C0). The slower isoform is not observed in *orb^F303^* ovaries ([16]; Fig. 1C) and thus may be important for *orb* function. We considered two explanations for the presence of two isoforms. The first was the use of two closely spaced AUG codons (18 codons apart) for translation initiation that would generate proteins of 897 or 915 amino acids, while the second was some sort of post-translational modification.

To distinguish between these possibilities, we sequenced cDNAs from *orb^F303^* and, as controls, from wild type and *orb^F343^* (which was recovered in the same EMS screen). Ruling out the first possibility, we did not detect a mutation in *orb^F303^* that could potentially eliminate translation products initiated from the upstream AUG and thus give only the smaller isoform. Except for several nucleotide and amino acid polymorphisms that were present in *orb^F303^* and *orb^F343^* but not the wild type, the only difference detected in *orb^F303^* was a T→A mutation that converts residue 742 from a tyrosine (Tyr) to an asparagine (Asn) (Fig. S1). Tyr742 residue is in a conserved region of the second RRM domain. In RRM domains with known structures, the corresponding residue is at a site in a helix that is buried within the protein [21], [22]. Since this Tyr is not expected to interact with RNA or be accessible to solvent, the mutation probably perturbs *orb* RNA binding activity by disrupting the structure of the RRM domain.

### Orb isoforms are generated by phosphorylation

An alternative explanation for the missing isoforms in *orb^F303^* is that the proper folding of the second RRM domain is a prerequisite for post-translational modifications. In *Xenopus*, CPEB activation is triggered by phosphorylation [12]. If there is an equivalent connection between Orb phosphorylation and activation, this modification could potentially account for the isoform profile as phosphorylation is known to retard electrophoretic mobility.

To test this idea, we treated ovary extracts with three phosphatases. As the *orb^F303^* lesion is a tyrosine residue, we first tested YOP tyrosine phosphatase (YOP). YOP has only a slight effect on the Orb isoforms (Fig. 1A). Thus, if the buried Tyr742 residue phosphorylated *in vivo*, it is either not susceptible to YOP dephosphorylation *in vitro*, or else its removal is not sufficient to greatly alter isoform profile. Likewise, if other Orb Tyr residues are phosphorylated, their removal has only a small effect.

We next tested the Ser/Thr Protein Phosphatase 1 (PP1). Fig. 1A shows that the slowly migrating isoforms largely disappear after PP1 treatment, collapsing into more rapidly migrating isoforms. While this finding indicates that slow isoforms are generated by phosphorylation of Ser/Thr residues, not all of the slow isoforms disappear after PP1 treatment even after prolonged digestion (Fig. 1A). One reason why PP1 might not completely convert the slow into the fast isoforms is that Orb is phosphorylated not only on Ser/Thr but also on Tyr. To test this possibility, we used λ phosphatase which dephosphorylates Ser/Thr and Tyr. As seen in Fig. 1A and B, the isoform profile after λ phosphatase treatment differs from the other phosphatase in that all of the slow isoforms collapse into a single band. Moreover, the λ phosphatase band consistently migrates slightly more rapidly than the fast isoforms in untreated extracts (compare the control with λ in Fig. 1A and B). Thus, it would appear that both isoforms in wild type extracts are phosphorylated. If this is the case, then the upper band is expected to be “hyperphosphorylated” because its mobility shows the greatest retardation compared to the λ phosphatase-treated sample, while the lower band would be “hypophosphorylated.” We conclude that Orb is phosphorylated not only on Ser/Thr but also on Tyr residues.

### The Orb^F303^ protein is hypophosphorylated

Since both the slow- and fast-migrating Orb isoforms in wild type appear to be phosphorylated, we re-examined Orb^F303^. As reported previously, we found that the slow isoforms are largely missing in *orb^F303^/Df(3)M95A* ovaries (Fig. 1C). When extracts from *orb^F303^/Df(3)M95A* ovaries are λ phosphatase treated, the resulting band migrates slightly more rapidly than the untreated Orb^F303^ band (compare λ and C in Fig. 1C). Moreover, the small shift in mobility is similar to that seen for the fast wild type isoforms following λ phosphatase treatment. This would suggest that Orb^F303^ is likely to be “hypophosphorylated.”

Since the mobility difference is slight, we analyzed Orb phosphoisoforms using a Phos-Tag SDS polyacrylamide gel (SDS-PAGE) [23]. The degree of retardation in this gel system is mostly dependent upon the number of phosphorylated residues, but can also be influenced by their location. In Phos-Tag gels, the two prominent bands seen in standard SDS-PAGE gels resolve into 7 or more distinct bands that are arranged in two groups, a more rapidly migrating group of four and a more slowly migrating group of three (Fig. 1D: see also Fig. S4). If the relative mobilities of the isoforms that constitute the two SDS-PAGE bands are retained on Phos-Tag gels, then the lower “hypophosphorylated” band consist of at least four isoforms (Fig. 1D: bands 1–4). Similarly, the upper “hyperphosphorylated” band consists of at least three isoforms (bands 5–7). Consistent with the idea that Phos-Tag isoforms correspond to proteins differing in the number and/or location of phosphorylated residues, they collapse into a single band when treated with λ phosphatase (not shown). {As several bands often run as doublets on Phos-Tag gels there may be more than seven phosphoisoforms.}

Fig. 1D shows that most of the more slowly migrating Orb isoforms are also greatly reduced in yield in *orb^F303^*. Instead, there is only one major band that co-migrates with the second of the group of four “hypophosphorylated” isoforms seen in wild type (arrow in Fig. 1D). This finding would be consistent with the λ phosphatase experiments, which suggested that Orb^F303^ has at least one phosphorylated residue.

### Casein Kinase 2 is found in Orb immunoprecipitates

We next sought to identify kinases that might function in Orb phosphorylation. For this purpose, we immunoprecipitated ovary extracts with Orb or, as control, Dorsal antibodies and analyzed the immunoprecipitates using MudPIT mass spectrometry [24], [25]. Altogether ∼170 proteins were detected in Orb but not Dorsal immunoprecipitates (see Table S1). Of these 30 were ribosomal proteins or translation initiation factors. (An additional 20 ribosomal proteins/translation factors were found in both Orb and Dorsal immunoprecipitates.) Other proteins unique to Orb immunoprecipitates included PABP, the *Drosophila* Gld2-homolog Wisp, five predicted RNA helicases, multiple predicted or known RNA binding proteins, components of the siRNA machinery and proteins involved in decapping and RNA turnover, (see also [26]). There were also several proteins (e.g., Bicaudal-C, Encore, Didum, Ovarian tumor and Oskar) implicated in mRNA localization in *Drosophila* ovaries.

Only two protein kinases, SR Protein Kinase 2 (SRPK2) and Casein Kinase 2 (CK2), were found in Orb, but not control immunoprecipitates (Table S1). Both kinases phosphorylate Ser and/or Thr (though CK2 substrates are usually Ser not Thr residues). SRPK2 is thought to modify splicing factors containing Serine/Arginine (SR) domains and is essential for spliceosome assembly [27]. CK2, by contrast, is found in the nucleus and cytoplasm, and phosphorylates proteins that function in many different processes. In the studies reported here we focused on CK2.

CK2 is a heterotetramer consisting of two catalytic α-subunits and two regulatory β-subunits. Both subunits were detected in Orb immunoprecipitates [28], [29]. We further confirmed the Orb-CK2 association by immunoprecipitating ovary extracts with antibodies against the CK2 holoenzyme and probing with Orb (Fig. S2). Consistent with the idea that this association might be relevant to Orb phosphorylation, there are a total twelve predicted CK2 phosphorylation sites in the Orb protein [30]. Two of these are in the C-terminal half of the protein while all of the other sites are in the N-terminal half.

**Figure 2:**
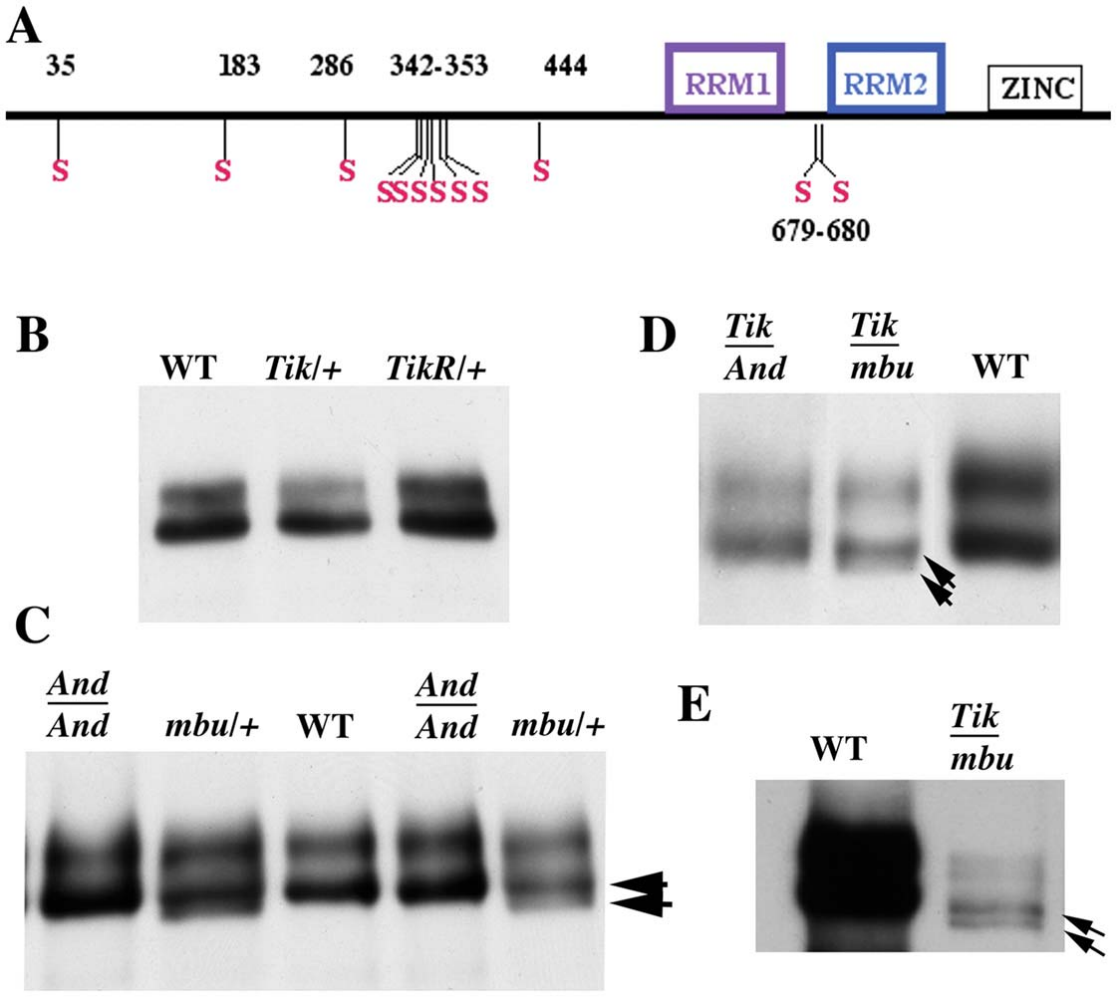
Orb phosphorylation is altered in *ck2* ovaries. (A) Map of the Orb protein showing the RRM and zinc finger domains in the C-terminal half of the protein. Also shown are the positions of the predicted CK2 phosphorylation sites in the poorly conserved N-terminal half of the protein and the pair of predicted CK2 phosphorylation sites in the linker region between the two RRM domains in the C-terminal half of the protein [30]. (B) WT: wild type females; *Tik/+*: *ck2α^Tik^/+* females; *TikR/+*: *ck2α^TikR^/*+ females. Underexposed films and Image J software was used to measure the ratio of fast to slow phosphoisoforms isoforms. Ratio of fast to slow phosphoisoforms in WT is 0.77 (lane 1; range of 0.74–0.84, n = 6). This ratio is reduced in *ck2α^Tik^/+* to 0.43 (lane 2; range 0.40–0.45, n = 3). *ck2α^TikR^/+* (*TikR/+*) ratio is 0.66 (lane 3: range of 0.64–0.68, n = 3). (C) *And/And*: *ck2β^And^/ck2β^And^* females; *mbu/+*: *ck2β^mbuΔA26-2L^/+* females; WT: wild type females; *And/And*: *ck2β^And^/ck2β^And^* females; *mbu/+*: *ck2β^mbuΔA26-2L^/+* females. The ratios of fast to slow in *ck2β^And^/ck2β^And^* is 0.58 (lane 1; n = 3); 0.74 for *ck2β^mbuΔA26-2L^/+* (lane 2; n = 3). Note: A “novel” phosphoisoform that migrates like λ phosphatase treated Orb is seen in *ck2β^mbuΔA26-2L^/+* females (arrowhead). Lanes 4 and 5 are the same as lanes 1 and 2, except that half as much extract was used. (D) Ratio of fast to slow is reduced in *ck2α*/*β trans*-heterozygotes *ck2α^Tik^/ck2β^And^* (*Tik/And*; ratio: 0.37; lane 1; n = 3) and *ck2α^Tik^/ck2β^mbuΔA26-2L^* (*Tik/mbu*; ratio 0.35; lane 2; n = 3). Novel phosphoisoform in *ck2α^Tik^/ck2β^mbuΔA26-2L^* indicated by arrowhead. (E) Novel isoform in *ck2α^Tik^/ckβ^mbuΔA26-2L^* (*Tik/mbu*) females. Position of faster migrating band (lower arrow) coincides with λ phosphatases treated Orb. The upper “hyperphosphorylated” band in *Tik/mbu* also resolves into two distinct species. By contrast, the “hyperphosphorylated” band from (even underloaded) WT females is broad, and never resolves into distinct bands on standard SDS-PAGE gels.

Since CK2 is known to be quite promiscuous in its specificity, and the consensus motif is not especially specific, we asked whether the potential CK2 serine phosphorylation sites are located within conserved regions of the Orb protein. The ∼385 amino acid C-terminal domain contains the two RRM domains and a zinc finger. The sequence of much of this domain is highly conserved across the Drosophilids and in other more distantly related insects. The two potential C-terminal CK2 serine phosphorylation sites (aa 679 and 680) are located next to each other in the 35 amino acid longer linker region separating the two Orb RRM domains (Fig. 2A). Unlike the mutated Tyr742 residue in *orb^F303^*, these two Ser residues are likely to be in an exposed configuration. Moreover, changes in the conformation of the linker, and thus their accessibility to kinase action, could accompany RNA binding [22]. As shown in Fig. S3, the linker sequence of Orb, as well as both potential CK2 phosphorylation sites, are highly conserved in the Drosophilids.

The other predicted CK2 phosphorylation sites are in the ∼530 aa N-terminal domain. Unlike the C-terminal domain, this domain is very poorly conserved overall; however, it does contain short blocks of conserved sequences interspersed with blocks of unrelated or highly divergent sequences. One of the putative CK2 sites (Ser444) falls in a sequence that is poorly conserved even in the closely related *D. willistoni* and thus is unlikely to be critical for function (Fig. S3). Two of the other CK2 sites, Ser35 and Ser183 are located in short partially conserved sequence blocks; however, both of these putative *D. melanogaster* CK2 sites are present only in *D. yakuba* and not other more distantly related flies. In the case of Ser35, both *D. mojavensis* and *D. virilis* have a predicted CK2 site two amino acids over from the site in *D. melanogaster* and *D. yakuba*. In the case of Ser183, the putative CK2 site in *D. mojavensis* and *D. virilis* is shifted to an adjacent Ser residue (Fig. S3).

The seven remaining predicted *D. melanogaster* CK2 sites in the N-terminus are embedded in two highly conserved sequence blocks. One of the conserved blocks has a single putative CK2 site, Ser286. The other block has six very closely spaced potential CK2 phosphorylation sites, Ser342, Ser344, Ser347, Ser350, Ser351 and Ser353 (Fig. 2A). Like their immediately flanking sequences, all seven of these putative CK2 phosphorylation sites are conserved in the five *Drosophila* species we examined (Fig. S3).

In our analysis of Orb IPs using mass spectrometry we typically obtained only about 20–25% coverage of the Orb protein. For this reason, we did not expect to be able to identify the potentially large collection of phosphorylated peptides that would be generated from the various Orb phosphoisoforms evident in Phos-Tag gels. However, we did detect phosphorylated tryptic peptides spanning the 342–353 cluster of six predicted CK2 phosphorylation sites in the N-terminal domain and the 679–680 pair of predicted CK2 phosphorylation sites in the C-terminal domain. In both cases, there were multiple phosphorylated residues; however, we were not able to unambiguously assign the phosphosite localization to the predicted CK2 sites in the peptides with high confidence.

### Orb phosphorylation depends on *ck2* activity

The fact that nine of the predicted *D. melanogaster* CK2 phosphorylation sites are found in other *Drosophila* species and are located in highly conserved regions of the Orb protein would support the idea that the physical association between CK2 and Orb may not be just coincidental. For this reason we examined the Orb phosphoisoform profile in ovaries of females partially compromised for *ck2*. We tested two alleles of *timekeeper (Tik)*, which encodes the catalytic subunit, CK2α. The first, *ck2α^Tik^*, dominantly lengthens the circadian period as a heterozygote and is lethal as a homozygote. The second, *ck2α^TikR^*, is a spontaneous partial revertant of *ck2α^Tik^*. It has a normal circadian period as a heterozygote, but is still homozygous lethal [31]. Genetic and biochemical experiments indicate that *ck2α^Tik^* reduces *ck2* activity in heterozygotes by interfering with wild type CK2α subunits. By contrast, though the kinase activity of CK2α*^TikR^* is also compromised *in vitro*, it doesn't interfere with the wild type subunit *in vivo* and *ck2* activity in heterozygotes is close to wild type [31]. We also tested two alleles of the regulatory subunit, CK2β. One of these, *ck2β^And^* is a weak homozygous viable hypomorph. The other allele, *ck2β^mbuΔA26-2L^*, is homozygous lethal. Consistent with a role in Orb phosphorylation, we find that mutations in both subunits alter the Orb phosphoisoform profile.

In wild type, the slowly migrating “hyperphosphorylated” isoforms are slightly less abundant than the faster “hypophosphorylated” isoforms and the average ratio of slow to fast is ∼0.8 (Fig. 2B: see legend for description and results of the ratio measurements). We find that there is a consistent reduction in the relative amount of the slower isoforms in ovaries from females heterozygous for the dominant negative *ck2α^Tik^* allele and the average ratio is 0.4, or about half that of wild type. By contrast, in females heterozygous for the partial revertant, *ck2α^TikR^*, which are expected to have higher levels of *ck2* activity than *ck2α^Tik^*, the average ratio is ratio is close to wild type (∼0.7). For females homozygous for the weak *ck2β^And^* allele, the ratio of “hyper-“to “hypophosphorylated” isoforms is ∼0.6 (Fig. 2C). Though this reduction is smaller than *ck2α^Tik^/+*, the ratio is only ∼3/4^th^ that of wild type. A different result was obtained for *ck2β^mbuΔA26-2L^/+* females. In these flies the slow/fast ratio is close to wild type (∼0.7); however, the isoform pattern on SDS-PAGE gels is unusual. Though the wild type “hypophosphorylated” isoforms consist of several phosphoproteins, they are not resolved on SDS-PAGE gels. By contrast, the hypophosphorylated isoforms in *ck2β^mbuΔA26-2L^/+* females separate into two closely spaced bands (arrows: Fig. 2C: see also 2E). The upper band co-migrates with “bulk” wild type hypophosphorylated isoforms, while the lower migrates like λ phosphatase-treated Orb.

We also examined the Orb phosphoisoforms in females *trans*-heterozygous for *ck2α^Tik^* and either *ck2β^And^* or *ck2β^mbuΔA26-2L^*. The ratio of the two isoforms in both *ck2α^Tik^*/*ck2β^And^* (∼0.4) and *ck2α^Tik^/ck2β^mbuΔA26-2L^* (∼0.4) was about half that of wild type (∼0.8). In addition, as was observed in *ck2β^mbuΔA26-2L^/+* females, the fast band resolves into two bands in *ck2α^Tik^/ck2β^mbuΔA26-2L^* females (arrows: Fig. 2D & E). Similar, if not more dramatic changes in the phosphoisoform profile are evident when *ck2α^Tik^*/*ck2β^And^* and *ck2α^Tik^/ck2β^mbuΔA26-2L^* extracts are analyzed using Phos-Tag gels (see Fig. S4).

### Reducing *ck2* enhances *orb* DV polarity defects

The effects of partially reducing *ck2* activity on the phosphoisoform profile indicate that this kinase is needed (directly or indirectly) for Orb phosphorylation. An important question is whether *ck2* also impacts *orb* function. To address this question we tested for genetic interactions. *orb* is weakly haploinsufficient in the DV polarity pathway, and 5–10% of the eggs laid by females heterozygous for strong alleles like *orb^F343^* have ventralized chorions due to defects in *grk* mRNA localization and translation. These DV polarity defects can be exacerbated by an *hsp83* transgene (*HD19G*) which constitutively expresses a hybrid mRNA containing *E. coli* β-galactosidase coding sequences fused to the *orb* 3′UTR [20]. Genetic and molecular studies indicate that this transgene behaves as a dominant negative. The *LacZ:orb* 3′UTR mRNA interferes with the *orb* positive autoregulatory loop by competing with the endogenous *orb* mRNA and this downregulates Orb expression. A single copy of *HD19G* in *orb^F343^/+* females increases the percentage of ventralized eggs to 20–30%, while 75% of the eggs are ventralized when there are two copies of the transgene.

**Figure 3:**
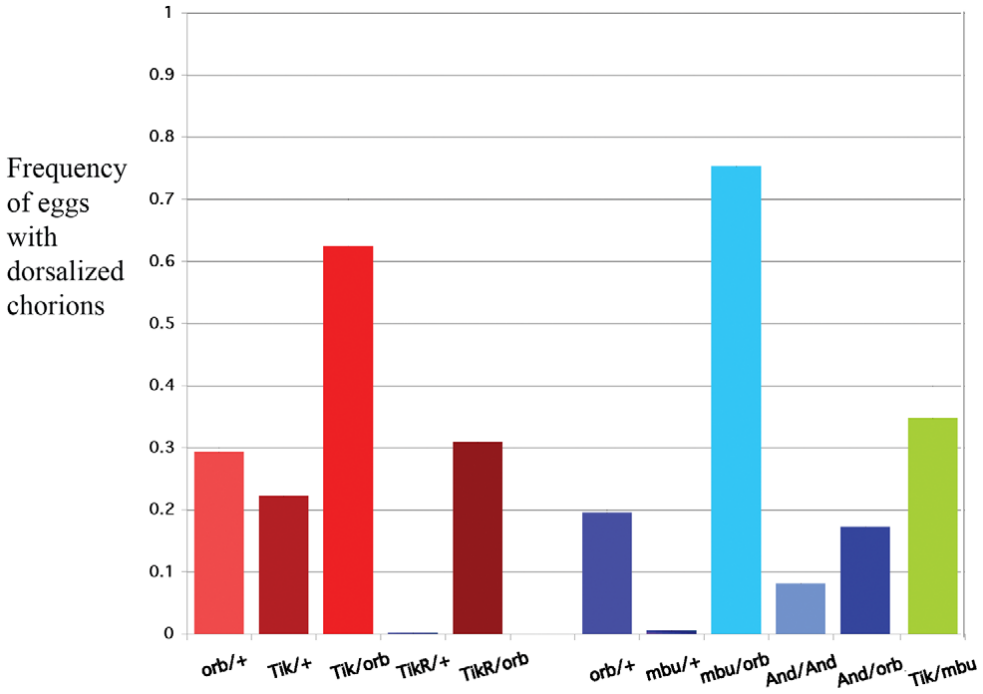
*ck2* is needed for *grk* signaling. Frequency of eggs with ventralized chorions produced by *ck2α*, *ck2β* and various *ck2/orb trans*-heterozygotes. Unless otherwise indicated all *trans* combinations are heterozygous for the mutant allele. Genotypes are: *orb/+*: *HD19G orb^F343^/+* (n = 1808); *Tik/+*: *ck2α^Tik^/+* (n = 1788, p-value = 0.00055); *Tik/orb*: *ck2α^Tik^/HD19G orb^F343^* (n = 1554: p-value = 2.01E-45); *TikR/+*: *ck2α^TikR^/+* (n = 1295: p-value = 3.51E-59); *TikR/*orb: *ck2α^TikR^/HD19G orb^F343^* (n = 2233: p-value = 0.46515); *orb/+*: *HD19G orb^F343^/+* females (n = 1467); *mbu/+*: *ck2β^mbuΔA26-2L^/+* (n = 1505: p-value = 5.83E-61); *mbu/orb*: *ck2β^mbuΔA26-2L^/HD19G orb^F343^* (n = 1892: p-value = 4.91E-85); *And/And*: *ck2β^And^/ck2β^And^* (n = 1450: p-value = 3.62E-29); *And/orb*: *ck2β^And^/HD19G orb^F343^* (n = 1577: p-value = 0.000000001); *Tik/mbu*: *ck2α^Tik^/ck2β^mbuΔA26-2L^* (n = 1034). For wild type females, less than one percent of the eggs have ventralized chorions.

We first asked whether reducing *ck2α* activity by itself has any effect on *grk* signaling. Fig. 3 shows that ∼20% of the eggs laid by *ck2α^Tik^/+* females have ventralized chorions characteristic of a defect in *grk* signaling. Paralleling the effects of the two *ck2α* alleles on the phosphoisoform ratio, the disruption in *grk* signaling is specific for the dominant negative mutation and is not observed when females are heterozygous for the partial revertant *ck2α^TikR^*. We next tested for genetic interactions between *ck2α* and *orb*. As shown in Fig. 3, there is a substantial increase in the frequency of eggs with ventralized chorions when *HD19G orb^F343^* females are *trans*-heterozygous with *ck2α^Tik^* while no interactions are observed for *ck2α^TikR^*.

Further evidence that *ck2* functions in the *grk* signaling pathway and that it's role in this pathway is likely to be connected to *orb* comes from analysis of β-subunit mutations [32]. Consistent with the (modest) change in the Orb phosphoisoform ratio, Fig. 3 shows that nearly 10% of the eggs laid by *ck2β^And^/ck2β^And^* females are ventralized. Not surprisingly, this weak allele shows little interaction with *orb* as a *trans*-heterozygote. On the other hand, there is a potent interaction between *ck2β^mbuΔA26-2L^*, the allele with the unusual phosphoisoform, and *orb*. Whereas there is little evidence of DV polarity defects in *ck2β^mbuΔA26-2L^/+* females, when *ck2β^mbuΔA26-2L^/+* is *trans* to *HD19G orb^F343/^/+* ∼75% of the eggs have ventralized chorions. This strong synergistic interaction would be consistent with a close connection between *ck2* activity and *orb* function in *grk* signaling. Additionally, we find that the *ck2β^mbuΔA26-2L^* allele also dominantly enhances the DV polarity defects evident in *ck2α^Tik^/+* females.

### CK2 is required for *orb* mRNA localization

**Figure 4:**
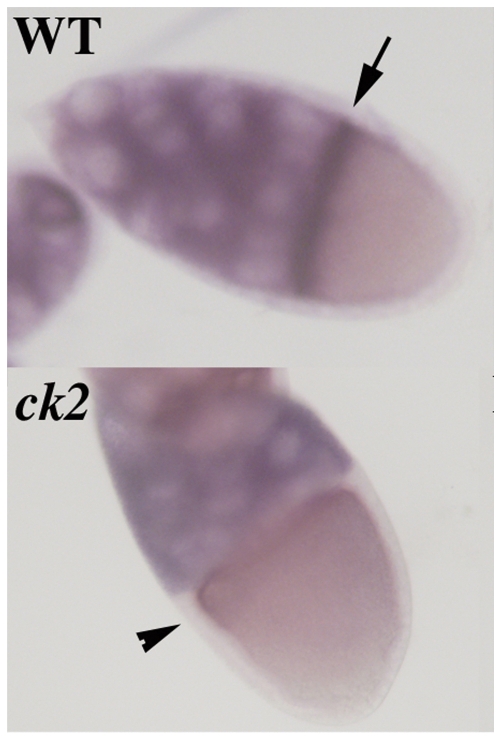
*orb* mRNA localization is disrupted in *ck2*. In WT *orb* mRNA is expressed in nurse cells and localized along anterior margin of oocyte during stages 8–10 (WT: arrow). In *ck2α^Tik^/ck2β^And^*, *orb* mRNA does not accumulate to high levels along anterior margin (ck2: arrowhead). There is a range in the severity of the defects in *orb* mRNA localization along the anterior margin and examples of this range are shown in Fig. S5.

The above experiments demonstrate that *ck2* is required for Orb phosphorylation and argue that the kinase is needed to potentiate *orb* activity in *grk* signaling. We wondered whether *ck2* is required for other *orb* functions. One of the other known *orb* functions is in the localization of *orb* mRNA. Previous studies have shown that *orb* is required for properly localizing *orb* and the chimeric *LacZ-orb* 3′ UTR mRNAs in developing egg chambers [20]. To determine if *ck2* is needed for this function, we examined the localization of *orb* mRNA in ovaries of *ck2α^Tik^/ck2β^And^ trans*-heterozygous females. In wild type, *orb* mRNA localizes to the posterior of the oocyte in pre-vitellogenic stages (1–7) while during stages 8–10 it accumulates preferentially along the anterior margin of the oocyte (arrow in Fig. 4). There were no striking abnormalities in the posterior targeting of *orb* mRNAs in stage 1–7 *ck2α^Tik^/ck2β^And^* chambers; however, we found that *orb* mRNA doesn't concentrate along the anterior margin in stage 8–10 *ck2α^Tik^/ck2β^And^* chambers (Fig. 4 & Fig. S5).

### Orb expression is disrupted when *ck2* activity is reduced

**Figure 5:**
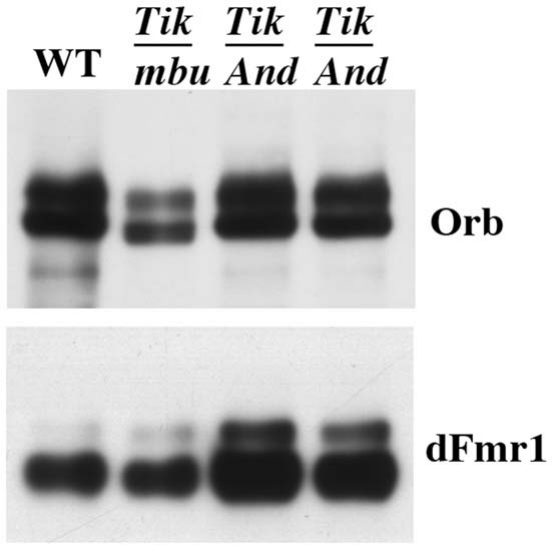
Orb protein levels are reduced in *ck2*. Western blot of wild type and mutant extracts probed with Orb antibody (upper panel) and after striping reprobed with dFMR1 antibody (lower panel). Lanes: WT: wild type; *Tik/mbu*: *ck2α^Tik^/ck2β^mbuΔA26-2L^*; *Tik/And*: two different *ck2α^Tik^/ck2β^And^* extracts. dFMR1 or Snf (Snf data not shown) were used as loading controls. ImageJ software was used to analyze underexposed films. Orb levels in the *trans*-heterozygous combinations shown here are ∼60% of wild type. A similar reduction was observed for *And/mbu*. Orb levels relative to wild type are: *ck2α^Tik^/ck2β^mbuΔA26-2L^*: 0.59 (dFMR1 control: n = 5) and 0.53 (Snf control: n = 3); *ck2α^Tik^/ck2β^And^*: 0.65 (dFMR1 control: n = 6) and 0.67 (Snf control: n = 3); *ck2β^And^/ck2β^mbuΔA26-2L^* 0.51 (dFMR1: control n = 2) and 0.53 (Snf control: n = 2).

Like mRNA localization, Orb expression requires *orb* [20]. If *ck2* is also important for this positive autoregulatory activity, then there should be abnormalities in the accumulation of Orb protein when *ck2* is activity is compromised. To test this prediction, we examined Orb accumulation in ovaries from females *trans*-heterozygous for different *ck2* mutations. In the experiment in Fig. 5, we compared Orb levels in wild type, *ck2α^Tik^/ck2β^mbuΔA26-2^*, and *ck2α^Tik^/ck2β^And^* ovaries using the *Drosophila* fragile X protein, dFMR1, as a loading control. For both *trans*-heterozygous combinations, we estimate that Orb levels are only about 60% that in wild type ovaries. This was also true for *ck2β^And^/ck2β^mbuΔA26-2^* (not shown). Similar reductions in Orb protein were found for all three *trans*-heterozygous combinations when we used the U1/U2 snRNP protein Sans-fille as the loading control instead of dFMR1 (see legend Fig. 5). These findings indicate that Orb protein accumulation depends upon *ck2* activity.

**Figure 6:**
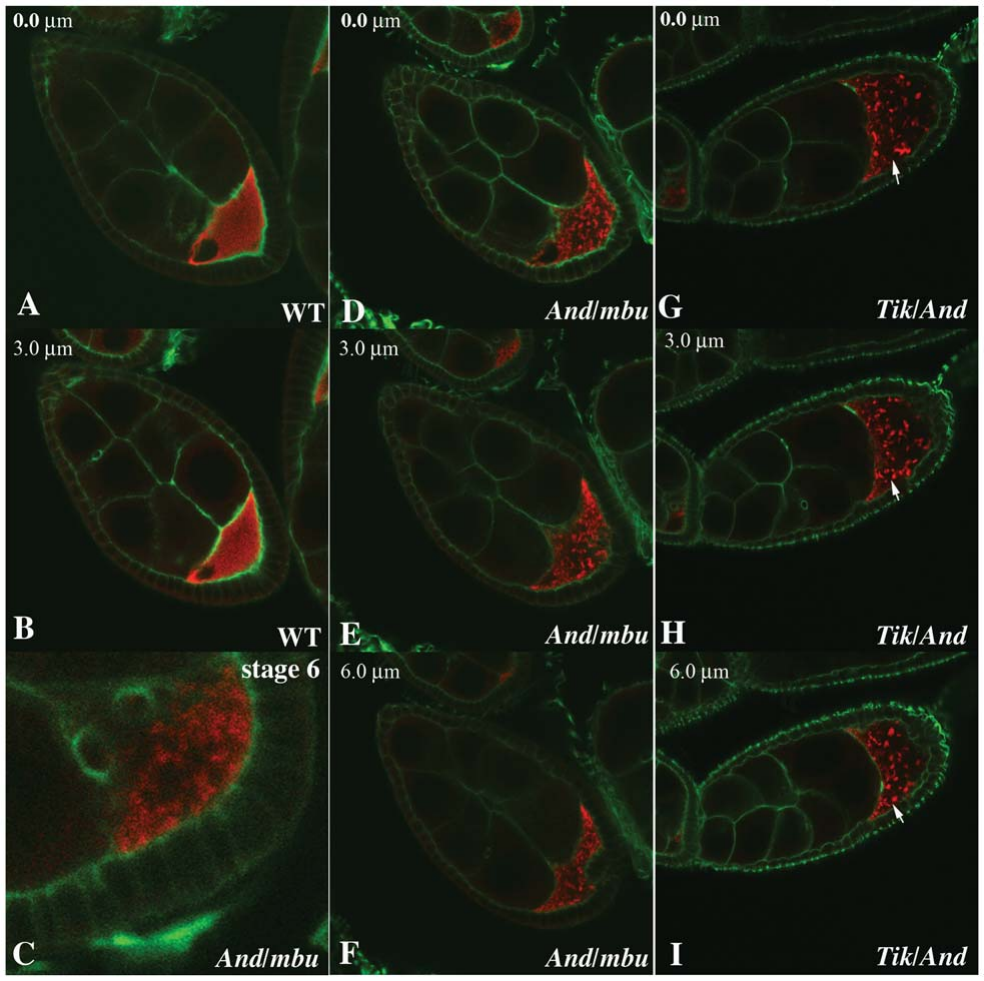
Orb distribution in *ck2* mutants. Distribution of Orb (red) in stage 8 wild type and *ck2* ovaries. Phalloidin (green) shows cytoskeletal organization. Images were successive 1 µm optical sections. Panels A and B: Stage 8 wild type (WT) chamber. Orb distribution (two sections: 0.0 and 3.0 microns) is relatively homogeneous. Panel C: Stage 6 *ck2β^And^/ck2β^mbuΔA26-2L^* chamber. Note discontinuities in Orb distribution. Panels D–F: Optical sections (0.0, 3.0 and 6.0 microns) of stage 8 *ck2β^And^/ck2β^mbuΔA26-2L^* chamber. Note Orb islands and extensive regions of ooplasm lacking Orb. Panels G–I: Sections (0.0, 3.0 and 6.0 microns) of stage 8 *ck2α^Tik^/ck2β^And^* chamber. Orb accumulation is irregular. Arrow shows that an island can be traced through 7 successive sections (in 3.0 and 6.0 microns, but not 0.0 microns).

We also examined the distribution of Orb protein in egg chambers when *ck2* activity is compromised. Fig. 6 shows the Orb distribution in optical sections of wild type and *trans-*heterozygous *ck2β^And^/ck2β^mbuΔA26-2L^* or *ck2α^Tik^/ck2β^And^* chambers raised under the same conditions. In wild type stage 1–9 chambers, Orb is distributed in a relatively homogeneous pattern throughout the oocyte with highest concentrations near the posterior, while there is little protein in the nurse cells. This homogenous distribution can be seen in two optical sections through a wild type stage 8 chamber in Fig. 6A–B. A different pattern is evident in *ck2β^And^/ck2β^mbuΔA26-2L^* (Fig. 6C–F) and *ck2α^Tik^/ck2β^And^* (Fig. 6G–I) *trans*-heterozygous stage 8 chambers. Instead of a relatively homogenous distribution, Orb accumulates in structures that resemble the reticulated sponge bodies described by Snee and MacDonald [33]. Some of these reticulated bodies are large and can be traced through 7 or more 1 µm optical sections (arrows panels G–I). In addition, some regions of the ooplasm lack Orb altogether. For example, there is little if any Orb localized along the posterior cortex of the *ck2α^Tik^/ck2β^And^* chamber shown in Fig. 6G–I, and it is also largely absent from the posterior half of the oocyte. The unusual Orb distribution in stage 8–9 chambers can be traced back to earlier stages where similar, though less pronounced, defects in Orb accumulation are evident in *ck2α^Tik^/ck2β^And^* and *ck2β^And^/ck2β^mbuΔA26-2L^* chambers (Fig. 6C).

### Orb phosphoisoforms are found in distinct protein complexes

**Figure 7:**
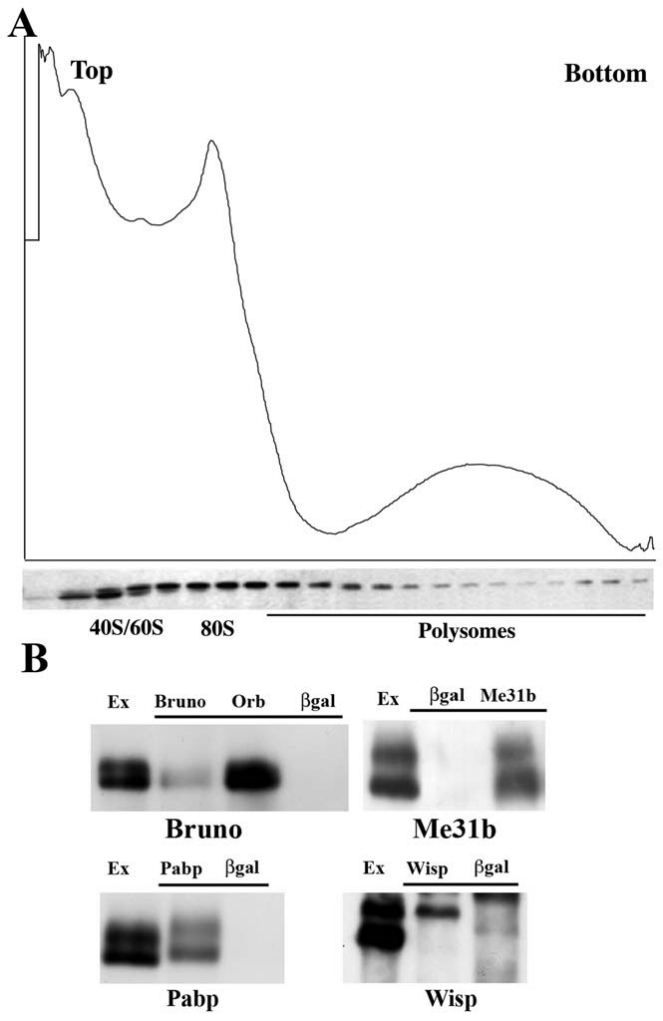
Orb isoforms are in different complexes. (A). Ovary extracts were fractionated on 20–45% sucrose gradients. The fast phosphoisoforms are found in slowly sedimenting complexes in fractions near the top of the gradient. Based on the OD profile, these fractions are expected to contain 40S and 60S ribosomal subunits. The slow phosphoisoform are found in more rapidly sedimenting complexes distributed in fractions from the bottom two-thirds of the gradient. These fractions are expected to contain 80S particles and polysomes as indicated. (B) Ovary extracts (Ex) were immunoprecipitated with Bruno, Me31B, PABP, Wisp or β-galactosidase antibody in the presence of RNase A, and probed with Orb antibodies. First panel: Bruno (Bru), Orb, or β-gal antibodies were used for immunoprecipitation. Second panel: Me31B or β-gal antibodies were used for immunoprecipitation. Third panel: PABP or β-gal antibodies used for immunoprecipitation. Fourth panel: Wisp and β-gal antibodies used for immunoprecipitation. The amount of ovary extract (Ex) loaded is ∼10% of the amount of extract loaded in each immunoprecipitated sample.

The results presented in the previous sections demonstrate that *ck2* is needed for *orb* function in mRNA localization/translation and for generating the normal array of Orb phosphoisoforms. Although several mechanisms might be envisioned, one way in which phosphorylation could promote Orb activity is by altering its interactions with other factors. If this is correct, then the “hypo-” and “hyperphosphoisoforms” should be found in distinct complexes. As a first test of this model, we fractionated ovary extracts on sucrose density gradients. The fast isoforms are found predominantly in slowly sedimenting complexes located near the top of the gradient (Fig. 7A). This region contains mRNPs including the silencing complexes associated with the translational repressor Bruno [34]. In contrast, the slow isoforms are distributed throughout the lower two-thirds of the gradient where they co-sediment with 80S monosomes and translationally active polysomes (Fig. 7A). Consistent with the idea that the slow phosphoisoforms are associated with actively translated polysomal mRNAs, Orb shifts to more slowly sedimenting complexes after polysomes are disrupted by EDTA (not shown). Direct evidence for ribosomal association comes from the collection of proteins identified in Orb immunoprecipitates by mass spectrometry. As indicated in Table S1, Orb is complexed with multiple proteins derived from both ribosomal subunits as well as translation factors like eIF3, eIF4A, eIF4E and PABP.

To provide further evidence that the hypo- and hyperphosphoisoforms are in different complexes, we immunoprecipitated ovary extracts with antibodies against four translation factors, Me31B, Bruno, PABP and Wisp, known to regulate localized mRNA translation (Fig. 7B). These factors also appear to be functionally connected to *orb*. Like *ck2*, they are physically associated with Orb and show dominant genetic interactions with *orb* [18], [35], (unpublished data). The first two proteins, Me31B and Bruno, are translational repressors. Me31B is a DEAD-box helicase [36], while Bruno represses translation by binding to the UTRs of its regulatory targets [37], [38]. PABP and Wisp function as positive regulators of translation. PABP binds to poly(A) tails and promotes translation through factors bound to the mRNA cap [39]. Wisp, the fly Gld2 poly(A) polymerase, activates translation by extending poly(A) tails of translationally silenced mRNAs.

While both Orb phosphoisoforms are found in complexes with the translational repressor Me31B and the activator PABP, a different result is obtained for Bruno and Wisp. Much of the Bruno protein appears to be associated with the hypophosphorylated isoforms. This result is consistent with Bruno's distribution in sucrose gradients where it is found in ∼40S–80S silencing complexes but not in polysomes [34], (unpublished data). It also fits with Snee and Macdonald's [33] studies on the sponge body distribution of Bruno and Orb. Nurse cell sponge bodies have Bruno but not Orb. In the oocyte, sponge bodies near the anterior margin can have both proteins, while only Orb is present in more posterior sponge bodies. In contrast to Bruno, Wisp is preferentially associated with the hyperphosphorylated isoforms. This finding (together with their polysome association) would link hyperphosphorylated Orb isoforms to translational activation.

## Discussion

### Orb activity is modulated by phosphorylation

While only two Orb phosphoisoforms are resolved on SDS-PAGE gels, a combination of phosphatase treatment, analysis of phosphoisoforms in different mutants, and fractionation on Phos-Tag gels indicate that Orb must be phosphorylated at multiple sites on Tyr, Ser and/or Thr residues. At least seven distinct isoforms are resolved on Phos-Tag gels, four faster migrating species and three slower migrating species. Since the degree of retardation in this gel system depends largely upon the number of phosphate residues [23], the set of more rapidly migrating “hypophosphorylated” isoforms are expected to have between zero and three phosphate residue, while the set of more slowly “hyperphosphorylated” isoforms are expected to have four or more phosphate residues. Mobility in the Phos-Tag gel is also influenced by the location of the phosphorylated amino acid, and several bands appear to be doublets. Thus, there may be isoforms that have the same number of phosphorylated residues, but differ in which amino acids are modified. Further studies will be required to determine the number and location of the phosphorylated residues associated with each of the different isoforms.

Several lines of evidence argue that phosphorylation is critical for *orb* activity. One comes from the dramatic effects of the *orb^F303^* mutation: the “hyperphosphorylated” isoforms are absent and the two more slowly migrating “hypophosphorylated” isoforms are largely missing as well. While this would link phosphorylation to Orb function, it is not immediately clear why the Tyr742 mutation has such drastic effects. The simplest model is that Tyr742 must be phosphorylated to generate the other phosphoisoforms. However, Tyr742 is predicted to be on the buried side of the second RRM α-helix and should have essentially no solvent accessibility [21], [22]. Thus, unless it is modified during translation, a scenario in which phosphorylation of this Tyr is obligatory for subsequent phosphorylation elsewhere seems unlikely. A more likely possibility is that the second Orb^F303^ RRM domain does not fold properly and this weakens or eliminates RNA binding. In the absence of RNA-binding, it is possible that oogenesis might arrest at a point prior to when most phosphoisoforms are generated. Arguing against this is the fact that the fast and slow phosphoisoforms are found in mutants in other genes that cause even earlier oogenesis arrest [16], (unpublished data). Another possibility is that phosphorylation of the sites in the Orb protein that generate the collection of more slowly migrating isoforms (isoforms 3–7 in Fig. 1D) requires prior binding to target RNAs. This idea is suggested by the structural changes that are induced when proteins that have two RRM domains interact with RNA. For example, when Sxl binds to its target RNAs, the two RRM domains rearrange so that they clamp around the RNA, while the linker region separating the two domains is converted from an unordered structure into a distorted but spatially fixed helix [22]. In addition to stabilizing RNA∶protein interactions, this rearrangement alters the ability of Sxl to physically interact with other splicing co-factors [40]. If the Orb RRM domains and linker region also undergo similar conformational changes upon RNA binding, this could provide a mechanism for coupling binding to phosphorylation. Since the linker region separating the two RRM domains would be an obvious target for binding dependent conformational changes that could potentially modulate phosphorylation, it is intriguing that a tryptic peptide (which contains two potential CK2 sites) from this linker region is phosphorylated *in vivo*. Unfortunately, we were unable to test this mechanism as we could not generate properly folded wild type (or mutant) Orb protein that had RNA-binding activity.

Another line of evidence arguing that Orb activity is linked to its phosphorylation status is the difference in the spectrum of proteins associated with the hypo- and hyperphosphorylated isoforms. While Me31B and PABP are in complexes with both isoforms, Bruno appears to interact primarily with the “hypophosphorylated” isoforms. This interaction fits with the striking difference in the distribution of Bruno and Orb proteins on sucrose gradients and with the limited co-localization of the two proteins to sponge bodies near the anterior of the oocyte [33], [34]. In contrast to Bruno, the poly(A) polymerase Wisp, which is needed to activate translation, interacts preferentially with the “hyperphosphorylated” isoforms [39]. Although a precursor-product relationship remains to be established for *orb* target mRNAs, this specificity would be consistent with a model in which hypophosphorylated isoforms are in complexes with mRNAs that are translationally repressed. Translational activation would then depend upon phosphorylation of the hypophosphorylated isoforms and reorganization of the Orb complex. Bruno and/or other repressive factors would be displaced from the complex, while the Wisp poly(A) polymerase would be recruited and could potentially begin extending the poly(A) tails. Supporting a model of this type, preliminary studies indicate that like *ck2*, mutations in *wisp* dominantly enhance the *HD19G orb^F343^/+* DV polarity defects, while mutations in the Bruno gene *arrest* have the opposite effect.

The conclusion that the two isoforms are incorporated into complexes that differ substantially in their composition, and likely also their function, is supported by sucrose gradient fractionation of ovary extracts. “Hypophosphorylated” isoforms are found mostly near the top of the gradient in comparatively small complexes (<80S). This region of the gradient is also greatly enriched in the translational repressors Bruno and Me31B, while these repressors are largely absent from the more rapidly sedimenting fractions that contain the polysomes [34], (unpublished data). In contrast, “hyperphosphorylated” isoforms are found in 80S complexes and polysomes. As would be expected if the hyperphosphorylated Orb in these big complexes is directly associated with ribosomes, rather than with some other type of very large RNP, a large collection of ribosomal proteins and translation initiation/elongation factors are found in Orb immunoprecipitates.

### CK2 is required for Orb phosphorylation and function

The kinase most closely tied to CPEB phosphorylation in vertebrates is Aurora [12], which has been shown to phosphorylate the Ser174 residue in the N-terminal half of the *Xenopus* CPEB. However, Aurora's role in Orb phosphorylation is uncertain as this residue is not conserved in Orb and we were also unable to detect any genetic interactions between *aurora* mutations and *orb* (unpublished). Though these results don't exclude a role for Aurora, they suggest that other kinases may phosphorylate Orb. Using a proteomics approach we identified two candidate Orb kinases, SRPK2 and CK2, and here we have focused on CK2.

Like *orb*, *ck2* is needed during oogenesis for the formation of the DV polarity axis of the egg and embryo. Chorion defects characteristic of disruptions in the *grk* signaling pathway are observed in eggs laid by females heterozygous for the dominant negative *ck2α^Tik^* allele or homozygous for the very weak loss-of-function *ck2β^and^* allele. Moreover, the genetic interactions between *ck2* and *orb* in DV polarity would argue that these defects arise, at least in part, because *ck2* is required for *orb* function in this particular signaling pathway. The most compelling of these interactions is between *orb* and the strong loss-of-function *ck2β^mbuΔA26-2L^* allele. Unlike *ck2α^Tik^*, eggs laid by *ck2β^mbuΔA26-2L^/+* females have no apparent DV polarity defects; however, when this mutation is introduced into a background partially compromised for *orb* activity, a very strong interaction is observed and almost three quarters of the eggs laid by *trans*-heterozygous females have chorion defects.

In addition to the genetic interactions in DV polarity, we found that *ck2* has a direct impact on *orb* autoregulation. Orb is required for localizing and activating the on-site translation of *orb* mRNA in the developing oocyte. Strikingly, both of these autoregulatory activities are disrupted in females that are only partially compromised for *ck2*. *orb* mRNA is not properly localized in vitellogenic stage egg chambers. In addition, the accumulation of Orb protein is reduced compared to wild type. Since the females harboring these *ck2* mutant combinations are viable and morphologically normal, it would appear that like DV polarity, these *orb* regulatory activities are especially sensitive to reductions in *ck2* activity.

The effects of *ck2* on *orb* function correlate with changes in the phosphoisoform profile. In *ck2* backgrounds that have modest effects on polarity and/or *orb* activity, there is a small shift towards the hypophosphorylated isoforms. In backgrounds that have stronger effects on *orb* activity and/or show synergistic genetic interactions with *orb*, the changes in phosphoisoform profile are more pronounced. This is, perhaps, most evident in females heterozygous for the amorphic *ck2β^mbuΔA26-2L^* allele. In addition to the hypo- and hyperphosphorylated isoforms visible on regular SDS-PAGE gels, these females have an Orb species that has a similar mobility to dephosphorylated Orb after λ phosphatase-treatment.

While these findings indicate that *ck2* is required both for *orb* activity and to generate the normal array of Orb phosphoisoforms, the fact that this kinase has been implicated in many cellular processes raises the possibility that the effects on *orb* are an indirect consequence of pleiotropic defects in oogenesis induced by *ck2* mutations. Unfortunately, this possibility can not be excluded; however, arguing against it is the fact that all of our experiments were done under conditions in which *ck2* activity is only partially compromised. Though this might not eliminate pleiotropic effects, it should certainly minimize them and at the same time reveal cellular processes that are especially sensitive to reductions in *ck2* activity and thus most likely to be intimately connected to *ck2* function. DV polarity, *orb* activity and Orb phosphorylation would fit into this category. As for how *ck2* impacts *orb* activity and the Orb phosophoisoform profile, the simplest explanation is that it is directly responsible for phosphorylating Orb. Consistent with this idea, there are twelve potential CK2 sites, of which nine are conserved even in distantly related *Drosophila* species. Of the conserved sites, two are in the linker region separating the two Orb RRM domains and, as mentioned above, could be potential candidates for RNA binding dependent phosphorylation. The seven remaining sites are in short conserved sequence blocks in the otherwise poorly conserved N-terminal half of the Orb protein. Interestingly, six of these are in a closely spaced cluster (see Fig. 2A). The physical association between CK2 and Orb would also support a direct mechanism. On the other hand, it is also possible that *ck2* acts indirectly through intermediate kinases. In this case, the activity of this kinase cascade would have to be especially sensitive to changes in *ck2* levels. However, even in this indirect scenario, the effects of *ck2* mutations on *orb* activity and the phosphoisoform profile provide further evidence linking the regulatory functions of the Orb protein to its phosphorylation status.

Whether the effects of *ck2* on *orb* are direct or indirect, there are indications that other kinases must phosphorylate Orb. For one, there are likely to be phosphorylated tyrosine residues since the Orb phosphoisoforms are not completely collapsed by the Ser/Thr specific Protein Phosphatase 1, but are collapsed by λ phosphatase. Consistent with this possibility, preliminary experiments indicate that Orb is recognized by phosphotyrosine antibodies. Secondly, other Ser/Thr kinases might be needed to activate Orb. Studies by Barbosa *et al.*, [41] demonstrate that *srpk2* mutations disrupt oogenesis and have DV polarity phenotypes. Though the reported phenotypes seem different from those of well-characterized *orb* mutants, we found that a P-element-induced mutation in *srpk2* dominantly enhanced the *orb* DV polarity defects. Finally, since known *orb* target mRNAs exhibit different patterns of localization and translation, they are likely to be associated with a unique set of regulatory proteins and depend upon different signaling cascades for translational activation. Thus, a more likely scenario is that CK2 is just one of several potential Orb kinases, and that translation of different *orb* target mRNAs might require the deployment of specific constellations of modifying enzymes.

## Materials and Methods

### Phosphatase assay

Wild type and *orb^F303^* ovaries were dissected in 1× PBS and homogenized in the presence of protease and phosphatase inhibitors (Roche). An aliquot of the resulting supernatant was set aside for the non-phosphatase-treated control, while the experimental sample was incubated with λ protein phosphatase, YOP phosphatase or PP1 (NEBiolabs) for 1–2 hrs at 30°C.

### Sequence analysis of *orb^F303^* molecular lesion

The coding sequence of the ovarian isoform of the *orb* gene was determined for wild type, *orb^F303^* and *orb^F343^* flies. Total RNA was extracted from ovaries and reverse transcribed using random primers. The *orb* coding region was PCR amplified from the cDNAs and cloned using primers that generate overlapping fragments of 200–500 bp. At least six independent clones were sequenced for each fragment. The *orb^F303^* sequence was compared with wild type and *orb^F343^*.

### *Drosophila* stocks and genetic interaction assay

*HD19G orb^F343^/Tm3Ser* flies were described in [20]. *ck2α^Tik^* and *ck2α^TikR^* alleles used in this study were gifts from Ravi Allada [31]. *ck2β^mbuΔA26-2L^* and *ck2β^And^* were generated in Rob Jackson's lab [32].

## Supporting Information

Figure S1 **A mutation in the second RRM domain in** ***orb^F303^*** **abolishes the hyperphosphorylated isoform.** Sequence analysis of *orb^F303^* cDNA indicates that the EMS-induced mutation changes a thymidine (T) residue at the first position of codon 742 to an adenosine (A) residue. This nucleotide substitution alters the codon so that instead of specifying tyrosine (Y) it encodes an asparagine (N) residue. Tyrosine742 is located.
(DOC)

Figure S2 **CK2 associates with Orb** ***in vivo*** **.** Ovary extracts were immunoprecipitated with antibodies against CK2 (CK2 IP) or β-galactosidase proteins (β-gal IP), and probed with antibodies against Orb. The amount of extract (Ex) loaded in the first lane is 10% of the amount of extract used for immunoprecipitation. The IP lanes represent approximately 30% of the input extract. Rabbit antibodies against the CK2 holoenzyme were a gift from A.P. Bidwai. {Karandikar U.C., Shaffer J., Bishop C.P., and Bidwai A.P. (2005) *Drosophila* Ck2 phosphorylates Deadpan, a member of the HES family of basic-helix-loop-helix (bHLH) repressors. Mol Cell Biochem. *274*, 133–139.}
(DOC)

Figure S3 **Predicted CK2 phosphorylation sites in the Orb protein.** Diagram shows the position of the predicted CK2 phosphorylation sites in Orb [30]. Two of these are located in the C terminal half of the Orb protein, in the linker region separating the two RRM domains. The remaining sites are in the poorly conserved N-terminal half of the Orb protein. Also shown here are the amino acid sequences from *melanogaster* and four other *Drosophila* species in the immediate vicinity of predicted CK2 phosphorylation sites.
(DOC)

Figure S4 **Orb phosphorylation pattern is altered in** ***ck2*** **mutants.** Orb protein from wild type ovaries can be resolved into 7 or more hypo- and hyperphosphorylated isoforms using Phos-Tag SDS-PAGE gel electrophoresis (WT; 1–7). The phospho-isoforms showing the greatest retardation in this gel system are expected to have the greatest number of phosphorylated amino residues. Note that in this particular gel several of the bands seen in Fig. 1D appear to be doublets (bands 3, 4 and 7). Analysis of the Orb protein from ovaries from *trans*-heterozygous *ck2α^Tik^/ckβ^mbuΔA26-2L^* (*Tik/mbu*) and *ckβ^And^/ckβ^mbuΔA26-2L^* (*And/mbu*) females using the Phos-Tag SDS-PAGE gels reveals a substantially altered phosphorylation pattern. As observed in standard SDS-PAGE gels, there is a marked reduction in the hyperphosphorylated isoforms, and bands 5–7 are largely missing in ovaries compromised for *ck2* activity. There is also a shift in the pattern of hypophosphorylated isoforms. The most striking change is the increase in the band 1, which based on phosphatase experiments, corresponds to an unphosphorylated Orb isoform. The appearance of this unphosphorylated isoform was also seen in standard SDS-PAGE gels. In addition, there is also a reduction in the levels of the hypophosphorylated bands 3 and 4.
(DOC)

Figure S5 ***orb*** **mRNA localization is disrupted when** ***ck2*** **activity is compromised.** In wild type *orb* mRNA expressed in nurse cells of stage 8–10 chambers is localized along the anterior margin of the oocyte (arrow in panel A). In *ck2α^Tik^/ck2β^And^* chambers *orb* mRNA does not accumulate to high levels along anterior margin (arrows in panel B–D).
(DOC)

Table S1 **Mass spectrometry analysis of proteins present in Orb and Dorsal immunoprecipitations of ovary extracts.** Ovary extracts were immunoprecipiated with Orb and Dorsal antibodies as previously described [26]. The immunopreciiptated samples were then analyzed as described in [24] and [24].
(DOC)
