# Supplementary material for: The Functioning of the Drosophila CPEB Protein Orb Is Regulated by Phosphorylation and Requires Casein Kinase 2 Activity
Source: PLoS One. 2011 Sep 19;6(9):e24355. doi: 10.1371/journal.pone.0024355 (PMC3176278; doi:10.1371/journal.pone.0024355)
Supplement: Figure S1 — A mutation in the second RRM domain in orbF303 abolishes the hyperphosphorylated isoform. Sequence analysis of orbF303 cDNA indicates that the EMS-induced mutation changes a thymidine (T) residue at the first position of codon 742 to an adenosine (A) residue. This nucleotide substitution alters the codon so that instead of specifying tyrosine (Y) it encodes an asparagine (N) residue. Tyrosine742 is located. (DOC) [file pone.0024355.s001.doc]

**Figure S1: A mutation in the second RRM domain in *orb^F303^*abolishes the hyperphosphorylated isoform.** Sequence analysis of *orb^F303^* cDNA indicates that the EMS-induced mutation changes a thymidine (T) residue at the first position of codon 742 to an adenosine (A) residue. This nucleotide substitution alters the codon so that instead of specifying tyrosine (Y) it encodes an asparagine (N) residue. Tyrosine742 is located
