# Supplementary material for: The Functioning of the Drosophila CPEB Protein Orb Is Regulated by Phosphorylation and Requires Casein Kinase 2 Activity
Source: PLoS One. 2011 Sep 19;6(9):e24355. doi: 10.1371/journal.pone.0024355 (PMC3176278; doi:10.1371/journal.pone.0024355)
Supplement: Figure S2 — CK2 associates with Orb in vivo . Ovary extracts were immunoprecipitated with antibodies against CK2 (CK2 IP) or β-galactosidase proteins (β-gal IP), and probed with antibodies against Orb. The amount of extract (Ex) loaded in the first lane is 10% of the amount of extract used for immunoprecipitation. The IP lanes represent approximately 30% of the input extract. Rabbit antibodies against the CK2 holoenzyme were a gift from A.P. Bidwai. {Karandikar U.C., Shaffer J., Bishop C.P., and Bidwai A.P. (2005) Drosophila Ck2 phosphorylates Deadpan, a member of the HES family of basic-helix-loop-helix (bHLH) repressors. Mol Cell Biochem. 274, 133–139.} (DOC) [file pone.0024355.s002.doc]

**Figure S2**: **CK2 associates with Orb *in vivo*.** Ovary extracts were immunoprecipitated with antibodies against CK2 (CK2 IP) or β-galactosidase proteins (β-gal IP), and probed with antibodies against Orb. The amount of extract (Ex) loaded in the first lane is 10% of the amount of extract used for immunoprecipitation. The IP lanes represent approximately 30% of the input extract. Rabbit antibodies against the CK2 holoenzyme were a gift from A.P. Bidwai. {Karandikar U.C., Shaffer J., Bishop C.P., and Bidwai A.P. (2005) *Drosophila* Ck2 phosphorylates Deadpan, a member of the HES family of basic-helix-loop-helix (bHLH) repressors. Mol Cell Biochem. *274*, 133-139.}
