# Supplementary material for: The Functioning of the Drosophila CPEB Protein Orb Is Regulated by Phosphorylation and Requires Casein Kinase 2 Activity
Source: PLoS One. 2011 Sep 19;6(9):e24355. doi: 10.1371/journal.pone.0024355 (PMC3176278; doi:10.1371/journal.pone.0024355)
Supplement: Figure S3 — Predicted CK2 phosphorylation sites in the Orb protein. Diagram shows the position of the predicted CK2 phosphorylation sites in Orb [30]. Two of these are located in the C terminal half of the Orb protein, in the linker region separating the two RRM domains. The remaining sites are in the poorly conserved N-terminal half of the Orb protein. Also shown here are the amino acid sequences from melanogaster and four other Drosophila species in the immediate vicinity of predicted CK2 phosphorylation sites. (DOC) [file pone.0024355.s003.doc]

**Map of Orb protein and putative CK2 phosphorylation sites.**

**Sequence Conservation of CK2 phosphorylation Sites.**

**C-terminal domain between RRM1 and RRM2**

**Linker between RRM1 and RRM2**

melanogaster 667- IPWIIADSNFVRSSSQKLDPTK-688| **RRM2**

yakuba 777- IPWIIADSNFVRSSSQKLDPTk-898

willistoni 649- IPWIIADSNFVRSSSQKLDPTK-670

mojavensis 771- IPWIIADSN*Y*VRSSSQKLDPTK-792

virilis 755- IPWIIADSN*Y*VRSSSQKLDPTK-778

Aedes aegypti 631- IPWNIADSNYVKSTSQKLDPTK-654

**N-terminal domain**

**Site 35**

Melanogaster 20- RALSGGSTTELLQKHSISSYLDHH-43

yakubua 26- RALSGGSTTELLQKHSISSYLDHH-49

willistoni 17- RSLNDGNPSDLLQKHSINSLLEHQ-40

mojavensis 20- RALSGASASDLLQKHSISSILEQH-43

virilis 20- RALSGASANDLLQKHSISSILEQH-43

Aedes aegypti 22- RSLTG---PDILQKHSINSLLLEH-42

**Site 183**

melanogaster 169- SAANPSASFGGNGSSSDVNNLLLA-193

yakubua 287- SAANPSASFGGNGSSSDVNNLLLA-310

willistoni 133- MTNSSVCVSGNGSSSGGVNNLLLA-157

mojavensis 240- AAGIASGAGGNGSSSSSGVNNLLL-254

virilis 213- AAGIASGAGGNGSSSSSGVNNLLL-236

Aedes aegypti sequence not conserved

**Site 286**

melanogaster 279- FHSSPLVSDSSNSSS-293

yakubua 396- FHSSPLVSDSSNSSS-410

willistoni 245- FHSSPLVSDSSNSSS-259

mojavensis 344- FHSSPLVSDSSNSSS-358

virilis 333- FHSSPLVSDSSNSSS-347

Aedes aegypti 76- SSSLGASSSSNSNS-89

**Sites 342, 344, 347, 350,351 and 353**

melanogaster 334- LANASTRSNSPESQNSSNSTTEQNLLDM-362

yakuba 451- LANASTRSNSPESQNSSQSTTEPNLLDM-478

willistoni 315- LPNASTRSNSPESQNSNQSINETNLLDM-342

mojavensis 394- IQNASTRSNSPESQNSNQSFNEPNLLDM-419

virilis 373- LQNASTRSNSPESQNSNQSFNEPNLLDM-400

Aedes aegypti 278- RSNSPPDNDQSSLLYNFDSA---NILDM-302

**Site 444**

melanogaster 429- HGFEHNGVGVGASSSGNENCFSQY-453

yakuba 545- NGYEHNGVGVGASRG---DCLSQY-564

willistoni 494- VGVGGSGS -501

mojavensis no similarity

virilis no similarity

**Figure S3: Predicted CK2 phosphorylation sites in the Orb protein.** Diagram shows the position of the predicted CK2 phosphorylation sites in Orb [30]. Two of these are located in the C terminal half of the Orb protein, in the linker region separating the two RRM domains. The remaining sites are in the poorly conserved N-terminal half of the Orb protein. Also shown here are the amino acid sequences from *melanogaster* and four other *Drosophila* species in the immediate vicinity of predicted CK2 phosphorylation sites.
