# Supplementary material for: The Functioning of the Drosophila CPEB Protein Orb Is Regulated by Phosphorylation and Requires Casein Kinase 2 Activity
Source: PLoS One. 2011 Sep 19;6(9):e24355. doi: 10.1371/journal.pone.0024355 (PMC3176278; doi:10.1371/journal.pone.0024355)
Supplement: Figure S4 — Orb phosphorylation pattern is altered in ck2 mutants. Orb protein from wild type ovaries can be resolved into 7 or more hypo- and hyperphosphorylated isoforms using Phos-Tag SDS-PAGE gel electrophoresis (WT; 1–7). The phospho-isoforms showing the greatest retardation in this gel system are expected to have the greatest number of phosphorylated amino residues. Note that in this particular gel several of the bands seen in Fig. 1D appear to be doublets (bands 3, 4 and 7). Analysis of the Orb protein from ovaries from trans-heterozygous ck2αTik/ckβmbuΔA26-2L (Tik/mbu) and ckβAnd/ckβmbuΔA26-2L (And/mbu) females using the Phos-Tag SDS-PAGE gels reveals a substantially altered phosphorylation pattern. As observed in standard SDS-PAGE gels, there is a marked reduction in the hyperphosphorylated isoforms, and bands 5–7 are largely missing in ovaries compromised for ck2 activity. There is also a shift in the pattern of hypophosphorylated isoforms. The most striking change is the increase in the band 1, which based on phosphatase experiments, corresponds to an unphosphorylated Orb isoform. The appearance of this unphosphorylated isoform was also seen in standard SDS-PAGE gels. In addition, there is also a reduction in the levels of the hypophosphorylated bands 3 and 4. (DOC) [file pone.0024355.s004.doc]

**Figure S4: Orb phosphorylation pattern is altered in *ck2* mutants.** Orb protein from wild type ovaries can be resolved into 7 or more hypo- and hyperphosphorylated isoforms using Phos-Tag SDS-PAGE gel electrophoresis (WT; 1-7). The phospho-isoforms showing the greatest retardation in this gel system are expected to have the greatest number of phosphorylated amino residues. Note that in this particular gel several of the bands seen in Fig. 1D appear to be doublets (bands 3, 4 and 7). Analysis of the Orb protein from ovaries from *trans*-heterozygous *ck2α^Tik^/ckβ^mbuΔA26-2L^* (*Tik/mbu*) and *ckβ^And^/ckβ^mbuΔA26-2L^* (*And/mbu*) females using the Phos-Tag SDS-PAGE gels reveals a substantially altered phosphorylation pattern. As observed in standard SDS-PAGE gels, there is a marked reduction in the hyperphosphorylated isoforms, and bands 5-7 are largely missing in ovaries compromised for *ck2* activity. There is also a shift in the pattern of hypophosphorylated isoforms. The most striking change is the increase in the band 1, which based on phosphatase experiments, corresponds to an unphosphorylated Orb isoform. The appearance of this unphosphorylated isoform was also seen in standard SDS-PAGE gels. In addition, there is also a reduction in the levels of the hypophosphorylated bands 3 and 4.
