# Supplementary material for: The Functioning of the Drosophila CPEB Protein Orb Is Regulated by Phosphorylation and Requires Casein Kinase 2 Activity
Source: PLoS One. 2011 Sep 19;6(9):e24355. doi: 10.1371/journal.pone.0024355 (PMC3176278; doi:10.1371/journal.pone.0024355)
Supplement: Figure S5 — orb mRNA localization is disrupted when ck2 activity is compromised. In wild type orb mRNA expressed in nurse cells of stage 8–10 chambers is localized along the anterior margin of the oocyte (arrow in panel A). In ck2αTik/ck2βAnd chambers orb mRNA does not accumulate to high levels along anterior margin (arrows in panel B–D). (DOC) [file pone.0024355.s005.doc]

**Figure S5: *orb* mRNA localization is disrupted when *ck2* activity is compromised.**  In wild type *orb* mRNA expressed in nurse cells of stage 8-10 chambers is localized along the anterior margin of the oocyte (arrow in panel A). In *ck2α^Tik^/ ck2β^And^* chambers *orb* mRNA does not accumulate to high levels along anterior margin (arrows in panel B-D).
