# Supplementary material for: The Functioning of the Drosophila CPEB Protein Orb Is Regulated by Phosphorylation and Requires Casein Kinase 2 Activity
Source: PLoS One. 2011 Sep 19;6(9):e24355. doi: 10.1371/journal.pone.0024355 (PMC3176278; doi:10.1371/journal.pone.0024355)
Supplement: Table S1 — Mass spectrometry analysis of proteins present in Orb and Dorsal immunoprecipitations of ovary extracts. Ovary extracts were immunoprecipiated with Orb and Dorsal antibodies as previously described [26]. The immunopreciiptated samples were then analyzed as described in [24] and [24]. (DOC) [file pone.0024355.s006.doc]

**Table S1: Mass spectrometry analysis of proteins present in Orb and Dorsal immunoprecipitations of ovary extracts.**

Orb IP

| Locus | Orb IP | Dorsal IP | Orb IP | Dorsal IP | Total | Description |
| --- | --- | --- | --- | --- | --- | --- |
| 143E_DROME | [11.8](../orb_02-updated_search.html#143E_DROME) |  | [11.8](../orb_03-phos.html#143E_DROME) |  | [11.8](http://gemini.scripps.edu/cgi-bin/SeqCov?/scratch/yates/FlyBase_Drosophila_na_02-27-2005_con_reversed.fasta&143E_DROME&95+12^197+19&95+12^197+19*) | (P92177) 14-3-3 protein epsilon (Suppressor of Ras1 3-9) |
| **AAF48092**  **KC2B_DROME**  **AAS65321**  **AAF48093** | **[31.0](../orb_02-updated_search.html#AAF48092)** |  | **[23.5](../orb_03-phos.html#AAF48092)** |  | [**31.0**](http://gemini.scripps.edu/cgi-bin/SeqCov?/scratch/yates/FlyBase_Drosophila_na_02-27-2005_con_reversed.fasta&AAF48092&29+30^123+23^203+17&29+30^123+23*)  [**32.6**](http://gemini.scripps.edu/cgi-bin/SeqCov?/scratch/yates/FlyBase_Drosophila_na_02-27-2005_con_reversed.fasta&KC2B_DROME&18+30^112+23^192+17&18+30^112+23*)  [**29.8**](http://gemini.scripps.edu/cgi-bin/SeqCov?/scratch/yates/FlyBase_Drosophila_na_02-27-2005_con_reversed.fasta&AAS65321&18+30^112+23^192+17&18+30^112+23*)  [**32.6**](http://gemini.scripps.edu/cgi-bin/SeqCov?/scratch/yates/FlyBase_Drosophila_na_02-27-2005_con_reversed.fasta&AAF48093! &18+30^112+23^192+17&18+30^112+23*) | **(AAF48092) CG15224-PA**  **(P08182) Casein kinase II beta chain (CK II)**  **(AAS65321) CG15224-PE**  **(AAF48093) CG15224-PB** |
| AAF51926  RM62_DROME | [29.8](../orb_02-updated_search.html#AAF51926) |  | [30.4](../orb_03-phos.html#AAF51926) |  | [30.4](http://gemini.scripps.edu/cgi-bin/SeqCov?/scratch/yates/FlyBase_Drosophila_na_02-27-2005_con_reversed.fasta&AAF51926&78+25^123+10^162+24^187+57^274+13^377+15^460+17^510+15&78+25^123+10^162+24^191+53^274+13^377+15^460+17^510+15&191+20^460+17&191+20&191+20*)  [24.5](http://gemini.scripps.edu/cgi-bin/SeqCov?/scratch/yates/FlyBase_Drosophila_na_02-27-2005_con_reversed.fasta&RM62_DROME&219+25^264+10^303+24^328+57^415+13^518+15^601+17^651+15&219+25^264+10^303+24^332+53^415+13^518+15^601+17^651+15&332+20^601+17&332+20&332+20*) | (AAF51926) CG10279-PC  (P19109) Putative ATP-dependent RNA helicase P62 |
| AAF54180  Q9VHW9  AAG22133 | [3.4](../orb_02-updated_search.html#AAF54180) |  | [3.4](../orb_03-phos.html#AAF54180) |  | [3.4](http://gemini.scripps.edu/cgi-bin/SeqCov?/scratch/yates/FlyBase_Drosophila_na_02-27-2005_con_reversed.fasta&AAF54180&22+10^163+14^1424+11^1660+12^1958+12^2040+12&22+10^163+14^1424+11^1660+12^1958+12^2040+12&2040+12&2040+12*)  [3.4](http://gemini.scripps.edu/cgi-bin/SeqCov?/scratch/yates/FlyBase_Drosophila_na_02-27-2005_con_reversed.fasta&Q9VHW9&22+10^163+14^1424+11^1660+12^1958+12^2040+12&22+10^163+14^1424+11^1660+12^1958+12^2040+12&2040+12&2040+12*)  [3.3](http://gemini.scripps.edu/cgi-bin/SeqCov?/scratch/yates/FlyBase_Drosophila_na_02-27-2005_con_reversed.fasta&AAG22133&70+10^211+14^1472+11^1708+12^2006+12^2088+12&70+10^211+14^1472+1! 1^1708+12^2006+12^2088+12&2088+12&2088+12*) | (AAF54180) CG2747-PB  (Q9VHW9) CG2747 protein  (AAG22133) CG2747-PA |
| AAF54303 | [3.1](../orb_02-updated_search.html#AAF54303) |  | [3.1](../orb_03-phos.html#AAF54303) |  | [3.1](http://gemini.scripps.edu/cgi-bin/SeqCov?/scratch/yates/FlyBase_Drosophila_na_02-27-2005_con_reversed.fasta&AAF54303&80+25^370+11&80+25^370+11*) | (AAF54303) CG31349-PE |
| AAF54520 | [15.2](../orb_02-updated_search.html#AAF54520) |  | [14.8](../orb_03-phos.html#AAF54520) |  | 15.5 | (AAF54520) CG6303-PA |
| AAF54965 | [67.2](../orb_02-updated_search.html#AAF54965) |  | [65.7](../orb_03-phos.html#AAF54965) |  | [67.2](http://gemini.scripps.edu/cgi-bin/SeqCov?/scratch/yates/FlyBase_Drosophila_na_02-27-2005_con_reversed.fasta&AAF54965&2+17^23+6^31+146^179+60^322+50^377+50^482+42^538+45^591+14^629+20^676+14&2+17^23+6^31+146^179+60^322+50^377+50^482+42^538+45^591+14^629+20^676+14&31+21^68+61^190+29^332+30^391+35^482+42^538+18^558+16^629+9^676+14&31+21^68+7^85+44^190+29^332+18^400+19^482+29^516+8^541+15^558+16^629+9&42+10^85+44^192+27^341+9^482+29^541+15^564+10^629+9*) | (AAF54965) CG9412-PB |
| AAF55259  Q9VF03 | [31.9](../orb_02-updated_search.html#AAF55259) |  | [31.9](../orb_03-phos.html#AAF55259) |  | 31.9  31.4 | (AAF55259) CG18740-PA  (Q9VF03) MOIRA protein (Brahma associated protein 155 kDa) (Brahma-associated putative chromatin-remodeling factor) |
| AAF58773  Q9V5M8  Q9V5M6  Q9V5M5  Q9V5M3  Q9V5M2  Q95TS0  Q8MKX6  Q8MKX5  Q8MKX4  Q86BC4  Q86BC3  Q86BC2  Q867Z4  Q867S9  Q867R1  Q867K2  LOLL_DROME  AAS64875  AAS64874  AAF58777 | [3.4](../orb_02-updated_search.html#AAF58773) |  | [3.4](../orb_03-phos.html#AAF58773) |  | [3.4](http://gemini.scripps.edu/cgi-bin/SeqCov?/scratch/yates/FlyBase_Drosophila_na_02-27-2005_con_reversed.fasta&AAF58773&20+20^68+7&20+20^68+7*)  [3.4](http://gemini.scripps.edu/cgi-bin/SeqCov?/scratch/yates/FlyBase_Drosophila_na_02-27-2005_con_reversed.fasta&Q9V5M8&20+20^68+7&20+20^68+7*)  [2.9](http://gemini.scripps.edu/cgi-bin/SeqCov?/scratch/yates/FlyBase_Drosophila_na_02-27-2005_con_reversed.fasta&Q9V5M6&20+20^68+7&20+20^68+7*)  [2.9](http://gemini.scripps.edu/cgi-bin/SeqCov?/scratch/yates/FlyBase_Drosophila_na_02-27-2005_con_reversed.fasta&Q9V5M5&20+20^68+7&20+20^68+7*)  [2.8](http://gemini.scripps.edu/cgi-bin/SeqCov?/scratch/yates/FlyBase_Drosophila_na_02-27-2005_con_reversed.fasta&Q9V5M3&20+20^68+7&20+20^68+7*)  [3.0](http://gemini.scripps.edu/cgi-bin/SeqCov?/scratch/yates/FlyBase_Drosophila_na_02-27-2005_con_reversed.fasta&Q9V5M2&20+20^68+7&20+20^68+7*)  [3.6](http://gemini.scripps.edu/cgi-bin/SeqCov?/scratch/yates/FlyBase_Drosophila_na_02-27-2005_con_reversed.fasta&Q95TS0&20+20^68+7&20+20^68+7*)  [4.5](http://gemini.scripps.edu/cgi-bin/SeqCov?/scratch/yates/FlyBase_Drosophila_na_02-27-2005_con_reversed.fasta&Q8MKX6&20+20^68+7&20+20^68+7*)  [3.8](http://gemini.scripps.edu/cgi-bin/SeqCov?/scratch/yates/FlyBase_Drosophila_na_02-27-2005_con_reversed.fasta&Q8M! KX5&20+20^68+7&20+20^68+7*)  [4.5](http://gemini.scripps.edu/cgi-bin/SeqCov?/scratch/yates/FlyBase_Drosophila_na_02-27-2005_con_reversed.fasta&Q8MKX4&20+20^68+7&20+20^68+7*)  [4.0](http://gemini.scripps.edu/cgi-bin/SeqCov?/scratch/yates/FlyBase_Drosophila_na_02-27-2005_con_reversed.fasta&Q86BC4&20+20^68+7&20+20^68+7*)  [3.2](http://gemini.scripps.edu/cgi-bin/SeqCov?/scratch/yates/FlyBase_Drosophila_na_02-27-2005_con_reversed.fasta&Q86BC3&20+20^68+7&20+20^68+7*)  [3.8](http://gemini.scripps.edu/cgi-bin/SeqCov?/scratch/yates/FlyBase_Drosophila_na_02-27-2005_con_reversed.fasta&Q86BC2&20+20^68+7&20+20^68+7*)  [2.8](http://gemini.scripps.edu/cgi-bin/SeqCov?/scratch/yates/FlyBase_Drosophila_na_02-27-2005_con_reversed.fasta&Q867Z4&20+20^68+7&20+20^68+7*)  [4.4](http://gemini.scripps.edu/cgi-bin/SeqCov?/scratch/yates/FlyBase_Drosophila_na_02-27-2005_con_reversed.fasta&Q867S9&20+20^68+7&20+20^! 68+7*)  [5.8](http://gemini.scripps.edu/cgi-bin/SeqCov?/scratch/yates/FlyBase_Drosophila_na_02-27-2005_con_reversed.fasta&Q867R1&20+20^68+7&20+20^68+7*)  [4.8](http://gemini.scripps.edu/cgi-bin/SeqCov?/scratch/yates/FlyBase_Drosophila_na_02-27-2005_con_reversed.fasta&Q867K2&20+20^68+7&20+20^68+7*)  [3.0](http://gemini.scripps.edu/cgi-bin/SeqCov?/scratch/yates/FlyBase_Drosophila_na_02-27-2005_con_reversed.fasta&LOLL_DROME&20+20^68+7&20+20^68+7*)  [4.7](http://gemini.scripps.edu/cgi-bin/SeqCov?/scratch/yates/FlyBase_Drosophila_na_02-27-2005_con_reversed.fasta&AAS64875&20+20^68+7&20+20^68+7*)  [4.5](http://gemini.scripps.edu/cgi-bin/SeqCov?/scratch/yates/FlyBase_Drosophila_na_02-27-2005_con_reversed.fasta&AAS64874&20+20^68+7&20+20^68+7*)  [5.2](http://gemini.scripps.edu/cgi-bin/SeqCov?/scratch/yates/FlyBase_Drosophila! _na_02-27-2005_con_reversed.fasta&AAF58777&20+20^68+7&20+20^68+7*) | (AAF58773) CG12052-PC  (Q9V5M8) Lola protein (BTB-IV protein domain)  (Q9V5M6) CG12052-PP  (Q9V5M5) CG12052-PJ  (Q9V5M3) CG12052-PN  (Q9V5M2) CG12052 protein  (Q95TS0) LD28033p (CG12052-PB) (Lola protein isoform F)  (Q8MKX6) CG12052-PK  (Q8MKX5) CG12052-PA (LD03274p)  (Q8MKX4) CG12052-PQ  (Q86BC4) CG12052-PO  (Q86BC3) CG12052-PR  (Q86BC2) CG12052-PS  (Q867Z4) CG12052-PI (Lola protein isoform K)  (Q867S9) CG12052-PL (Lola protein isoform C)  (Q867R1) CG12052-PM (Lola protein isoform A)  (Q867K2) CG12052-PF (Lola protein isoform I)  (P42283) Lola protein, long isoform (Longitudinals lacking protein)  (AAS64875) CG12052-PY  (AAS64874) CG12052-PX  (AAF58777) CG12052-PH |
| AAN12148 | [16.7](../orb_02-updated_search.html#AAN12148) |  | [15.6](../orb_03-phos.html#AAN12148) |  | [16.7](http://gemini.scripps.edu/cgi-bin/SeqCov?/scratch/yates/FlyBase_Drosophila_na_02-27-2005_con_reversed.fasta&AAN12148&12+19^456+29^747+16^771+16^1008+30^1161+31^1225+30^1270+41^1335+29^1603+13^1685+36^1792+16^1839+19^1953+12^1966+15^2001+26^2203+14^2373+46&12+19^456+29^747+16^771+16^1008+30^1161+31^1225+30^1270+41^1603+13^1685+36^1792+16^1839+19^1953+12^1966+15^2001+26^2203+14^2373+46&1238+17^1282+29^1966+15&1238+17^1282+29^1966+15*) | (AAN12148) CG9936-PC |
| AAS64933  Q9W093 | [5.0](../orb_02-updated_search.html#AAS64933) |  | [5.0](../orb_03-phos.html#AAS64933) |  | [5.0](http://gemini.scripps.edu/cgi-bin/SeqCov?/scratch/yates/FlyBase_Drosophila_na_02-27-2005_con_reversed.fasta&AAS64933&83+35^803+21&83+35^803+21*)  [4.3](http://gemini.scripps.edu/cgi-bin/SeqCov?/scratch/yates/FlyBase_Drosophila_na_02-27-2005_con_reversed.fasta&Q9W093&83+35^997+21&83+35^997+21*) | (AAS64933) CG13921-PB  (Q9W093) CG13921-PA |
| AAS64957  Q9VZQ3  AAS64958 | [1.3](../orb_02-updated_search.html#AAS64957) |  | [0.9](../orb_03-phos.html#AAS64957) |  | [1.8](http://gemini.scripps.edu/cgi-bin/SeqCov?/scratch/yates/FlyBase_Drosophila_na_02-27-2005_con_reversed.fasta&AAS64957&1075+19^1666+16^2229+18^3327+22&1666+16*)  [1.8](http://gemini.scripps.edu/cgi-bin/SeqCov?/scratch/yates/FlyBase_Drosophila_na_02-27-2005_con_reversed.fasta&Q9VZQ3&1075+19^1666+16^2229+18^3327+22&1666+16*)  [1.8](http://gemini.scripps.edu/cgi-bin/SeqCov?/scratch/yates/FlyBase_Drosophila_na_02-27-2005_con_reversed.fasta&AAS64958&1075+19^1666+16^2229+18^3327+22&1666+16*) | (AAS64957) CG12008-PC  (Q9VZQ3) KST protein (Beta-H spectrin)  (AAS64958) CG12008-PB |
| AAS64963  ENC_DROME | [2.2](../orb_02-updated_search.html#AAS64963) |  | [2.2](../orb_03-phos.html#AAS64963) |  | [2.2](http://gemini.scripps.edu/cgi-bin/SeqCov?/scratch/yates/FlyBase_Drosophila_na_02-27-2005_con_reversed.fasta&AAS64963&644+23^1765+17&644+23^1765+17*)  [2.2](http://gemini.scripps.edu/cgi-bin/SeqCov?/scratch/yates/FlyBase_Drosophila_na_02-27-2005_con_reversed.fasta&ENC_DROME&639+23^1760+17&639+23^1760+17*) | (AAS64963) CG10847-PA  (Q8MSX1) Encore protein |
| AAS65065  Q9VS62 | [6.0](../orb_02-updated_search.html#AAS65065) |  | [6.0](../orb_03-phos.html#AAS65065) |  | [6.0](http://gemini.scripps.edu/cgi-bin/SeqCov?/scratch/yates/FlyBase_Drosophila_na_02-27-2005_con_reversed.fasta&AAS65065&318+12^597+19^810+20&318+12^597+19^810+20*)  [5.4](http://gemini.scripps.edu/cgi-bin/SeqCov?/scratch/yates/FlyBase_Drosophila_na_02-27-2005_con_reversed.fasta&Q9VS62&412+12^691+19^904+20&412+12^691+19^904+20*) | (AAS65065) CG8571-PB  (Q9VS62) SMID protein |
| AAS65129  OSKA_DROME | [25.4](../orb_02-updated_search.html#AAS65129) |  | [25.4](../orb_03-phos.html#AAS65129) |  | [25.4](http://gemini.scripps.edu/cgi-bin/SeqCov?/scratch/yates/FlyBase_Drosophila_na_02-27-2005_con_reversed.fasta&AAS65129&24+17^87+12^101+16^179+44^353+12^375+18&24+17^87+12^101+16^179+44^353+12^375+18*)  [19.6](http://gemini.scripps.edu/cgi-bin/SeqCov?/scratch/yates/FlyBase_Drosophila_na_02-27-2005_con_reversed.fasta&OSKA_DROME&162+17^225+12^239+16^317+44^491+12^513+18&162+17^225+12^239+16^317+44^491+12^513+18*) | (AAS65129) CG10901-PC  (P25158) Maternal effect protein oskar |
| AAS65141  Q9VGG9 | [2.2](../orb_02-updated_search.html#AAS65141) |  | [2.8](../orb_03-phos.html#AAS65141) |  | [2.8](http://gemini.scripps.edu/cgi-bin/SeqCov?/scratch/yates/FlyBase_Drosophila_na_02-27-2005_con_reversed.fasta&AAS65141&588+15^856+17^1112+10&588+15^856+17*)  [2.8](http://gemini.scripps.edu/cgi-bin/SeqCov?/scratch/yates/FlyBase_Drosophila_na_02-27-2005_con_reversed.fasta&Q9VGG9&588+15^856+17^1112+10&588+15^856+17*) | (AAS65141) CG31368-PB  (Q9VGG9) CG31368-PA |
| AAS65166  OSA_DROME | [21.2](../orb_02-updated_search.html#AAS65166) |  | [16.5](../orb_03-phos.html#AAS65166) |  | [21.8](http://gemini.scripps.edu/cgi-bin/SeqCov?/scratch/yates/FlyBase_Drosophila_na_02-27-2005_con_reversed.fasta&AAS65166&202+30^382+15^706+61^920+60^1082+28^1240+83^1345+29^1613+18^1637+10^1698+9^1815+13^1838+41^1938+14^2035+24^2069+10^2089+17^2142+34^2224+19^2251+42&382+15^746+21^961+19^1082+28^1240+65^1345+29^1613+18^1637+10^1698+9^1815+13^1838+41^2035+24^2069+10^2089+17^2142+34^2229+14^2251+42&382+15^746+21^961+19^1268+37^2036+9^2069+10^2229+14&746+21^961+19^1268+37^2069+10&748+19*)  [20.5](http://gemini.scripps.edu/cgi-bin/SeqCov?/scratch/yates/FlyBase_Drosophila_na_02-27-2005_con_reversed.fasta&OSA_DROME&202+30^382+15^706+61^920+60^1082+28^1400+83^1505+29^1773+18^1797! +10^1858+9^1975+13^1998+41^2098+14^2195+24^2229+10^2249+17^2302+34^2384+19^2411+42&382+15^746+21^961+19^1082+28^1400+65^1505+29^1773+18^1797+10^1858+9^1975+13^1998+41^2195+24^2229+10^2249+17^2302+34^2389+14^2411+42&382+15^746+21^961+19^1428+37^2196+9^2229+10^2389+14&746+21^961+19^1428+37^2229+10&748+19*) | (AAS65166) CG7467-PC  (Q8IN94) Trithorax group protein OSA (Eyelid protein) |
| AAS65273  DC13_DROME  DC12_DROME  DC11_DROME | [3.6](../orb_02-updated_search.html#AAS65273) |  | [3.6](../orb_03-phos.html#AAS65273) |  | [3.6](http://gemini.scripps.edu/cgi-bin/SeqCov?/scratch/yates/FlyBase_Drosophila_na_02-27-2005_con_reversed.fasta&AAS65273&288+14^320+11^893+40&288+14^320+11^893+40*)  [4.1](http://gemini.scripps.edu/cgi-bin/SeqCov?/scratch/yates/FlyBase_Drosophila_na_02-27-2005_con_reversed.fasta&DC13_DROME&288+14^320+11^874+40&288+14^320+11^874+40*)  [6.8](http://gemini.scripps.edu/cgi-bin/SeqCov?/scratch/yates/FlyBase_Drosophila_na_02-27-2005_con_reversed.fasta&DC12_DROME&288+14^320+11^874+40&288+14^320+11^874+40*)  [5.4](http://gemini.scripps.edu/cgi-bin/SeqCov?/scratch/yates/FlyBase_Drosophila_na_02-27-2005! _con_reversed.fasta&DC11_DROME&288+14^320+11^874+40&288+14^320+11^874+40*) | (AAS65273) CG2175-PD  (P18171) Defective chorion-1 protein, FC177 isoform precursor  (P18170) Defective chorion-1 protein, F106 isoform precursor  (P18169) Defective chorion-1 protein, FC125 isoform precursor |
| ACDM_DROME | [9.5](../orb_02-updated_search.html#ACDM_DROME) |  | [9.5](../orb_03-phos.html#ACDM_DROME) |  | [9.5](http://gemini.scripps.edu/cgi-bin/SeqCov?/scratch/yates/FlyBase_Drosophila_na_02-27-2005_con_reversed.fasta&ACDM_DROME&145+25^276+15&145+25^276+15&276+15&276+15*) | (Q9VSA3) Probable acyl-CoA dehydrogenase, medium-chain specific, mitochondrial precursor (EC 1.3.99.3) (MCAD) |
| BLM_DROME | [3.2](../orb_02-updated_search.html#BLM_DROME) |  | [3.2](../orb_03-phos.html#BLM_DROME) |  | [3.2](http://gemini.scripps.edu/cgi-bin/SeqCov?/scratch/yates/FlyBase_Drosophila_na_02-27-2005_con_reversed.fasta&BLM_DROME&725+12^846+16^1029+9^1289+10&725+12^846+16^1029+9^1289+10*) | (Q9VGI8) Bloom's syndrome protein homolog (EC 3.6.1.-) (Dmblm) (Mutagen-sensitive protein 309) (RecQ helicase homolog) |
| BRC1_DROME  Q9W572  BRC4_DROME | [7.2](../orb_02-updated_search.html#BRC1_DROME) |  | [7.2](../orb_03-phos.html#BRC1_DROME) |  | [7.2](http://gemini.scripps.edu/cgi-bin/SeqCov?/scratch/yates/FlyBase_Drosophila_na_02-27-2005_con_reversed.fasta&BRC1_DROME&112+28^170+24&112+28^170+24&170+24*)  [8.3](http://gemini.scripps.edu/cgi-bin/SeqCov?/scratch/yates/FlyBase_Drosophila_na_02-27-2005_con_reversed.fasta&Q9W572&112+28^170+24&112+28^170+24&170+24*)  [5.9](http://gemini.scripps.edu/cgi-bin/SeqCov?/scratch/yates/FlyBase_Drosophila_na_02-27-2005_con_reversed.fasta&BRC4_DROME&112+28^170+24&112+28^170+24&170+24*) | (Q01295) Broad-complex core-protein isoforms 1/2/3/4/5  (Q9W572) CG11491 protein  (Q24206) Broad-complex core-protein isoform 6 |
| BRM_DROME | [17.9](../orb_02-updated_search.html#BRM_DROME) |  | [17.9](../orb_03-phos.html#BRM_DROME) |  | [17.9](http://gemini.scripps.edu/cgi-bin/SeqCov?/scratch/yates/FlyBase_Drosophila_na_02-27-2005_con_reversed.fasta&BRM_DROME&171+24^335+44^399+13^513+13^591+20^821+23^912+14^954+32^1064+32^1295+9^1323+13^1343+13^1460+9^1581+35&171+24^335+44^399+13^513+13^591+20^821+23^912+14^954+32^1064+32^1295+9^1323+13^1343+13^1460+9^1581+35&171+13^513+13^826+18^912+14^1064+14^1591+25&171+13^513+13^826+18^912+14^1064+14^1591+25&1591+25*) | (P25439) Homeotic gene regulator (Brahma protein) |
| CH36_DROME | [15.7](../orb_02-updated_search.html#CH36_DROME) |  | [15.7](../orb_03-phos.html#CH36_DROME) |  | [15.7](http://gemini.scripps.edu/cgi-bin/SeqCov?/scratch/yates/FlyBase_Drosophila_na_02-27-2005_con_reversed.fasta&CH36_DROME&126+13^189+14^222+18&126+13^189+14^222+18&126+13*) | (P07182) Chorion protein S36 |
| CH38_DROME | [13.7](../orb_02-updated_search.html#CH38_DROME) |  | [13.7](../orb_03-phos.html#CH38_DROME) |  | [13.7](http://gemini.scripps.edu/cgi-bin/SeqCov?/scratch/yates/FlyBase_Drosophila_na_02-27-2005_con_reversed.fasta&CH38_DROME&117+14^279+28&117+14^279+28*) | (P07183) Chorion protein S38 |
| CRTC_DROME | [7.9](../orb_02-updated_search.html#CRTC_DROME) |  | [7.9](../orb_03-phos.html#CRTC_DROME) |  | [7.9](http://gemini.scripps.edu/cgi-bin/SeqCov?/scratch/yates/FlyBase_Drosophila_na_02-27-2005_con_reversed.fasta&CRTC_DROME&25+16^191+16&25+16^191+16*) | (P29413) Calreticulin precursor (CRP55) (Calregulin) (HACBP) |
| CYPH_DROME | [21.1](../orb_02-updated_search.html#CYPH_DROME) |  | [18.1](../orb_03-phos.html#CYPH_DROME) |  | [21.1](http://gemini.scripps.edu/cgi-bin/SeqCov?/scratch/yates/FlyBase_Drosophila_na_02-27-2005_con_reversed.fasta&CYPH_DROME&69+14^101+7^155+27&69+14^155+27&155+27&155+27*) | (P25007) Peptidyl-prolyl cis-trans isomerase (EC 5.2.1.8) (PPIase) (Rotamase) (Cyclophilin) (Cyclosporin A-binding protein) |
| DNJ1_DROME | [4.8](../orb_02-updated_search.html#DNJ1_DROME) |  | [4.8](../orb_03-phos.html#DNJ1_DROME) |  | [4.8](http://gemini.scripps.edu/cgi-bin/SeqCov?/scratch/yates/FlyBase_Drosophila_na_02-27-2005_con_reversed.fasta&DNJ1_DROME&274+16&274+16&274+16&274+16*) | (Q24133) DnaJ protein homolog 1 (DROJ1) |
| GBLP_DROME | [30.5](../orb_02-updated_search.html#GBLP_DROME) |  | [30.5](../orb_03-phos.html#GBLP_DROME) |  | [30.5](http://gemini.scripps.edu/cgi-bin/SeqCov?/scratch/yates/FlyBase_Drosophila_na_02-27-2005_con_reversed.fasta&GBLP_DROME&9+18^90+8^157+17^187+27^247+12^267+15&9+18^90+8^157+17^187+27^247+12^267+15&161+13^187+27&161+13^187+27*) | (O18640) Guanine nucleotide-binding protein beta subunit-like protein (Receptor of activated protein kinase C homolog) |
| GYS_DROME | [2.1](../orb_02-updated_search.html#GYS_DROME) |  | [2.1](../orb_03-phos.html#GYS_DROME) |  | [2.1](http://gemini.scripps.edu/cgi-bin/SeqCov?/scratch/yates/FlyBase_Drosophila_na_02-27-2005_con_reversed.fasta&GYS_DROME&318+15&318+15&318+15&318+15*) | (Q9VFC8) Putative glycogen [starch] synthase (EC 2.4.1.11) |
| HS26_DROME | [19.7](../orb_02-updated_search.html#HS26_DROME) |  | [19.7](../orb_03-phos.html#HS26_DROME) |  | [19.7](http://gemini.scripps.edu/cgi-bin/SeqCov?/scratch/yates/FlyBase_Drosophila_na_02-27-2005_con_reversed.fasta&HS26_DROME&18+13^74+13^175+15&18+13^74+13^175+15&18+13&18+13*) | (P02517) Heat shock protein 26 |
| HS27_DROME | [16.4](../orb_02-updated_search.html#HS27_DROME) |  | [16.4](../orb_03-phos.html#HS27_DROME) |  | [16.4](http://gemini.scripps.edu/cgi-bin/SeqCov?/scratch/yates/FlyBase_Drosophila_na_02-27-2005_con_reversed.fasta&HS27_DROME&142+20^176+15&142+20^176+15*) | (P02518) Heat shock protein 27 |
| IDHA_DROME | [15.4](../orb_02-updated_search.html#IDHA_DROME) |  | [9.3](../orb_03-phos.html#IDHA_DROME) |  | [15.4](http://gemini.scripps.edu/cgi-bin/SeqCov?/scratch/yates/FlyBase_Drosophila_na_02-27-2005_con_reversed.fasta&IDHA_DROME&69+20^94+15^163+23&69+20^94+15*) | (Q9VWH4) Probable isocitrate dehydrogenase [NAD] subunit alpha, mitochondrial precursor (EC 1.1.1.41) (Isocitric dehydrogenase) (NAD+-specific ICDH) |
| IF32_DROME | [4.9](../orb_02-updated_search.html#IF32_DROME) |  | [4.9](../orb_03-phos.html#IF32_DROME) |  | [4.9](http://gemini.scripps.edu/cgi-bin/SeqCov?/scratch/yates/FlyBase_Drosophila_na_02-27-2005_con_reversed.fasta&IF32_DROME&285+16&285+16&285+16&285+16*) | (O02195) Eukaryotic translation initiation factor 3 subunit 2 (eIF-3 beta) (eIF3i) (TRIP-1 homolog) |
| IF4A_DROME | [8.9](../orb_02-updated_search.html#IF4A_DROME) |  | [8.9](../orb_03-phos.html#IF4A_DROME) |  | [8.9](http://gemini.scripps.edu/cgi-bin/SeqCov?/scratch/yates/FlyBase_Drosophila_na_02-27-2005_con_reversed.fasta&IF4A_DROME&144+15^383+21&144+15^383+21*) | (Q02748) Eukaryotic initiation factor 4A (eIF4A) (eIF-4A) |
| IF4E_DROME | [34.4](../orb_02-updated_search.html#IF4E_DROME) |  | [37.8](../orb_03-phos.html#IF4E_DROME) |  | [37.8](http://gemini.scripps.edu/cgi-bin/SeqCov?/scratch/yates/FlyBase_Drosophila_na_02-27-2005_con_reversed.fasta&IF4E_DROME&20+24^49+26^131+9^141+12^157+7^205+20&20+24^49+26^141+12^157+7^205+20&20+24&20+24*) | (P48598) Eukaryotic translation initiation factor 4E (eIF4E) (eIF-4E) (mRNA cap-binding protein) (eIF-4F 25 kDa subunit) |
| **KC2A_DROME** | **[30.4](../orb_02-updated_search.html#KC2A_DROME)** |  | **[28.4](../orb_03-phos.html#KC2A_DROME)** |  | [**30.4**](http://gemini.scripps.edu/cgi-bin/SeqCov?/scratch/yates/FlyBase_Drosophila_na_02-27-2005_con_reversed.fasta&KC2A_DROME&105+22^140+13^170+19^227+15^245+13^281+20&105+15^140+13^170+19^227+15^245+13^281+20&105+15^170+19&170+19*) | **(P08181) Casein kinase II, alpha chain (EC 2.7.1.37) (CK II alpha subunit)** |
| NEP1_DROME | [16.3](../orb_02-updated_search.html#NEP1_DROME) |  | [16.3](../orb_03-phos.html#NEP1_DROME) |  | [16.3](http://gemini.scripps.edu/cgi-bin/SeqCov?/scratch/yates/FlyBase_Drosophila_na_02-27-2005_con_reversed.fasta&NEP1_DROME&57+18^133+11^161+12&57+18^133+11^161+12*) | (Q9W4J5) Probable ribosome biogenesis protein NEP1 |
| NONA_DROME  Q9VXH1  Q8IR16 | [23.1](../orb_02-updated_search.html#NONA_DROME) |  | [23.1](../orb_03-phos.html#NONA_DROME) |  | [23.1](http://gemini.scripps.edu/cgi-bin/SeqCov?/scratch/yates/FlyBase_Drosophila_na_02-27-2005_con_reversed.fasta&NONA_DROME&38+12^53+33^231+46^304+16^324+14^395+10^451+17^486+14&38+12^53+33^231+46^304+16^324+14^395+10^451+17^486+14&236+41&236+41&240+28*)  [23.1](http://gemini.scripps.edu/cgi-bin/SeqCov?/scratch/yates/FlyBase_Drosophila_na_02-27-2005_con_reversed.fasta&Q9VXH1&38+12^53+33^231+46^304+16^324+14^395+10^451+17^486+14&38+12^53+33^231+46^304+16^324+14^395+10^451+17^486+14&236+41&236+41&240+28*)  [21.8](http://gemini.scripps.edu/cgi-bin/SeqCov?/scratch/yates/FlyBase_Drosophila_na_02-27-2005_con_reversed.fasta&Q8IR16&38+12^53+3! 3^231+46^304+16^324+14^395+10^451+17^486+14&38+12^53+33^231+46^304+16^324+14^395+10^451+17^486+14&236+41&236+41&240+28*) | (Q04047) No-on-transient A protein  (Q9VXH1) CG4211 protein (RE58280p)  (Q8IR16) CG4211-PB |
| OTU_DROME | [7.2](../orb_02-updated_search.html#OTU_DROME) |  | [7.2](../orb_03-phos.html#OTU_DROME) |  | [7.2](http://gemini.scripps.edu/cgi-bin/SeqCov?/scratch/yates/FlyBase_Drosophila_na_02-27-2005_con_reversed.fasta&OTU_DROME&381+22^430+12^642+27&381+22^430+12^642+27*) | (P10383) Ovarian tumor locus protein |
| PABP_DROME | [20.9](../orb_02-updated_search.html#PABP_DROME) |  | [20.9](../orb_03-phos.html#PABP_DROME) |  | [20.9](http://gemini.scripps.edu/cgi-bin/SeqCov?/scratch/yates/FlyBase_Drosophila_na_02-27-2005_con_reversed.fasta&PABP_DROME&42+28^278+13^424+36^467+20^529+20^574+15&42+28^278+13^424+36^467+20^529+20^574+15&467+20^529+20&467+20^529+20*) | (P21187) Polyadenylate-binding protein (Poly(A)-binding protein) (PABP) |
| PIT_DROME | [8.4](../orb_02-updated_search.html#PIT_DROME) |  | [8.4](../orb_03-phos.html#PIT_DROME) |  | [8.4](http://gemini.scripps.edu/cgi-bin/SeqCov?/scratch/yates/FlyBase_Drosophila_na_02-27-2005_con_reversed.fasta&PIT_DROME&164+17^448+19^527+21&164+17^448+19^527+21*) | (Q9VD51) Probable ATP-dependent helicase pitchoune |
| PP11_DROME  PP12_DROME | [10.7](../orb_02-updated_search.html#PP11_DROME) |  | [10.7](../orb_03-phos.html#PP11_DROME) |  | [10.7](http://gemini.scripps.edu/cgi-bin/SeqCov?/scratch/yates/FlyBase_Drosophila_na_02-27-2005_con_reversed.fasta&PP11_DROME&42+17^149+18&42+17^149+18*)  [11.6](http://gemini.scripps.edu/cgi-bin/SeqCov?/scratch/yates/FlyBase_Drosophila_na_02-27-2005_con_reversed.fasta&PP12_DROME&42+17^149+18&42+17^149+18*) | (P48461) Serine/threonine protein phosphatase alpha-1 isoform (EC 3.1.3.16)  (P12982) Serine/threonine protein phosphatase alpha-2 isoform (EC 3.1.3.16) |
| PPOL_DROME  Q7PLT6 | [24.1](../orb_02-updated_search.html#PPOL_DROME) |  | [22.9](../orb_03-phos.html#PPOL_DROME) |  | [24.1](http://gemini.scripps.edu/cgi-bin/SeqCov?/scratch/yates/FlyBase_Drosophila_na_02-27-2005_con_reversed.fasta&PPOL_DROME&58+17^80+19^150+42^298+14^332+10^369+12^467+15^605+8^622+13^718+27^762+19^789+13^848+13^947+18&58+17^80+19^150+42^298+14^332+10^467+15^605+8^622+13^718+27^762+19^789+13^848+13^947+18&58+17^150+17^848+13&58+17^150+17^848+13&852+9*)  [24.1](http://gemini.scripps.edu/cgi-bin/SeqCov?/scratch/yates/FlyBase_Drosophila_na_02-27-2005_con_reversed.fasta&Q7PLT6&58+17^80+19^150+42^298+14^332+10^369+12^467+15^605+8^622+13^718+27^762+19^789+13^848+13^947+18&58+17^80+19^150+42^298+14^332+10^467+15^605+8^622+13^718+27^762+19^789+13^848+13^947+18&58+17^150+17^848+1! 3&58+17^150+17^848+13&852+9*) | (P35875) Poly [ADP-ribose] polymerase (EC 2.4.2.30) (PARP) (ADPRT) (NAD(+) ADP-ribosyltransferase) (Poly[ADP-ribose] synthetase)  (Q7PLT6) CG40411-PC |
| Q24009  Q8IP26 | [7.0](../orb_02-updated_search.html#Q24009) |  | [7.0](../orb_03-phos.html#Q24009) |  | [7.0](http://gemini.scripps.edu/cgi-bin/SeqCov?/scratch/yates/FlyBase_Drosophila_na_02-27-2005_con_reversed.fasta&Q24009&557+32^858+31&557+32^858+31*)  [8.0](http://gemini.scripps.edu/cgi-bin/SeqCov?/scratch/yates/FlyBase_Drosophila_na_02-27-2005_con_reversed.fasta&Q8IP26&437+32^738+31&437+32^738+31*) | (Q24009) Bicaudal C protein  (Q8IP26) CG4824-PD |
| Q24090 | [46.8](../orb_02-updated_search.html#Q24090) |  | [46.5](../orb_03-phos.html#Q24090) |  | [46.8](http://gemini.scripps.edu/cgi-bin/SeqCov?/scratch/yates/FlyBase_Drosophila_na_02-27-2005_con_reversed.fasta&Q24090&59+33^112+26^147+21^193+54^310+39&59+33^113+25^147+21^193+54^310+39&59+19^113+25&59+19^113+25*) | (Q24090) SNR1 protein (GH08712p) |
| Q26459  Q9VRM5  Q8IQ63 | [18.1](../orb_02-updated_search.html#Q26459) |  | [17.3](../orb_03-phos.html#Q26459) |  | [18.1](http://gemini.scripps.edu/cgi-bin/SeqCov?/scratch/yates/FlyBase_Drosophila_na_02-27-2005_con_reversed.fasta&Q26459&72+27^103+25^161+16^202+15^277+13^349+14&72+27^103+25^161+16^202+15^277+13^354+9&354+9&354+9&354+9*)  [17.8](http://gemini.scripps.edu/cgi-bin/SeqCov?/scratch/yates/FlyBase_Drosophila_na_02-27-2005_con_reversed.fasta&Q9VRM5&81+27^112+25^170+16^211+15^286+13^358+14&81+27^112+25^170+16^211+15^286+13^363+9&363+9&363+9&363+9*)  [17.6](http://gemini.scripps.edu/cgi-bin/SeqCov?/scratch/yates/FlyBase_Drosophila_na_02-27-2005_con_reversed.fasta&Q8IQ63&89+27^120+25^178+16^219+15^294+13^366+14&89+27^120+25^178+16^219+15^294+13^371+9&371+9! &371+9&371+9*) | (Q26459) MSR-110 protein (LD44960p) (CG10596-PB)  (Q9VRM5) MSR-110 protein  (Q8IQ63) CG10596-PC |
| Q86B94  Q9VGF0  Q9VGE9  Q86B97  Q86B96  Q86B95 | [3.8](../orb_02-updated_search.html#Q86B94) |  | [3.8](../orb_03-phos.html#Q86B94) |  | [3.8](http://gemini.scripps.edu/cgi-bin/SeqCov?/scratch/yates/FlyBase_Drosophila_na_02-27-2005_con_reversed.fasta&Q86B94&322+15^434+19&322+15^434+19*)  [3.9](http://gemini.scripps.edu/cgi-bin/SeqCov?/scratch/yates/FlyBase_Drosophila_na_02-27-2005_con_reversed.fasta&Q9VGF0&298+15^410+19&298+15^410+19*)  [4.2](http://gemini.scripps.edu/cgi-bin/SeqCov?/scratch/yates/FlyBase_Drosophila_na_02-27-2005_con_reversed.fasta&Q9VGE9&233+15^345+19&233+15^345+19*)  [4.2](http://gemini.scripps.edu/cgi-bin/SeqCov?/scratch/yates/FlyBase_Drosophila_na_02-27-2005_con_reversed.fasta&Q86B97&232+15^344+19&232+15^34! 4+19*)  [4.1](http://gemini.scripps.edu/cgi-bin/SeqCov?/scratch/yates/FlyBase_Drosophila_na_02-27-2005_con_reversed.fasta&Q86B96&256+15^368+19&256+15^368+19*)  [4.0](http://gemini.scripps.edu/cgi-bin/SeqCov?/scratch/yates/FlyBase_Drosophila_na_02-27-2005_con_reversed.fasta&Q86B95&257+15^369+19&257+15^369+19*) | (Q86B94) CG3359-PJ  (Q9VGF0) MFAS protein  (Q9VGE9) MFAS protein (GH11519p)  (Q86B97) CG3359-PH (CG3359-PN)  (Q86B96) CG3359-PE (CG3359-PO)  (Q86B95) CG3359-PF (CG3359-PG) |
| Q8I0G5  Q9V9W9 | [6.5](../orb_02-updated_search.html#Q8I0G5) |  | [6.5](../orb_03-phos.html#Q8I0G5) |  | [6.5](http://gemini.scripps.edu/cgi-bin/SeqCov?/scratch/yates/FlyBase_Drosophila_na_02-27-2005_con_reversed.fasta&Q8I0G5&625+15^659+13^816+29&625+15^659+13^816+29*)  [6.5](http://gemini.scripps.edu/cgi-bin/SeqCov?/scratch/yates/FlyBase_Drosophila_na_02-27-2005_con_reversed.fasta&Q9V9W9&621+15^655+13^812+29&621+15^655+13^812+29*) | (Q8I0G5) CG1528-PB (RE37840p)  (Q9V9W9) GAMMACOP protein |
| Q8IM93 | [10.2](../orb_02-updated_search.html#Q8IM93) |  | [7.1](../orb_03-phos.html#Q8IM93) |  | [13.1](http://gemini.scripps.edu/cgi-bin/SeqCov?/scratch/yates/FlyBase_Drosophila_na_02-27-2005_con_reversed.fasta&Q8IM93&72+13^213+9^301+37&301+19&301+19*) | (Q8IM93) CG32017-PA |
| Q8IMI7  Q9VA91 | [16.8](../orb_02-updated_search.html#Q8IMI7) |  | [16.8](../orb_03-phos.html#Q8IMI7) |  | [16.8](http://gemini.scripps.edu/cgi-bin/SeqCov?/scratch/yates/FlyBase_Drosophila_na_02-27-2005_con_reversed.fasta&Q8IMI7&21+20^119+22&21+20^119+22*)  [21.6](http://gemini.scripps.edu/cgi-bin/SeqCov?/scratch/yates/FlyBase_Drosophila_na_02-27-2005_con_reversed.fasta&Q9VA91&21+20^119+22&21+20^119+22*) | (Q8IMI7) CG1883-PB  (Q9VA91) CG1883 protein (RE18653p) |
| Q8INE0  Q9VF64  Q8SWR8 | [5.8](../orb_02-updated_search.html#Q8INE0) |  | [5.8](../orb_03-phos.html#Q8INE0) |  | [5.8](http://gemini.scripps.edu/cgi-bin/SeqCov?/scratch/yates/FlyBase_Drosophila_na_02-27-2005_con_reversed.fasta&Q8INE0&57+15^265+19^358+25&57+15^265+19^358+25*)  [6.6](http://gemini.scripps.edu/cgi-bin/SeqCov?/scratch/yates/FlyBase_Drosophila_na_02-27-2005_con_reversed.fasta&Q9VF64&57+15^265+19^358+25&57+15^265+19^358+25*)  [5.4](http://gemini.scripps.edu/cgi-bin/SeqCov?/scratch/yates/FlyBase_Drosophila_na_02-27-2005_con_reversed.fasta&Q8SWR8&118+15^326+19^419+25&118+15^326+19^419+25*) | (Q8INE0) CG5166-PC  (Q9VF64) CG5166-PA  (Q8SWR8) GH13857p (CG5166-PB) |
| Q8INH9  Q9VG05 | [4.5](../orb_02-updated_search.html#Q8INH9) |  | [3.7](../orb_03-phos.html#Q8INH9) |  | [4.5](http://gemini.scripps.edu/cgi-bin/SeqCov?/scratch/yates/FlyBase_Drosophila_na_02-27-2005_con_reversed.fasta&Q8INH9&561+14^610+20^1411+19^1772+17^2008+18^2111+18&561+14^610+20^1411+19^1772+17^2008+18&2008+18&2008+18*)  [4.3](http://gemini.scripps.edu/cgi-bin/SeqCov?/scratch/yates/FlyBase_Drosophila_na_02-27-2005_con_reversed.fasta&Q9VG05&561+14^610+20^1411+19^1772+17^2008+18^2111+18&561+14^610+20^1411+19^1772+17^2008+18&2008+18&2008+18*) | (Q8INH9) CG7518-PB  (Q9VG05) CG7518 protein |
| Q8IPB9  Q9VKX1 | [5.2](../orb_02-updated_search.html#Q8IPB9) |  | [5.2](../orb_03-phos.html#Q8IPB9) |  | [5.2](http://gemini.scripps.edu/cgi-bin/SeqCov?/scratch/yates/FlyBase_Drosophila_na_02-27-2005_con_reversed.fasta&Q8IPB9&621+27^688+27&621+27^688+27*)  [5.1](http://gemini.scripps.edu/cgi-bin/SeqCov?/scratch/yates/FlyBase_Drosophila_na_02-27-2005_con_reversed.fasta&Q9VKX1&625+27^692+27&625+27^692+27*) | (Q8IPB9) CG31716-PG  (Q9VKX1) CG31716 protein (RE04975p) |
| Q8IQG4  Q9VTP4 | [10.0](../orb_02-updated_search.html#Q8IQG4) |  | [10.0](../orb_03-phos.html#Q8IQG4) |  | [10.0](http://gemini.scripps.edu/cgi-bin/SeqCov?/scratch/yates/FlyBase_Drosophila_na_02-27-2005_con_reversed.fasta&Q8IQG4&57+14&57+14&57+14&57+14*)  [6.5](http://gemini.scripps.edu/cgi-bin/SeqCov?/scratch/yates/FlyBase_Drosophila_na_02-27-2005_con_reversed.fasta&Q9VTP4&134+14&134+14&134+14&134+14*) | (Q8IQG4) CG7283-PC  (Q9VTP4) CG7283 protein |
| Q8IQV9  Q9VWB8 | [0.9](../orb_02-updated_search.html#Q8IQV9) |  | [0.9](../orb_03-phos.html#Q8IQV9) |  | [0.9](http://gemini.scripps.edu/cgi-bin/SeqCov?/scratch/yates/FlyBase_Drosophila_na_02-27-2005_con_reversed.fasta&Q8IQV9&1249+18&1249+18&1249+18&1249+18*)  [0.9](http://gemini.scripps.edu/cgi-bin/SeqCov?/scratch/yates/FlyBase_Drosophila_na_02-27-2005_con_reversed.fasta&Q9VWB8&1226+18&1226+18&1226+18&1226+18*) | (Q8IQV9) CG11943-PB  (Q9VWB8) CG11943 protein |
| Q8IRM8  Q9W392 | [6.4](../orb_02-updated_search.html#Q8IRM8) |  | [6.4](../orb_03-phos.html#Q8IRM8) |  | [6.4](http://gemini.scripps.edu/cgi-bin/SeqCov?/scratch/yates/FlyBase_Drosophila_na_02-27-2005_con_reversed.fasta&Q8IRM8&119+34&119+34&130+23&130+23*)  [6.4](http://gemini.scripps.edu/cgi-bin/SeqCov?/scratch/yates/FlyBase_Drosophila_na_02-27-2005_con_reversed.fasta&Q9W392&121+34&121+34&132+23&132+23*) | (Q8IRM8) CG7033-PB  (Q9W392) CG7033 protein (SD02216p) |
| Q8MKM0  Q9V4M6  Q8MKM1 | [4.4](../orb_02-updated_search.html#Q8MKM0) |  | [4.4](../orb_03-phos.html#Q8MKM0) |  | [4.4](http://gemini.scripps.edu/cgi-bin/SeqCov?/scratch/yates/FlyBase_Drosophila_na_02-27-2005_con_reversed.fasta&Q8MKM0&65+27^330+15^471+11&65+27^330+15^471+11*)  [3.0](http://gemini.scripps.edu/cgi-bin/SeqCov?/scratch/yates/FlyBase_Drosophila_na_02-27-2005_con_reversed.fasta&Q9V4M6&65+27^330+15^471+11&65+27^330+15^471+11*)  [2.9](http://gemini.scripps.edu/cgi-bin/SeqCov?/scratch/yates/FlyBase_Drosophila_na_02-27-2005_con_reversed.fasta&Q8MKM1&65+27^330+15^471+11&65+27^330+15^471+11*) | (Q8MKM0) CG2146-PB  (Q9V4M6) Didum protein  (Q8MKM1) CG2146-PC |
| Q8MKX9  Q9V5L1 | [19.6](../orb_02-updated_search.html#Q8MKX9) |  | [19.6](../orb_03-phos.html#Q8MKX9) |  | [19.6](http://gemini.scripps.edu/cgi-bin/SeqCov?/scratch/yates/FlyBase_Drosophila_na_02-27-2005_con_reversed.fasta&Q8MKX9&203+24^248+48^355+20^561+25&203+24^248+48^355+20^561+25&561+16&561+16&561+16*)  [12.2](http://gemini.scripps.edu/cgi-bin/SeqCov?/scratch/yates/FlyBase_Drosophila_na_02-27-2005_con_reversed.fasta&Q9V5L1&565+24^610+48^717+20^923+25&565+24^610+48^717+20^923+25&923+16&923+16&923+16*) | (Q8MKX9) CG11763-PC  (Q9V5L1) CG11763 protein |
| Q8ML06  Q9V679 | [24.0](../orb_02-updated_search.html#Q8ML06) |  | [24.0](../orb_03-phos.html#Q8ML06) |  | [24.0](http://gemini.scripps.edu/cgi-bin/SeqCov?/scratch/yates/FlyBase_Drosophila_na_02-27-2005_con_reversed.fasta&Q8ML06&86+8^104+29&86+8^104+29&104+11&104+11*)  [23.9](http://gemini.scripps.edu/cgi-bin/SeqCov?/scratch/yates/FlyBase_Drosophila_na_02-27-2005_con_reversed.fasta&Q9V679&87+8^105+29&87+8^105+29&105+11&105+11*) | (Q8ML06) CG8857-PB  (Q9V679) CG8857 protein |
| Q8ML98  Q8ML99 | [14.0](../orb_02-updated_search.html#Q8ML98) |  | [14.0](../orb_03-phos.html#Q8ML98) |  | [14.0](http://gemini.scripps.edu/cgi-bin/SeqCov?/scratch/yates/FlyBase_Drosophila_na_02-27-2005_con_reversed.fasta&Q8ML98&142+21^399+32^575+19^595+13&142+21^399+32^575+19^595+13*)  [11.1](http://gemini.scripps.edu/cgi-bin/SeqCov?/scratch/yates/FlyBase_Drosophila_na_02-27-2005_con_reversed.fasta&Q8ML99&299+21^556+32^732+19^752+13&299+21^556+32^732+19^752+13*) | (**Q8ML98) CG8174-PC SPRK**  **(Q8ML99) CG8174-PA** **SPRK** |
| Q8MMB3  Q9V8R3 | [16.8](../orb_02-updated_search.html#Q8MMB3) |  | [16.4](../orb_03-phos.html#Q8MMB3) |  | [16.8](http://gemini.scripps.edu/cgi-bin/SeqCov?/scratch/yates/FlyBase_Drosophila_na_02-27-2005_con_reversed.fasta&Q8MMB3&198+14^215+12^248+12^293+35^655+26^729+40&198+14^215+12^248+12^296+32^655+26^729+40&296+32^729+37*)  [20.3](http://gemini.scripps.edu/cgi-bin/SeqCov?/scratch/yates/FlyBase_Drosophila_na_02-27-2005_con_reversed.fasta&Q9V8R3&53+14^70+12^103+12^148+35^510+26^584+40&53+14^70+12^103+12^151+32^510+26^584+40&151+32^584+37*) | (Q8MMB3) CG15112-PB  (Q9V8R3) CG15112 protein |
| Q8MRH7  Q9VEG6 | [5.7](../orb_02-updated_search.html#Q8MRH7) |  | [5.7](../orb_03-phos.html#Q8MRH7) |  | [5.7](http://gemini.scripps.edu/cgi-bin/SeqCov?/scratch/yates/FlyBase_Drosophila_na_02-27-2005_con_reversed.fasta&Q8MRH7&158+16^536+30&158+16^536+30*)  [5.5](http://gemini.scripps.edu/cgi-bin/SeqCov?/scratch/yates/FlyBase_Drosophila_na_02-27-2005_con_reversed.fasta&Q9VEG6&158+16^558+30&158+16^558+30*) | (Q8MRH7) LD43174p (CG7660-PB)  (Q9VEG6) CG7660 protein |
| Q95WY3 | [8.5](../orb_02-updated_search.html#Q95WY3) |  | [8.5](../orb_03-phos.html#Q95WY3) |  | [8.5](http://gemini.scripps.edu/cgi-bin/SeqCov?/scratch/yates/FlyBase_Drosophila_na_02-27-2005_con_reversed.fasta&Q95WY3&223+12^309+13^383+17&223+12^309+13^383+17*) | (Q95WY3) Nucleolar KKE/D repeat protein (CG13849-PA) |
| Q960X9  Q9VW20 | [8.7](../orb_02-updated_search.html#Q960X9) |  | [8.7](../orb_03-phos.html#Q960X9) |  | [8.7](http://gemini.scripps.edu/cgi-bin/SeqCov?/scratch/yates/FlyBase_Drosophila_na_02-27-2005_con_reversed.fasta&Q960X9&173+25^367+12^447+35&173+25^367+12^447+35*)  [7.2](http://gemini.scripps.edu/cgi-bin/SeqCov?/scratch/yates/FlyBase_Drosophila_na_02-27-2005_con_reversed.fasta&Q9VW20&347+25^541+12^621+35&347+25^541+12^621+35*) | (Q960X9) LD30525p (EC 3.4.21.-) (Mitochondrial Lon protease homolog) (CG8798-PB)  (Q9VW20) CG8798 protein (EC 3.4.21.-) (Mitochondrial Lon protease homolog) |
| Q9NFU0  Q9TVY4 | [45.0](../orb_02-updated_search.html#Q9NFU0) |  | [40.5](../orb_03-phos.html#Q9NFU0) |  | [45.0](http://gemini.scripps.edu/cgi-bin/SeqCov?/scratch/yates/FlyBase_Drosophila_na_02-27-2005_con_reversed.fasta&Q9NFU0&18+30^73+15^99+15^127+19^155+38^220+42^267+11^280+14^337+40^385+17^406+10^451+9^537+12^601+16^657+20&18+30^73+15^99+15^127+19^155+38^220+42^267+11^280+14^337+40^406+10^451+9^601+14^657+20&73+15^127+19^166+10^220+29^280+14^337+37^406+10^601+14&73+15^127+19^166+10^220+29^280+14^337+37^406+10&77+11^132+14^166+10^230+19^337+37*)  [45.2](http://gemini.scripps.edu/cgi-bin/SeqCov?/scratch/yates/FlyBase_Drosophila_na_02-27-2005_con_reversed.fasta&Q9TVY4&18+30^73+15^99+15^127+19^155+38^220+42^267+11^280+14^334+40^382+17^403+10^448+9^534+12^598+16^654+20&18+30^73+15^99+15^12! 7+19^155+38^220+42^267+11^280+14^334+40^403+10^448+9^598+14^654+20&73+15^127+19^166+10^220+29^280+14^334+37^403+10^598+14&73+15^127+19^166+10^220+29^280+14^334+37^403+10&77+11^132+14^166+10^230+19^334+37*) | (Q9NFU0) Fragile X related protein (CG6203-PA)  (Q9TVY4) Fragile X related (CG6203-PC) |
| Q9V378 | [6.0](../orb_02-updated_search.html#Q9V378) |  | [3.8](../orb_03-phos.html#Q9V378) |  | [6.0](http://gemini.scripps.edu/cgi-bin/SeqCov?/scratch/yates/FlyBase_Drosophila_na_02-27-2005_con_reversed.fasta&Q9V378&419+11^474+17^543+19&419+11^543+19*) | (Q9V378) BCDNA:GM05306 protein (LD15481p) |
| Q9V3G1 | [3.9](../orb_02-updated_search.html#Q9V3G1) |  | [3.9](../orb_03-phos.html#Q9V3G1) |  | [3.9](http://gemini.scripps.edu/cgi-bin/SeqCov?/scratch/yates/FlyBase_Drosophila_na_02-27-2005_con_reversed.fasta&Q9V3G1&55+10&55+10&55+10&55+10*) | (Q9V3G1) CG1263 protein (RE37829p) |
| Q9V3J4 | [7.6](../orb_02-updated_search.html#Q9V3J4) |  | [7.6](../orb_03-phos.html#Q9V3J4) |  | [7.6](http://gemini.scripps.edu/cgi-bin/SeqCov?/scratch/yates/FlyBase_Drosophila_na_02-27-2005_con_reversed.fasta&Q9V3J4&146+27&146+27&151+22&151+22*) | (Q9V3J4) BCDNA:LD03471 protein |
| Q9V3P6 | [7.3](../orb_02-updated_search.html#Q9V3P6) |  | [4.8](../orb_03-phos.html#Q9V3P6) |  | [8.4](http://gemini.scripps.edu/cgi-bin/SeqCov?/scratch/yates/FlyBase_Drosophila_na_02-27-2005_con_reversed.fasta&Q9V3P6&60+12^445+37^971+37&971+37*) | (Q9V3P6) RPN2 protein |
| Q9V3U2 | [16.2](../orb_02-updated_search.html#Q9V3U2) |  | [16.2](../orb_03-phos.html#Q9V3U2) |  | [16.2](http://gemini.scripps.edu/cgi-bin/SeqCov?/scratch/yates/FlyBase_Drosophila_na_02-27-2005_con_reversed.fasta&Q9V3U2&129+9^144+17&129+9^144+17&144+17*) | (Q9V3U2) CG5258 protein |
| Q9V3W7 | [15.3](../orb_02-updated_search.html#Q9V3W7) |  | [15.3](../orb_03-phos.html#Q9V3W7) |  | [15.3](http://gemini.scripps.edu/cgi-bin/SeqCov?/scratch/yates/FlyBase_Drosophila_na_02-27-2005_con_reversed.fasta&Q9V3W7&43+13^117+16^149+10&43+13^117+16^149+10*) | (Q9V3W7) CG6987 protein (SR family splicing factor) (LD40489P) |
| Q9V4F9 | [1.9](../orb_02-updated_search.html#Q9V4F9) |  | [1.9](../orb_03-phos.html#Q9V4F9) |  | [1.9](http://gemini.scripps.edu/cgi-bin/SeqCov?/scratch/yates/FlyBase_Drosophila_na_02-27-2005_con_reversed.fasta&Q9V4F9&242+19^1233+9&242+19^1233+9*) | (Q9V4F9) CG1793 protein |
| Q9V4Z5 | [5.0](../orb_02-updated_search.html#Q9V4Z5) |  | [5.0](../orb_03-phos.html#Q9V4Z5) |  | [5.0](http://gemini.scripps.edu/cgi-bin/SeqCov?/scratch/yates/FlyBase_Drosophila_na_02-27-2005_con_reversed.fasta&Q9V4Z5&428+11^462+20&428+11^462+20*) | (Q9V4Z5) CG30349 protein |
| Q9V5G1 | [0.8](../orb_02-updated_search.html#Q9V5G1) |  | [0.8](../orb_03-phos.html#Q9V5G1) |  | [0.8](http://gemini.scripps.edu/cgi-bin/SeqCov?/scratch/yates/FlyBase_Drosophila_na_02-27-2005_con_reversed.fasta&Q9V5G1&94+9&94+9&94+9&94+9*) | (Q9V5G1) CG1371 protein |
| Q9V6U5 | [8.8](../orb_02-updated_search.html#Q9V6U5) |  | [8.8](../orb_03-phos.html#Q9V6U5) |  | [8.8](http://gemini.scripps.edu/cgi-bin/SeqCov?/scratch/yates/FlyBase_Drosophila_na_02-27-2005_con_reversed.fasta&Q9V6U5&217+13^234+13&217+13^234+13*) | (Q9V6U5) CG18645 protein (LD24265p) |
| Q9V814 | [30.1](../orb_02-updated_search.html#Q9V814) |  | [35.8](../orb_03-phos.html#Q9V814) |  | [35.8](http://gemini.scripps.edu/cgi-bin/SeqCov?/scratch/yates/FlyBase_Drosophila_na_02-27-2005_con_reversed.fasta&Q9V814&3+24^37+22^73+12^95+12^141+18^170+40^283+24&37+22^73+12^95+12^141+18^170+40^283+24&37+22^283+24*) | (Q9V814) CG6546 protein (LD29458p) |
| Q9V8D6 | [57.2](../orb_02-updated_search.html#Q9V8D6) |  | [57.2](../orb_03-phos.html#Q9V8D6) |  | [57.2](http://gemini.scripps.edu/cgi-bin/SeqCov?/scratch/yates/FlyBase_Drosophila_na_02-27-2005_con_reversed.fasta&Q9V8D6&21+36^59+44^105+26^141+8^167+13^186+14^202+32^237+14^255+11^267+11^285+125^424+31^526+19^677+17^716+21^751+16&21+36^59+44^105+26^141+8^167+13^186+14^202+32^237+14^255+11^267+11^285+125^424+31^526+19^677+17^716+21^751+16&105+11^141+8^186+14^202+32^267+11^334+28^391+19^717+20&105+11^141+8^186+14^202+32^267+11^334+28^391+19^717+20&209+13^391+19^717+20*) | (Q9V8D6) CG5726 protein (RE28271p) |
| Q9V8K6 | [8.3](../orb_02-updated_search.html#Q9V8K6) |  | [8.3](../orb_03-phos.html#Q9V8K6) |  | [8.3](http://gemini.scripps.edu/cgi-bin/SeqCov?/scratch/yates/FlyBase_Drosophila_na_02-27-2005_con_reversed.fasta&Q9V8K6&38+19^300+13^353+14^383+19^569+27^735+13&38+19^300+13^353+14^383+19^569+27^735+13*) | (Q9V8K6) CG30122 protein |
| Q9VAX7 | [16.1](../orb_02-updated_search.html#Q9VAX7) |  | [16.1](../orb_03-phos.html#Q9VAX7) |  | [16.1](http://gemini.scripps.edu/cgi-bin/SeqCov?/scratch/yates/FlyBase_Drosophila_na_02-27-2005_con_reversed.fasta&Q9VAX7&78+26^307+10^318+24^402+14^446+10&78+26^307+10^318+24^402+14^446+10*) | (Q9VAX7) CG4869 protein |
| Q9VBN5 | [15.6](../orb_02-updated_search.html#Q9VBN5) |  | [15.6](../orb_03-phos.html#Q9VBN5) |  | [15.6](http://gemini.scripps.edu/cgi-bin/SeqCov?/scratch/yates/FlyBase_Drosophila_na_02-27-2005_con_reversed.fasta&Q9VBN5&74+21&74+21*) | (Q9VBN5) 60S ribosomal protein L27 (AT27980p) |
| Q9VBU9 | [28.6](../orb_02-updated_search.html#Q9VBU9) |  | [28.6](../orb_03-phos.html#Q9VBU9) |  | [28.6](http://gemini.scripps.edu/cgi-bin/SeqCov?/scratch/yates/FlyBase_Drosophila_na_02-27-2005_con_reversed.fasta&Q9VBU9&6+11^24+13&6+11^24+13*) | (Q9VBU9) CG10423 protein (Ribosomal protein S27) (LD37859p) |
| Q9VC28 | [17.8](../orb_02-updated_search.html#Q9VC28) |  | [17.8](../orb_03-phos.html#Q9VC28) |  | [17.8](http://gemini.scripps.edu/cgi-bin/SeqCov?/scratch/yates/FlyBase_Drosophila_na_02-27-2005_con_reversed.fasta&Q9VC28&26+13^97+19&26+13^97+19&97+19&97+19*) | (Q9VC28) CG7006 protein (GM12126p) |
| Q9VCA8 | [8.8](../orb_02-updated_search.html#Q9VCA8) |  | [8.0](../orb_03-phos.html#Q9VCA8) |  | [8.8](http://gemini.scripps.edu/cgi-bin/SeqCov?/scratch/yates/FlyBase_Drosophila_na_02-27-2005_con_reversed.fasta&Q9VCA8&253+21^703+17^808+20^1388+23^1558+13^1788+62^1942+16^2345+16^2410+21^2823+19^2919+25^3294+17^3743+30^3877+27^3954+26&253+21^703+17^808+20^1388+23^1558+13^1822+28^1942+16^2345+16^2410+21^2823+19^2919+25^3294+17^3743+30^3877+27^3954+26&253+21^1388+23^2345+16&253+21^1388+23^2345+16*) | (Q9VCA8) CG33106-PA (CG33106-PB) |
| Q9VCN7 | [30.9](../orb_02-updated_search.html#Q9VCN7) |  | [29.3](../orb_03-phos.html#Q9VCN7) |  | [32.7](http://gemini.scripps.edu/cgi-bin/SeqCov?/scratch/yates/FlyBase_Drosophila_na_02-27-2005_con_reversed.fasta&Q9VCN7&2+32^234+18^256+22^342+31^389+16^549+19^578+26^610+8^632+17^663+16^699+26^797+13^820+22^868+33&2+32^234+18^256+22^549+19^578+26^610+8^632+17^663+16^699+26^797+13^820+22^868+33&21+13^549+19^578+26^663+16&549+19^580+24^663+16&580+24^668+11*) | (Q9VCN7) ORB protein |
| Q9VDE3 | [7.3](../orb_02-updated_search.html#Q9VDE3) |  | [7.3](../orb_03-phos.html#Q9VDE3) |  | [7.3](http://gemini.scripps.edu/cgi-bin/SeqCov?/scratch/yates/FlyBase_Drosophila_na_02-27-2005_con_reversed.fasta&Q9VDE3&87+28^154+9&87+28^154+9*) | (Q9VDE3) SLMB protein (SLIMB) (LD08669p) |
| Q9VDR1 | [12.9](../orb_02-updated_search.html#Q9VDR1) |  | [12.9](../orb_03-phos.html#Q9VDR1) |  | [12.9](http://gemini.scripps.edu/cgi-bin/SeqCov?/scratch/yates/FlyBase_Drosophila_na_02-27-2005_con_reversed.fasta&Q9VDR1&67+21^137+20^173+10^543+14^579+12^594+17^645+17&67+21^137+20^173+10^543+14^579+12^594+17^645+17&579+12^594+17&579+12^594+17*) | (Q9VDR1) CG12254 protein (LD07688p) |
| Q9VEC1 | [23.5](../orb_02-updated_search.html#Q9VEC1) |  | [23.5](../orb_03-phos.html#Q9VEC1) |  | [23.5](http://gemini.scripps.edu/cgi-bin/SeqCov?/scratch/yates/FlyBase_Drosophila_na_02-27-2005_con_reversed.fasta&Q9VEC1&30+11^61+26^111+21^311+19^416+18^514+56&30+11^61+26^111+21^311+19^416+18^514+56&311+19^534+17&311+19^534+17*) | (Q9VEC1) CG7957 protein (Mediator transcriptional cofactor TRAP80) (Thyroid hormone receptor-associated protein TRAP80) (SD10038p) |
| Q9VFV9 | [13.9](../orb_02-updated_search.html#Q9VFV9) |  | [13.9](../orb_03-phos.html#Q9VFV9) |  | [13.9](http://gemini.scripps.edu/cgi-bin/SeqCov?/scratch/yates/FlyBase_Drosophila_na_02-27-2005_con_reversed.fasta&Q9VFV9&47+14^168+17^228+25&47+14^168+17^228+25&168+17&168+17*) | (Q9VFV9) CG8863 protein (GM13664p) |
| Q9VHI5 | [16.2](../orb_02-updated_search.html#Q9VHI5) |  | [16.2](../orb_03-phos.html#Q9VHI5) |  | [16.2](http://gemini.scripps.edu/cgi-bin/SeqCov?/scratch/yates/FlyBase_Drosophila_na_02-27-2005_con_reversed.fasta&Q9VHI5&219+36^550+36^591+32&219+36^550+36^591+32*) | (Q9VHI5) CG11984-PA |
| Q9VHK3 | [1.3](../orb_02-updated_search.html#Q9VHK3) |  | [1.3](../orb_03-phos.html#Q9VHK3) |  | [1.3](http://gemini.scripps.edu/cgi-bin/SeqCov?/scratch/yates/FlyBase_Drosophila_na_02-27-2005_con_reversed.fasta&Q9VHK3&1041+17^1879+10&1041+17^1879+10*) | (Q9VHK3) CG31349-PA |
| Q9VHP0 | [3.4](../orb_02-updated_search.html#Q9VHP0) |  | [3.4](../orb_03-phos.html#Q9VHP0) |  | [3.4](http://gemini.scripps.edu/cgi-bin/SeqCov?/scratch/yates/FlyBase_Drosophila_na_02-27-2005_con_reversed.fasta&Q9VHP0&36+17^324+10&36+17^324+10*) | (Q9VHP0) CG9748 protein |
| Q9VI82 | [2.8](../orb_02-updated_search.html#Q9VI82) |  | [2.8](../orb_03-phos.html#Q9VI82) |  | [2.8](http://gemini.scripps.edu/cgi-bin/SeqCov?/scratch/yates/FlyBase_Drosophila_na_02-27-2005_con_reversed.fasta&Q9VI82&726+23&726+23&726+23&726+23*) | (Q9VI82) CG1234 protein (LD35257P) |
| Q9VIG1 | [2.7](../orb_02-updated_search.html#Q9VIG1) |  | [2.7](../orb_03-phos.html#Q9VIG1) |  | [2.7](http://gemini.scripps.edu/cgi-bin/SeqCov?/scratch/yates/FlyBase_Drosophila_na_02-27-2005_con_reversed.fasta&Q9VIG1&760+9^952+18&760+9^952+18*) | (Q9VIG1) CG12050 protein |
| Q9VII9 | [8.9](../orb_02-updated_search.html#Q9VII9) |  | [8.9](../orb_03-phos.html#Q9VII9) |  | [8.9](http://gemini.scripps.edu/cgi-bin/SeqCov?/scratch/yates/FlyBase_Drosophila_na_02-27-2005_con_reversed.fasta&Q9VII9&10+17^97+12&10+17^97+12*) | (Q9VII9) CG31673 protein (LD14730p) |
| Q9VIQ5 | [15.5](../orb_02-updated_search.html#Q9VIQ5) |  | [15.5](../orb_03-phos.html#Q9VIQ5) |  | [15.5](http://gemini.scripps.edu/cgi-bin/SeqCov?/scratch/yates/FlyBase_Drosophila_na_02-27-2005_con_reversed.fasta&Q9VIQ5&2+27^31+12&2+27^31+12*) | (Q9VIQ5) CG10680 protein (RH02620p) |
| Q9VIX5  Q9VIX6 | [16.0](../orb_02-updated_search.html#Q9VIX5) |  | [16.0](../orb_03-phos.html#Q9VIX5) |  | [16.0](http://gemini.scripps.edu/cgi-bin/SeqCov?/scratch/yates/FlyBase_Drosophila_na_02-27-2005_con_reversed.fasta&Q9VIX5&184+42&184+42*)  [14.6](http://gemini.scripps.edu/cgi-bin/SeqCov?/scratch/yates/FlyBase_Drosophila_na_02-27-2005_con_reversed.fasta&Q9VIX6&209+42&209+42*) | (Q9VIX5) CG17549 protein  (Q9VIX6) GH19142p (CG17549-PA) |
| Q9VIX7 | [33.1](../orb_02-updated_search.html#Q9VIX7) |  | [33.1](../orb_03-phos.html#Q9VIX7) |  | [33.1](http://gemini.scripps.edu/cgi-bin/SeqCov?/scratch/yates/FlyBase_Drosophila_na_02-27-2005_con_reversed.fasta&Q9VIX7&27+20^171+34^240+66^347+30^522+17^554+24&27+20^171+34^240+66^347+30^522+17^554+24&34+13^240+10^265+41^362+15^554+16&265+41^362+15^554+16&265+18^555+15*) | (Q9VIX7) CG15825 protein (HL01053p) |
| Q9VJY6 | [18.7](../orb_02-updated_search.html#Q9VJY6) |  | [18.7](../orb_03-phos.html#Q9VJY6) |  | [18.7](http://gemini.scripps.edu/cgi-bin/SeqCov?/scratch/yates/FlyBase_Drosophila_na_02-27-2005_con_reversed.fasta&Q9VJY6&28+7^48+9^81+13&28+7^48+9^81+13*) | (Q9VJY6) CG9282 protein (RE30690p) |
| Q9VK58 | [3.4](../orb_02-updated_search.html#Q9VK58) |  | [3.4](../orb_03-phos.html#Q9VK58) |  | [3.4](http://gemini.scripps.edu/cgi-bin/SeqCov?/scratch/yates/FlyBase_Drosophila_na_02-27-2005_con_reversed.fasta&Q9VK58&660+28^1060+9&660+28^1060+9*) | (Q9VK58) CG5792 protein |
| Q9VK59 | [8.5](../orb_02-updated_search.html#Q9VK59) |  | [6.5](../orb_03-phos.html#Q9VK59) |  | [8.5](http://gemini.scripps.edu/cgi-bin/SeqCov?/scratch/yates/FlyBase_Drosophila_na_02-27-2005_con_reversed.fasta&Q9VK59&356+35^426+27^852+19&356+35^426+27*) | (Q9VK59) CG5787 protein (LD23647p) |
| Q9VKG8 | [6.1](../orb_02-updated_search.html#Q9VKG8) |  | [6.1](../orb_03-phos.html#Q9VKG8) |  | [6.1](http://gemini.scripps.edu/cgi-bin/SeqCov?/scratch/yates/FlyBase_Drosophila_na_02-27-2005_con_reversed.fasta&Q9VKG8&809+31^971+17^1024+42^1375+27&809+31^971+17^1024+42^1375+27*) | (Q9VKG8) CG6509 protein (LD32687p) |
| Q9VL96 | [3.5](../orb_02-updated_search.html#Q9VL96) |  | [3.5](../orb_03-phos.html#Q9VL96) |  | [3.5](http://gemini.scripps.edu/cgi-bin/SeqCov?/scratch/yates/FlyBase_Drosophila_na_02-27-2005_con_reversed.fasta&Q9VL96&390+15^595+7&390+15^595+7*) | (Q9VL96) CG4364 protein (LD27336p) |
| Q9VLK2 | [6.9](../orb_02-updated_search.html#Q9VLK2) |  | [6.9](../orb_03-phos.html#Q9VLK2) |  | [6.9](http://gemini.scripps.edu/cgi-bin/SeqCov?/scratch/yates/FlyBase_Drosophila_na_02-27-2005_con_reversed.fasta&Q9VLK2&80+19^119+15^332+13&80+19^119+15^332+13&80+19^119+15&80+19^119+15*) | (Q9VLK2) CG13096 protein |
| Q9VNG0 | [18.8](../orb_02-updated_search.html#Q9VNG0) |  | [18.8](../orb_03-phos.html#Q9VNG0) |  | [18.8](http://gemini.scripps.edu/cgi-bin/SeqCov?/scratch/yates/FlyBase_Drosophila_na_02-27-2005_con_reversed.fasta&Q9VNG0&106+18^151+14^223+23&106+18^151+14^223+23&106+18&106+18*) | (Q9VNG0) CG1245 protein (RE51713p) |
| Q9VP05 | [17.4](../orb_02-updated_search.html#Q9VP05) |  | [15.6](../orb_03-phos.html#Q9VP05) |  | [17.4](http://gemini.scripps.edu/cgi-bin/SeqCov?/scratch/yates/FlyBase_Drosophila_na_02-27-2005_con_reversed.fasta&Q9VP05&235+16^322+13^393+19^527+59^616+26^739+48^809+18^912+57&235+16^322+13^393+19^527+59^616+26^739+48^809+18^938+31&739+13&739+13*) | (Q9VP05) CG7162 protein (Thyroid hormone receptor-associated protein TRAP220) |
| Q9VP21 | [4.1](../orb_02-updated_search.html#Q9VP21) |  | [4.1](../orb_03-phos.html#Q9VP21) |  | [4.1](http://gemini.scripps.edu/cgi-bin/SeqCov?/scratch/yates/FlyBase_Drosophila_na_02-27-2005_con_reversed.fasta&Q9VP21&15+18^555+8&15+18^555+8*) | (Q9VP21) CG7611 protein (LP01609p) |
| Q9VPR5 | [3.1](../orb_02-updated_search.html#Q9VPR5) |  | [3.1](../orb_03-phos.html#Q9VPR5) |  | [3.1](http://gemini.scripps.edu/cgi-bin/SeqCov?/scratch/yates/FlyBase_Drosophila_na_02-27-2005_con_reversed.fasta&Q9VPR5&279+21^386+21&279+21^386+21*) | (Q9VPR5) CG2807 protein |
| Q9VS38 | [17.4](../orb_02-updated_search.html#Q9VS38) |  | [17.4](../orb_03-phos.html#Q9VS38) |  | [17.4](http://gemini.scripps.edu/cgi-bin/SeqCov?/scratch/yates/FlyBase_Drosophila_na_02-27-2005_con_reversed.fasta&Q9VS38&33+16^131+29&33+16^131+29*) | (Q9VS38) CG8609 protein (LD46084P) |
| Q9VSF2 | [13.6](../orb_02-updated_search.html#Q9VSF2) |  | [13.6](../orb_03-phos.html#Q9VSF2) |  | [13.6](http://gemini.scripps.edu/cgi-bin/SeqCov?/scratch/yates/FlyBase_Drosophila_na_02-27-2005_con_reversed.fasta&Q9VSF2&35+32^242+19^328+35^418+11^690+12^706+11^785+15&35+32^242+19^328+35^418+11^690+12^706+11^785+15*) | (Q9VSF2) CG7999 protein (Thyroid hormone receptor-associated protein TRAP100) |
| Q9VUC1 | [3.6](../orb_02-updated_search.html#Q9VUC1) |  | [3.6](../orb_03-phos.html#Q9VUC1) |  | [3.6](http://gemini.scripps.edu/cgi-bin/SeqCov?/scratch/yates/FlyBase_Drosophila_na_02-27-2005_con_reversed.fasta&Q9VUC1&232+12^327+17&232+12^327+17*) | (Q9VUC1) HSC70CB protein (LD32979P) |
| Q9VUE5 | [3.7](../orb_02-updated_search.html#Q9VUE5) |  | [5.6](../orb_03-phos.html#Q9VUE5) |  | [5.6](http://gemini.scripps.edu/cgi-bin/SeqCov?/scratch/yates/FlyBase_Drosophila_na_02-27-2005_con_reversed.fasta&Q9VUE5&96+20^384+16^486+22&384+16^486+22&384+16*) | (Q9VUE5) STWL protein (LD17962p) |
| Q9VUF1 | [7.3](../orb_02-updated_search.html#Q9VUF1) |  | [7.3](../orb_03-phos.html#Q9VUF1) |  | [7.3](http://gemini.scripps.edu/cgi-bin/SeqCov?/scratch/yates/FlyBase_Drosophila_na_02-27-2005_con_reversed.fasta&Q9VUF1&216+8^335+18&216+8^335+18*) | (Q9VUF1) MPCP protein |
| Q9VUQ5  Q9VUQ6 | [25.1](../orb_02-updated_search.html#Q9VUQ5) |  | [23.7](../orb_03-phos.html#Q9VUQ5) |  | [25.1](http://gemini.scripps.edu/cgi-bin/SeqCov?/scratch/yates/FlyBase_Drosophila_na_02-27-2005_con_reversed.fasta&Q9VUQ5&45+28^153+10^360+71^460+14^535+16^585+15^679+10^694+33^751+18^784+12^835+18^873+10^884+14^1134+28^1202+9&45+28^153+10^360+71^460+14^535+16^585+15^679+10^694+33^751+18^784+12^873+10^884+14^1134+28^1202+9&388+43^585+15^694+14^884+14^1154+8&388+43^585+15^694+14^884+14^1154+8&416+15*)  [25.2](http://gemini.scripps.edu/cgi-bin/SeqCov?/scratch/yates/FlyBase_Drosophila_na_02-27-2005_con_reversed.fasta&Q9VUQ6&42+28^150+10^357+71^457+14^532+16^582+15^676+10^691+33^748+18^781+12^832+18^870+10^881+14^1131+28^1199+9&42+28^150+10^357+71^457+14^532+16^582+15^676+10^691+33^748+18! ^781+12^870+10^881+14^1131+28^1199+9&385+43^582+15^691+14^881+14^1151+8&385+43^582+15^691+14^881+14^1151+8&413+15*) | (Q9VUQ5) CG7439 protein  (Q9VUQ6) CG7439 protein |
| Q9VVI2 | [39.9](../orb_02-updated_search.html#Q9VVI2) |  | [39.9](../orb_03-phos.html#Q9VVI2) |  | [39.9](http://gemini.scripps.edu/cgi-bin/SeqCov?/scratch/yates/FlyBase_Drosophila_na_02-27-2005_con_reversed.fasta&Q9VVI2&30+11^64+59^235+50^357+18^388+16^455+14^503+32^575+60^645+11&30+11^64+59^235+50^357+18^388+16^455+14^503+32^575+60^645+11&30+11^64+39^257+13^388+16^455+14^503+17^582+12^620+15&64+39^257+13^388+16^455+14^503+17^582+12^620+15&73+20^95+8^390+14*) | (Q9VVI2) CG6311 protein |
| Q9VVL6 | [28.8](../orb_02-updated_search.html#Q9VVL6) |  | [18.1](../orb_03-phos.html#Q9VVL6) |  | [28.8](http://gemini.scripps.edu/cgi-bin/SeqCov?/scratch/yates/FlyBase_Drosophila_na_02-27-2005_con_reversed.fasta&Q9VVL6&43+14^90+27^128+25^164+31&43+14^128+25^173+22&43+14^144+9^173+22&43+14^144+9*) | (Q9VVL6) CG5546 protein (LD41395p) |
| Q9VW47 | [7.4](../orb_02-updated_search.html#Q9VW47) |  | [7.4](../orb_03-phos.html#Q9VW47) |  | [7.4](http://gemini.scripps.edu/cgi-bin/SeqCov?/scratch/yates/FlyBase_Drosophila_na_02-27-2005_con_reversed.fasta&Q9VW47&40+25^408+16^480+25^809+17^1219+11^1437+17^1820+11^1899+20^2415+46&40+25^408+16^480+25^809+17^1219+11^1437+17^1820+11^1899+20^2415+46*) | (Q9VW47) CG8491 protein (Transcriptional coactivator kohtalo) |
| Q9VXD5 | [3.6](../orb_02-updated_search.html#Q9VXD5) |  | [2.0](../orb_03-phos.html#Q9VXD5) |  | [3.6](http://gemini.scripps.edu/cgi-bin/SeqCov?/scratch/yates/FlyBase_Drosophila_na_02-27-2005_con_reversed.fasta&Q9VXD5&532+26^566+37^1870+18&532+26^1870+18&1870+18&1870+18*) | (Q9VXD5) CG18572 protein |
| Q9VXV3 | [3.0](../orb_02-updated_search.html#Q9VXV3) |  | [3.0](../orb_03-phos.html#Q9VXV3) |  | [3.0](http://gemini.scripps.edu/cgi-bin/SeqCov?/scratch/yates/FlyBase_Drosophila_na_02-27-2005_con_reversed.fasta&Q9VXV3&33+13^253+17^541+30&33+13^253+17^541+30*) | (Q9VXV3) CG9198 protein |
| Q9VYA9 | [7.2](../orb_02-updated_search.html#Q9VYA9) |  | [7.2](../orb_03-phos.html#Q9VYA9) |  | [7.2](http://gemini.scripps.edu/cgi-bin/SeqCov?/scratch/yates/FlyBase_Drosophila_na_02-27-2005_con_reversed.fasta&Q9VYA9&49+21^104+10&49+21^104+10&104+10&104+10*) | (Q9VYA9) CG12175 protein (SD08423P) |
| Q9VYQ9 | [4.6](../orb_02-updated_search.html#Q9VYQ9) |  | [4.6](../orb_03-phos.html#Q9VYQ9) |  | [4.6](http://gemini.scripps.edu/cgi-bin/SeqCov?/scratch/yates/FlyBase_Drosophila_na_02-27-2005_con_reversed.fasta&Q9VYQ9&110+14^324+8&110+14^324+8*) | (Q9VYQ9) CG1796 protein (LD24662P) |
| Q9VYS4 | [6.6](../orb_02-updated_search.html#Q9VYS4) |  | [6.6](../orb_03-phos.html#Q9VYS4) |  | [6.6](http://gemini.scripps.edu/cgi-bin/SeqCov?/scratch/yates/FlyBase_Drosophila_na_02-27-2005_con_reversed.fasta&Q9VYS4&271+21^391+21^652+19^849+29&271+21^391+21^652+19^849+29&391+21^652+19&391+21^652+19*) | (Q9VYS4) CG15737 protein |
| Q9VZF4 | [5.0](../orb_02-updated_search.html#Q9VZF4) |  | [5.0](../orb_03-phos.html#Q9VZF4) |  | [5.0](http://gemini.scripps.edu/cgi-bin/SeqCov?/scratch/yates/FlyBase_Drosophila_na_02-27-2005_con_reversed.fasta&Q9VZF4&255+20^456+16^800+30&255+20^456+16^800+30&810+20&810+20&817+13*) | (Q9VZF4) LD21322p (LD30271p) (CG15010-PA) (CG15010-PB) |
| Q9VZN0 | [4.5](../orb_02-updated_search.html#Q9VZN0) |  | [4.5](../orb_03-phos.html#Q9VZN0) |  | [4.5](http://gemini.scripps.edu/cgi-bin/SeqCov?/scratch/yates/FlyBase_Drosophila_na_02-27-2005_con_reversed.fasta&Q9VZN0&232+28^586+17&232+28^586+17*) | (Q9VZN0) CG32264 protein |
| Q9W0A8 | [9.0](../orb_02-updated_search.html#Q9W0A8) |  | [9.0](../orb_03-phos.html#Q9W0A8) |  | [9.0](http://gemini.scripps.edu/cgi-bin/SeqCov?/scratch/yates/FlyBase_Drosophila_na_02-27-2005_con_reversed.fasta&Q9W0A8&63+10^210+15&63+10^210+15&210+15&210+15&211+14*) | (Q9W0A8) RPL23A protein |
| Q9W0B8 | [2.8](../orb_02-updated_search.html#Q9W0B8) |  | [2.8](../orb_03-phos.html#Q9W0B8) |  | [2.8](http://gemini.scripps.edu/cgi-bin/SeqCov?/scratch/yates/FlyBase_Drosophila_na_02-27-2005_con_reversed.fasta&Q9W0B8&531+19^952+15&531+19^952+15*) | (Q9W0B8) ALPHACOP protein (LD46584p) |
| Q9W0P3 | [9.4](../orb_02-updated_search.html#Q9W0P3) |  | [9.4](../orb_03-phos.html#Q9W0P3) |  | [9.4](http://gemini.scripps.edu/cgi-bin/SeqCov?/scratch/yates/FlyBase_Drosophila_na_02-27-2005_con_reversed.fasta&Q9W0P3&176+30&176+30*) | (Q9W0P3) CG17183 protein (SD10560P) |
| Q9W0P8 | [20.5](../orb_02-updated_search.html#Q9W0P8) |  | [18.2](../orb_03-phos.html#Q9W0P8) |  | [20.5](http://gemini.scripps.edu/cgi-bin/SeqCov?/scratch/yates/FlyBase_Drosophila_na_02-27-2005_con_reversed.fasta&Q9W0P8&135+20^338+25^382+9^408+23^635+53^727+11^781+26^884+32^1194+25^1226+12^1251+43^1425+40&135+20^338+25^382+9^408+23^656+32^727+11^796+11^884+32^1194+25^1226+12^1251+43^1425+40&338+25^668+20&350+13^668+20&350+13*) | (Q9W0P8) CG12031-PA |
| Q9W0S7 | [4.9](../orb_02-updated_search.html#Q9W0S7) |  | [4.9](../orb_03-phos.html#Q9W0S7) |  | [4.9](http://gemini.scripps.edu/cgi-bin/SeqCov?/scratch/yates/FlyBase_Drosophila_na_02-27-2005_con_reversed.fasta&Q9W0S7&180+19^734+18^906+8&180+19^734+18^906+8*) | (Q9W0S7) CG7008 protein (LD20211p) |
| Q9W105 | [12.7](../orb_02-updated_search.html#Q9W105) |  | [12.7](../orb_03-phos.html#Q9W105) |  | [12.7](http://gemini.scripps.edu/cgi-bin/SeqCov?/scratch/yates/FlyBase_Drosophila_na_02-27-2005_con_reversed.fasta&Q9W105&92+22^131+16^185+11&92+22^131+16^185+11*) | (Q9W105) CG16910 protein |
| Q9W1G7 | [13.0](../orb_02-updated_search.html#Q9W1G7) |  | [13.0](../orb_03-phos.html#Q9W1G7) |  | [13.0](http://gemini.scripps.edu/cgi-bin/SeqCov?/scratch/yates/FlyBase_Drosophila_na_02-27-2005_con_reversed.fasta&Q9W1G7&62+19^145+11^213+18&62+19^145+11^213+18*) | (Q9W1G7) NAP1 protein (LD21576p) |
| Q9W1H5 | [39.5](../orb_02-updated_search.html#Q9W1H5) |  | [46.0](../orb_03-phos.html#Q9W1H5) |  | [46.0](http://gemini.scripps.edu/cgi-bin/SeqCov?/scratch/yates/FlyBase_Drosophila_na_02-27-2005_con_reversed.fasta&Q9W1H5&23+48^102+24^139+23^177+14^207+11^221+14^247+13^263+24&23+48^102+24^139+23^177+14^207+11^221+14^247+13&23+23^58+13^104+12^147+15^207+11^221+14&23+23^58+13^104+12^147+15^207+11^221+14&61+10*) | (Q9W1H5) CG11183 protein |
| Q9W1H8 | [6.8](../orb_02-updated_search.html#Q9W1H8) |  | [6.8](../orb_03-phos.html#Q9W1H8) |  | [6.8](http://gemini.scripps.edu/cgi-bin/SeqCov?/scratch/yates/FlyBase_Drosophila_na_02-27-2005_con_reversed.fasta&Q9W1H8&72+9^330+23&72+9^330+23*) | (Q9W1H8) Thiolase protein (GH13256p) |
| Q9W1X7 | [12.9](../orb_02-updated_search.html#Q9W1X7) |  | [12.3](../orb_03-phos.html#Q9W1X7) |  | [12.9](http://gemini.scripps.edu/cgi-bin/SeqCov?/scratch/yates/FlyBase_Drosophila_na_02-27-2005_con_reversed.fasta&Q9W1X7&40+17^252+38^445+28^541+13^624+18^655+16^1021+24^1295+9^1313+8^1323+14&40+17^252+38^445+28^541+13^624+18^655+16^1021+24^1295+9^1323+14&1295+9^1323+14&1295+9^1323+14*) | (Q9W1X7) Trap150-beta protein |
| Q9W237 | [41.2](../orb_02-updated_search.html#Q9W237) |  | [21.6](../orb_03-phos.html#Q9W237) |  | [41.2](http://gemini.scripps.edu/cgi-bin/SeqCov?/scratch/yates/FlyBase_Drosophila_na_02-27-2005_con_reversed.fasta&Q9W237&7+29^53+10^74+14^93+8&53+10^74+14^93+8*) | (Q9W237) CG4046 protein (RH07540p) |
| Q9W255 | [11.1](../orb_02-updated_search.html#Q9W255) |  | [11.1](../orb_03-phos.html#Q9W255) |  | [11.1](http://gemini.scripps.edu/cgi-bin/SeqCov?/scratch/yates/FlyBase_Drosophila_na_02-27-2005_con_reversed.fasta&Q9W255&20+30^96+14&20+30^96+14&21+29&21+29*) | (Q9W255) QRK58E-1 protein (GH05812p) |
| Q9W278 | [13.3](../orb_02-updated_search.html#Q9W278) |  | [10.8](../orb_03-phos.html#Q9W278) |  | [13.3](http://gemini.scripps.edu/cgi-bin/SeqCov?/scratch/yates/FlyBase_Drosophila_na_02-27-2005_con_reversed.fasta&Q9W278&207+27^338+32^511+11^569+18^685+21&207+27^338+32^511+11^569+18*) | (Q9W278) CG5465 protein |
| Q9W2L8 | [15.2](../orb_02-updated_search.html#Q9W2L8) |  | [15.2](../orb_03-phos.html#Q9W2L8) |  | [15.2](http://gemini.scripps.edu/cgi-bin/SeqCov?/scratch/yates/FlyBase_Drosophila_na_02-27-2005_con_reversed.fasta&Q9W2L8&69+36&69+36*) | (Q9W2L8) CG4038 protein (Anon-Pen101 protein) (Glycine-rich protein 101) (GCR 101 protein) |
| Q9W2U7 | [6.3](../orb_02-updated_search.html#Q9W2U7) |  | [6.3](../orb_03-phos.html#Q9W2U7) |  | [6.3](http://gemini.scripps.edu/cgi-bin/SeqCov?/scratch/yates/FlyBase_Drosophila_na_02-27-2005_con_reversed.fasta&Q9W2U7&189+42^352+33^810+13^1282+15^1452+14^1828+28&189+42^352+33^810+13^1282+15^1452+14^1828+28*) | (Q9W2U7) CG17255 protein |
| Q9W3G1 | [4.8](../orb_02-updated_search.html#Q9W3G1) |  | [4.8](../orb_03-phos.html#Q9W3G1) |  | [4.8](http://gemini.scripps.edu/cgi-bin/SeqCov?/scratch/yates/FlyBase_Drosophila_na_02-27-2005_con_reversed.fasta&Q9W3G1&127+44&127+44*) | (Q9W3G1) CG10555 protein |
| Q9W3W8 | [18.8](../orb_02-updated_search.html#Q9W3W8) |  | [18.8](../orb_03-phos.html#Q9W3W8) |  | [18.8](http://gemini.scripps.edu/cgi-bin/SeqCov?/scratch/yates/FlyBase_Drosophila_na_02-27-2005_con_reversed.fasta&Q9W3W8&87+35&87+35&87+10^106+16&87+10^106+16*) | (Q9W3W8) CG3203 protein (GM02242p) |
| Q9W417  UBIQ_DROME  Q9W418 | [15.3](../orb_02-updated_search.html#Q9W417) |  | [15.3](../orb_03-phos.html#Q9W417) |  | [15.3](http://gemini.scripps.edu/cgi-bin/SeqCov?/scratch/yates/FlyBase_Drosophila_na_02-27-2005_con_reversed.fasta&Q9W417&12+16^195+30&12+16^195+30&201+24&201+24&207+18*)  [60.5](http://gemini.scripps.edu/cgi-bin/SeqCov?/scratch/yates/FlyBase_Drosophila_na_02-27-2005_con_reversed.fasta&UBIQ_DROME&12+16^43+30&12+16^43+30&49+24&49+24&55+18*)  [8.6](http://gemini.scripps.edu/cgi-bin/SeqCov?/scratch/yates/FlyBase_Drosophila_na_02-27-2005_con_reversed.fasta&Q9W418&12+16^43+30&12+16^43+30&49+24&49+24&55+18*) | (Q9W417) CG11700 protein  (Q9VZL4) Ubiquitin  (Q9W418) CG32744 protein |
| Q9W4L2 | [6.9](../orb_02-updated_search.html#Q9W4L2) |  | [6.9](../orb_03-phos.html#Q9W4L2) |  | [6.9](http://gemini.scripps.edu/cgi-bin/SeqCov?/scratch/yates/FlyBase_Drosophila_na_02-27-2005_con_reversed.fasta&Q9W4L2&216+12^464+15^517+18&216+12^464+15^517+18&464+15^517+18&464+15^517+18*) | (Q9W4L2) CG2982 protein (LD40453p) |
| Q9W4M7 | [3.6](../orb_02-updated_search.html#Q9W4M7) |  | [2.5](../orb_03-phos.html#Q9W4M7) |  | [3.6](http://gemini.scripps.edu/cgi-bin/SeqCov?/scratch/yates/FlyBase_Drosophila_na_02-27-2005_con_reversed.fasta&Q9W4M7&526+11^1131+20^1458+21^1545+16&526+11^1458+21^1545+16&1458+21&1458+21*) | (Q9W4M7) CG4857 protein |
| Q9Y0Z1 | [5.7](../orb_02-updated_search.html#Q9Y0Z1) |  | [5.1](../orb_03-phos.html#Q9Y0Z1) |  | [6.8](http://gemini.scripps.edu/cgi-bin/SeqCov?/scratch/yates/FlyBase_Drosophila_na_02-27-2005_con_reversed.fasta&Q9Y0Z1&225+17^944+32^1013+35^1060+28&944+32^1013+35*) | (Q9Y0Z1) BCDNA:LD23876 protein |
| Q9Y108 | [4.0](../orb_02-updated_search.html#Q9Y108) |  | [4.0](../orb_03-phos.html#Q9Y108) |  | [4.0](http://gemini.scripps.edu/cgi-bin/SeqCov?/scratch/yates/FlyBase_Drosophila_na_02-27-2005_con_reversed.fasta&Q9Y108&297+18^347+14&297+18^347+14*) | (Q9Y108) BCDNA:GH11110 protein |
| Q9Y149 | [5.2](../orb_02-updated_search.html#Q9Y149) |  | [5.2](../orb_03-phos.html#Q9Y149) |  | [5.2](http://gemini.scripps.edu/cgi-bin/SeqCov?/scratch/yates/FlyBase_Drosophila_na_02-27-2005_con_reversed.fasta&Q9Y149&586+21^614+18&586+21^614+18*) | (Q9Y149) BCDNA:GH03922 protein |
| R13A_DROME | [28.3](../orb_02-updated_search.html#R13A_DROME) |  | [28.3](../orb_03-phos.html#R13A_DROME) |  | [28.3](http://gemini.scripps.edu/cgi-bin/SeqCov?/scratch/yates/FlyBase_Drosophila_na_02-27-2005_con_reversed.fasta&R13A_DROME&40+12^56+6^106+11^119+9^143+14^200+6&40+12^56+6^106+11^119+9^143+14^200+6&106+11^119+9^143+14&106+11^119+9^143+14*) | (Q9VNE9) 60S ribosomal protein L13A |
| RB27_DROME | [26.8](../orb_02-updated_search.html#RB27_DROME) |  | [26.8](../orb_03-phos.html#RB27_DROME) |  | [26.8](http://gemini.scripps.edu/cgi-bin/SeqCov?/scratch/yates/FlyBase_Drosophila_na_02-27-2005_con_reversed.fasta&RB27_DROME&9+17^49+27^98+16^303+20^332+23&9+17^49+27^98+16^303+20^332+23*) | (P48809) Heterogeneous nuclear ribonucleoprotein 27C (hnRNP 48) (HRP48.1) |
| RF2P_DROME | [37.6](../orb_02-updated_search.html#RF2P_DROME) |  | [37.6](../orb_03-phos.html#RF2P_DROME) |  | [37.6](http://gemini.scripps.edu/cgi-bin/SeqCov?/scratch/yates/FlyBase_Drosophila_na_02-27-2005_con_reversed.fasta&RF2P_DROME&36+10^79+19^101+39^160+29^196+19^226+51^347+24^462+34&36+10^79+19^101+39^160+29^196+19^226+51^347+24^462+34&168+21^196+19^265+12^486+10&168+21^196+19^265+12^486+10*) | (P14199) Ref(2)P protein (Refractory to sigma P) |
| RL10_DROME | [34.9](../orb_02-updated_search.html#RL10_DROME) |  | [34.9](../orb_03-phos.html#RL10_DROME) |  | [34.9](http://gemini.scripps.edu/cgi-bin/SeqCov?/scratch/yates/FlyBase_Drosophila_na_02-27-2005_con_reversed.fasta&RL10_DROME&41+29^131+9^144+11^165+14^199+13&41+29^131+9^144+11^165+14^199+13&199+13&199+13*) | (O61231) 60S ribosomal protein L10 (QM protein homolog) (dQM) |
| RL14_DROME | [20.5](../orb_02-updated_search.html#RL14_DROME) |  | [20.5](../orb_03-phos.html#RL14_DROME) |  | [20.5](http://gemini.scripps.edu/cgi-bin/SeqCov?/scratch/yates/FlyBase_Drosophila_na_02-27-2005_con_reversed.fasta&RL14_DROME&24+24^122+10&24+24^122+10*) | (P55841) 60S ribosomal protein L14 |
| RL19_DROME | [11.8](../orb_02-updated_search.html#RL19_DROME) |  | [11.8](../orb_03-phos.html#RL19_DROME) |  | [11.8](http://gemini.scripps.edu/cgi-bin/SeqCov?/scratch/yates/FlyBase_Drosophila_na_02-27-2005_con_reversed.fasta&RL19_DROME&22+17^191+7&22+17^191+7*) | (P36241) 60S ribosomal protein L19 |
| RL1X_DROME | [30.5](../orb_02-updated_search.html#RL1X_DROME) |  | [30.5](../orb_03-phos.html#RL1X_DROME) |  | [30.5](http://gemini.scripps.edu/cgi-bin/SeqCov?/scratch/yates/FlyBase_Drosophila_na_02-27-2005_con_reversed.fasta&RL1X_DROME&32+18^77+7^99+13^121+16&32+18^77+7^99+13^121+16&44+6&44+6*) | (P41093) 60S ribosomal protein L18a |
| RL2A_DROME | [23.5](../orb_02-updated_search.html#RL2A_DROME) |  | [23.5](../orb_03-phos.html#RL2A_DROME) |  | [23.5](http://gemini.scripps.edu/cgi-bin/SeqCov?/scratch/yates/FlyBase_Drosophila_na_02-27-2005_con_reversed.fasta&RL2A_DROME&14+9^70+18^99+8&14+9^70+18^99+8*) | (P41092) 60S ribosomal protein L27a |
| RL7_DROME | [23.8](../orb_02-updated_search.html#RL7_DROME) |  | [23.8](../orb_03-phos.html#RL7_DROME) |  | [23.8](http://gemini.scripps.edu/cgi-bin/SeqCov?/scratch/yates/FlyBase_Drosophila_na_02-27-2005_con_reversed.fasta&RL7_DROME&138+14^168+13^184+33&138+14^168+13^184+33&170+11^207+10&170+11*) | (P32100) 60S ribosomal protein L7 |
| RNT1_DROME | [49.7](../orb_02-updated_search.html#RNT1_DROME) |  | [50.5](../orb_03-phos.html#RNT1_DROME) |  | 50.5 | (Q9VYS3) Regulator of nonsense transcripts 1 homolog |
| RS12_DROME | [51.1](../orb_02-updated_search.html#RS12_DROME) |  | [51.1](../orb_03-phos.html#RS12_DROME) |  | [51.1](http://gemini.scripps.edu/cgi-bin/SeqCov?/scratch/yates/FlyBase_Drosophila_na_02-27-2005_con_reversed.fasta&RS12_DROME&32+16^53+17^71+15^91+10^120+13&32+16^53+17^71+15^91+10^120+13&32+16^71+15&32+16^71+15&34+14*) | (P80455) 40S ribosomal protein S12 |
| RS13_DROME | [18.0](../orb_02-updated_search.html#RS13_DROME) |  | [18.0](../orb_03-phos.html#RS13_DROME) |  | [18.0](http://gemini.scripps.edu/cgi-bin/SeqCov?/scratch/yates/FlyBase_Drosophila_na_02-27-2005_con_reversed.fasta&RS13_DROME&9+10^76+17&9+10^76+17&76+17&76+17*) | (Q03334) 40S ribosomal protein S13 |
| RS14_DROME | [38.4](../orb_02-updated_search.html#RS14_DROME) |  | [39.7](../orb_03-phos.html#RS14_DROME) |  | [39.7](http://gemini.scripps.edu/cgi-bin/SeqCov?/scratch/yates/FlyBase_Drosophila_na_02-27-2005_con_reversed.fasta&RS14_DROME&11+12^64+35^129+13&11+12^64+21^87+12^129+13&67+18^87+10&67+18^87+10&72+13^87+10*) | (P14130) 40S ribosomal protein S14 |
| RS18_DROME | [21.1](../orb_02-updated_search.html#RS18_DROME) |  | [21.1](../orb_03-phos.html#RS18_DROME) |  | [21.1](http://gemini.scripps.edu/cgi-bin/SeqCov?/scratch/yates/FlyBase_Drosophila_na_02-27-2005_con_reversed.fasta&RS18_DROME&67+20^95+12&67+20^95+12*) | (P41094) 40S ribosomal protein S18 |
| RS1A_DROME | [56.6](../orb_02-updated_search.html#RS1A_DROME) |  | [56.6](../orb_03-phos.html#RS1A_DROME) |  | [56.6](http://gemini.scripps.edu/cgi-bin/SeqCov?/scratch/yates/FlyBase_Drosophila_na_02-27-2005_con_reversed.fasta&RS1A_DROME&3+9^32+25^78+39&3+9^32+25^78+39&3+9^78+10&3+9^78+10*) | (P48149) 40S ribosomal protein S15A |
| RS26_DROME | [23.7](../orb_02-updated_search.html#RS26_DROME) |  | [23.7](../orb_03-phos.html#RS26_DROME) |  | [23.7](http://gemini.scripps.edu/cgi-bin/SeqCov?/scratch/yates/FlyBase_Drosophila_na_02-27-2005_con_reversed.fasta&RS26_DROME&52+15^71+12&52+15^71+12*) | (P13008) 40S ribosomal protein S26 (DS31) |
| RS3A_DROME | [31.5](../orb_02-updated_search.html#RS3A_DROME) |  | [28.5](../orb_03-phos.html#RS3A_DROME) |  | [31.5](http://gemini.scripps.edu/cgi-bin/SeqCov?/scratch/yates/FlyBase_Drosophila_na_02-27-2005_con_reversed.fasta&RS3A_DROME&28+6^65+11^97+34^170+15^190+8^206+10&28+6^65+11^97+34^170+15^206+10&65+11^97+34^206+10&65+11^97+15^118+13^206+10&118+13^206+10*) | (P55830) 40S ribosomal protein S3a (C3 protein) |
| RS3_DROME | [32.9](../orb_02-updated_search.html#RS3_DROME) |  | [32.9](../orb_03-phos.html#RS3_DROME) |  | [32.9](http://gemini.scripps.edu/cgi-bin/SeqCov?/scratch/yates/FlyBase_Drosophila_na_02-27-2005_con_reversed.fasta&RS3_DROME&13+17^43+14^154+22^204+13^230+15&13+17^43+14^154+22^204+13^230+15*) | (Q06559) 40S ribosomal protein S3 |
| Reverse_Q86BQ1 | [1.4](../orb_02-updated_search.html#Reverse_Q86BQ1) |  | [1.4](../orb_03-phos.html#Reverse_Q86BQ1) |  | [1.4](http://gemini.scripps.edu/cgi-bin/SeqCov?/scratch/yates/FlyBase_Drosophila_na_02-27-2005_con_reversed.fasta&Reverse_Q86BQ1&286+9^372+8&286+9^372+8*) | (Q86BQ1) CG32316-PD (CG32316-PF) |
| Reverse_Q9I7U4 | [0.2](../orb_02-updated_search.html#Reverse_Q9I7U4) |  | [0.1](../orb_03-phos.html#Reverse_Q9I7U4) |  | [0.3](http://gemini.scripps.edu/cgi-bin/SeqCov?/scratch/yates/FlyBase_Drosophila_na_02-27-2005_con_reversed.fasta&Reverse_Q9I7U4&554+12^2030+11^3191+16^12096+11&12096+11*) | (Q9I7U4) CG1915 protein |
| PhReverse_Q9VFC4 | [0.8](../orb_02-updated_search.html#Reverse_Q9VFC4) |  | [0.8](../orb_03-phos.html#Reverse_Q9VFC4) |  | [0.8](http://gemini.scripps.edu/cgi-bin/SeqCov?/scratch/yates/FlyBase_Drosophila_na_02-27-2005_con_reversed.fasta&Reverse_Q9VFC4&249+10&249+10&251+8&251+8*) | (Q9VFC4) CG6752 protein |
| SSRP_DROME | [4.4](../orb_02-updated_search.html#SSRP_DROME) |  | [6.9](../orb_03-phos.html#SSRP_DROME) |  | [6.9](http://gemini.scripps.edu/cgi-bin/SeqCov?/scratch/yates/FlyBase_Drosophila_na_02-27-2005_con_reversed.fasta&SSRP_DROME&218+11^242+7^326+21^453+11&218+11^326+21&326+21&326+21*) | (Q05344) Single-strand recognition protein (SSRP) (Chorion-factor 5) |
| TBB3_DROME | [26.9](../orb_02-updated_search.html#TBB3_DROME) |  | [26.9](../orb_03-phos.html#TBB3_DROME) |  | [26.9](http://gemini.scripps.edu/cgi-bin/SeqCov?/scratch/yates/FlyBase_Drosophila_na_02-27-2005_con_reversed.fasta&TBB3_DROME&84+26^140+21^169+12^248+10^259+24^289+15^343+14&84+26^140+21^169+12^248+10^259+24^289+15^343+14&269+14&269+14*) | (P08841) Tubulin beta-3 chain |
| U202_DROME | [4.1](../orb_02-updated_search.html#U202_DROME) |  | [4.1](../orb_03-phos.html#U202_DROME) |  | [4.1](http://gemini.scripps.edu/cgi-bin/SeqCov?/scratch/yates/FlyBase_Drosophila_na_02-27-2005_con_reversed.fasta&U202_DROME&51+10^82+13^252+18&51+10^82+13^252+18*) | (Q9W3C1) Polycomb protein CG1994 (p110 protein) |
| VATB_DROME | [4.1](../orb_02-updated_search.html#VATB_DROME) |  | [4.1](../orb_03-phos.html#VATB_DROME) |  | [4.1](http://gemini.scripps.edu/cgi-bin/SeqCov?/scratch/yates/FlyBase_Drosophila_na_02-27-2005_con_reversed.fasta&VATB_DROME&168+20&168+20&168+20&168+20*) | (P31409) Vacuolar ATP synthase subunit B (EC 3.6.3.14) (V-ATPase B subunit) (Vacuolar proton pump B subunit) (V-ATPase 57 kDa subunit) |
| 171 | X |  | X |  |  |  |

Orb IP and Dorsal IP

| Locus | Orb IP | Dorsal IP | Orb IP | Dorsal IP | Total | Description |
| --- | --- | --- | --- | --- | --- | --- |
| AAF46547  VIT2_DROME | [52.3](../orb_02-updated_search.html#AAF46547) | [33.6](../dorsal_02-updated_search.html#AAF46547) | [51.6](../orb_03-phos.html#AAF46547) | [33.6](../dorsal_03-phos.html#AAF46547) | [56.4](http://gemini.scripps.edu/cgi-bin/SeqCov?/scratch/yates/FlyBase_Drosophila_na_02-27-2005_con_reversed.fasta&AAF46547&48+26^79+39^128+11^145+22^179+19^219+26^253+32^319+48^382+14^404+22&48+26^79+39^128+11^145+22^179+19^219+26^253+32^319+48^382+14&48+26^79+39^128+11^145+22^219+26^261+24^319+48^382+14&48+26^79+39^128+11^145+22^219+26^319+48^382+14&79+39^128+11^145+22^219+26^319+48^384+12*)  [58.6](http://gemini.scripps.edu/cgi-bin/SeqCov?/scratch/yates/FlyBase_Drosophila_na_02-27-2005_con_reversed.fasta&VIT2_DRO! ME&31+26^62+39^111+11^128+22^162+19^202+26^236+32^302+48^365+14^387+22&31+26^62+39^111+11^128+22^162+19^202+26^236+32^302+48^365+14&31+26^62+39^111+11^128+22^202+26^244+24^302+48^365+14&31+26^62+39^111+11^128+22^202+26^302+48^365+14&62+39^111+11^128+22^202+26^302+48^367+12*) | (AAF46547) CG2979-PA  (P02844) Vitellogenin II precursor (Yolk protein 2) |
| AAF49905 | [47.5](../orb_02-updated_search.html#AAF49905) | [36.5](../dorsal_02-updated_search.html#AAF49905) | [45.2](../orb_03-phos.html#AAF49905) | [36.5](../dorsal_03-phos.html#AAF49905) | 55.5 ! | (AAF49905) CG10686-PA |
| AAF51478 | [3.1](../orb_02-updated_search.html#AAF51478) | [8.4](../dorsal_02-updated_search.html#AAF51478) | [3.1](../orb_03-phos.html#AAF51478) | [8.4](../dorsal_03-phos.html#AAF51478) | [11.5](http://gemini.scripps.edu/cgi-bin/SeqCov?/scratch/yates/FlyBase_Drosophila_na_02-27-2005_con_reversed.fasta&AAF51478&7+15^28+28^270+21^314+28^377+21^465+22^1304+19&7+15^28+28^270+21^314+28^377+21^465+22^1304+19&28+28^322+20&28+28^322+20*) | (AAF51478) CG2807-PA |
| AAF57941  EF1B_DROME | [13.0](../orb_02-updated_search.html#AAF57941) | [14.2](../dorsal_02-updated_search.html#AAF57941) | [13.0](../orb_03-phos.html#AAF57941) | [14.2](../dorsal_03-phos.html#AAF57941) | [27.2](http://gemini.scripps.edu/cgi-bin/SeqCov?/scratch/yates/FlyBase_Drosophila_na_02-27-2005_con_reversed.fasta&AAF57941&53+30^107+23^243+18&53+30^107+23^243+18*)  [32.1](http://gemini.scripps.edu/cgi-bin/SeqCov?/scratch/yates/FlyBase_Drosophila_na_02-27-2005_con_reversed.fasta&EF1B_DROME&13+30^67+23^203+18&13+30^67+23^203+18*) | (AAF57941) CG6341-PA  (O96827) Probable elongation factor 1-beta (EF-1-beta) |
| AAM68717 | [21.5](../orb_02-updated_search.html#AAM68717) | [14.8](../dorsal_02-updated_search.html#AAM68717) | [21.5](../orb_03-phos.html#AAM68717) | [14.8](../dorsal_03-phos.html#AAM68717) | [30.3](http://gemini.scripps.edu/cgi-bin/SeqCov?/scratch/yates/FlyBase_Drosophila_na_02-27-2005_con_reversed.fasta&AAM68717&75+42^169+38^302+20&75+42^169+38^302+20&302+20&302+20*) | (AAM68717) CG8996-PA (CG8996-PB) |
| AAN09050  RSP4_DROME | [36.4](../orb_02-updated_search.html#AAN09050) | [16.6](../dorsal_02-updated_search.html#AAN09050) | [36.4](../orb_03-phos.html#AAN09050) | [16.6](../dorsal_03-phos.html#AAN09050) | [36.4](http://gemini.scripps.edu/cgi-bin/SeqCov?/scratch/yates/FlyBase_Drosophila_na_02-27-2005_con_reversed.fasta&AAN09050&107+17^146+15^164+46^269+36&107+17^146+15^164+46^269+36&107+17^164+35&107+17^164+35*)  [42.2](http://gemini.scripps.edu/cgi-bin/SeqCov?/scratch/yates/FlyBase_Drosophila_na_02-27-2005_con_reversed.fasta&RSP4_DROME&64+17^103+15^121+46^226+36&64+17^103+15^121+46^226+36&64+17^121+35&64+17^121+35*) | (AAN09050) CG14792-PD  (P38979) 40S ribosomal protein SA (p40) (Stubarista protein! ) (Laminin receptor homolog) (K14) |
| AAN14138  Q8IMM4 | [10.3](../orb_02-updated_search.html#AAN14138) | [8.5](../dorsal_02-updated_search.html#AAN14138) | [7.9](../orb_03-phos.html#AAN14138) | [3.9](../dorsal_03-phos.html#AAN14138) | [17.2](http://gemini.scripps.edu/cgi-bin/SeqCov?/scratch/yates/FlyBase_Drosophila_na_02-27-2005_con_reversed.fasta&AAN14138&134+22^238+54^382+27^503+16^775+16^943+37^985+13^1029+40^1240+16&134+22^238+23^267+10^503+16^775+16^1029+40^1240+16&238+23^267+10^1029+40&238+23^1029+40*)  [17.2](http://gemini.scripps.edu/cgi-bin/SeqCov?/scratch/yates/FlyBase_Drosophila_na_02-27-2005_con_reversed.fasta&Q8IMM4&134+22^238+54^382+27^503+16^775+16^943+37^985+13^1029+40^1240+16&134+22^238+23^267+10^503+16^775+16^1029+40^1240+16&238+23^267! +10^1029+40&238+23^1029+40*) | (AAN14138) CG14066-PC (Fragment)  (Q8IMM4) CG14066-PC |
| AAS64975  PEP_DROME | [10.8](../orb_02-updated_search.html#AAS64975) | [5.5](../dorsal_02-updated_search.html#AAS64975) | [10.8](../orb_03-phos.html#AAS64975) | [5.5](../dorsal_03-phos.html#AAS64975) | [12.7](http://gemini.scripps.edu/cgi-bin/SeqCov?/scratch/yates/FlyBase_Drosophila_na_02-27-2005_con_reversed.fasta&AAS64975&53+23^201+13^336+14^474+13^642+25&53+23^201+13^336+14^474+13^642+25&201+13^642+25&201+13^642+25*)  [12.3](http://gemini.scripps.edu/cgi-bin/SeqCov?/scratch/yates/FlyBase_Drosophila_na_02-27-2005_con_reversed.fasta&PEP_DROME&76+23^224+13^359+14^497+13^665+25&76+23^224+13^359+14^497+13^665+25&224+13^665+25&224+13^665+25*) | (AAS64975) CG6143-PC  (P41073) Zinc finger protein on ecdy! sone puffs |
| ACT1_DROME | [41.5](../orb_02-updated_search.html#ACT1_DROME) | [22.6](../dorsal_02-updated_search.html#ACT1_DROME) | [41.5](../orb_03-phos.html#ACT1_DROME) | [19.7](../dorsal_03-phos.html#ACT1_DROME) | [47.1](http://gemini.scripps.edu/cgi-bin/SeqCov?/scratch/yates/FlyBase_Drosophila_na_02-27-2005_con_reversed.fasta&ACT1_DROME&30+11^86+29^149+30^185+8^217+39^258+28^293+21^317+11&30+11^86+29^149+30^185+8^217+39^258+28^293+21^317+11&30+11^86+29^149+30^185+8^217+23^244+12^317+11&30+9^86+29^149+30^185+8^217+23^317+11&30+9^86+11^149+30^217+23*) | (P10987) Actin-5C |
| ACT3_DROME  ACT5_DROME | [36.7](../orb_02-updated_search.html#ACT3_DROME) | [22.6](../dorsal_02-updated_search.html#ACT3_DROME) | [36.7](../orb_03-phos.html#ACT3_DROME) | [19.7](../dorsal_03-phos.html#ACT3_DROME) | [48.4](http://gemini.scripps.edu/cgi-bin/SeqCov?/scratch/yates/FlyBase_Drosophila_na_02-27-2005_con_reversed.fasta&ACT3_DROME&30+11^70+45^149+30^185+8^217+39^258+28^293+21&30+11^70+45^149+30^185+8^217+39^258+28^293+21&30+11^86+29^149+30^185+8^217+23^244+12&30+9^86+29^149+30^185+8^217+23&30+9^86+11^149+30*)  [48.4](http://gemini.scripps.edu/cgi-bin/SeqCov?/scratch/yates/FlyBase_Drosophila_na_02-27-2005_con_reversed.fasta&ACT5_DROME&30+11^70+45^149+30^185+8^217+39^258+28^293+21&30+11^70+45^149+30^185+8^217+3! 9^258+28^293+21&30+11^86+29^149+30^185+8^217+23^244+12&30+9^86+29^149+30^185+8^217+23&30+9^86+11^149+30*) | (P53501) Actin 57B  (P10981) Actin-87E |
| ADT_DROME  Q8IRA0 | [56.5](../orb_02-updated_search.html#ADT_DROME) | [31.8](../dorsal_02-updated_search.html#ADT_DROME) | [56.5](../orb_03-phos.html#ADT_DROME) | [33.4](../dorsal_03-phos.html#ADT_DROME) | [62.5](http://gemini.scripps.edu/cgi-bin/SeqCov?/scratch/yates/FlyBase_Drosophila_na_02-27-2005_con_reversed.fasta&ADT_DROME&4+27^36+10^55+8^83+12^99+42^154+11^168+33^246+28^282+16&4+22^36+10^55+8^83+12^99+42^154+11^168+33^246+28^282+16&4+22^36+10^85+10^114+27^154+11^171+30^254+11^282+16&4+22^36+10^85+10^114+27^154+11^171+30^254+11^282+16&4+22^85+10^114+27^254+11^282+15*)  [59.9](http://gemini.scripps.edu/cgi-bin/SeqCov?/scratch/yates/FlyBase_Drosophila_na_02-27-2005_con_reversed.fasta&Q8IRA0&17+27^49+10^68+8^96+12! ^112+42^167+11^181+33^259+28^295+16&17+22^49+10^68+8^96+12^112+42^167+11^181+33^259+28^295+16&17+22^49+10^98+10^127+27^167+11^184+30^267+11^295+16&17+22^49+10^98+10^127+27^167+11^184+30^267+11^295+16&17+22^98+10^127+27^267+11^295+15*) | (Q26365) ADP,ATP carrier protein (ADP/ATP translocase) (Adenine nucleotide translocator) (ANT) (Stress sensitive B protein)  (Q8IRA0) CG16944-PC |
| ATPA_DROME | [17.2](../orb_02-updated_search.html#ATPA_DROME) | [13.4](../dorsal_02-updated_search.html#ATPA_DROME) | [17.2](../orb_03-phos.html#ATPA_DROME) | [13.4](../dorsal_03-phos.html#ATPA_DROME) | [23.0](http://gemini.scripps.edu/cgi-bin/SeqCov?/scratch/yates/FlyBase_Drosophila_na_02-27-2005_con_reversed.fasta&ATPA_DROME&47+11^83+40^133+16^218+12^402+14^441+22^516+12&47+11^83+40^133+16^218+12^402+14^441+22^516+12&103+20^441+22&103+20^441+22*) | (P35381) ATP synthase alpha chain, mitochondrial precursor (EC 3.6.3.14) (Protein bellwether) |
| ATPB_DROME | [15.2](../orb_02-updated_search.html#ATPB_DROME) | [23.0](../dorsal_02-updated_search.html#ATPB_DROME) | [19.4](../orb_03-phos.html#ATPB_DROME) | [23.0](../dorsal_03-phos.html#ATPB_DROME) | [37.4](http://gemini.scripps.edu/cgi-bin/SeqCov?/scratch/yates/FlyBase_Drosophila_na_02-27-2005_con_reversed.fasta&ATPB_DROME&72+15^121+12^190+13^219+18^259+13^288+25^328+56^410+19^440+18&72+15^121+12^190+13^219+18^259+13^288+14^328+56^410+19^440+18&72+15^292+10^329+36&72+15^329+36*) | (Q05825) ATP synthase beta chain, mitochondrial precursor (EC 3.6.3.14) |
| CH60_DROME | [5.4](../orb_02-updated_search.html#CH60_DROME) | [13.1](../dorsal_02-updated_search.html#CH60_DROME) | [5.4](../orb_03-phos.html#CH60_DROME) | [13.1](../dorsal_03-phos.html#CH60_DROME) | [18.5](http://gemini.scripps.edu/cgi-bin/SeqCov?/scratch/yates/FlyBase_Drosophila_na_02-27-2005_con_reversed.fasta&CH60_DROME&93+25^202+13^233+13^247+18^307+23^335+14&93+25^202+13^233+13^247+18^307+23^335+14&93+25&93+25*) | (O02649) 60 kDa heat shock protein, mitochondrial precursor (Hsp60) (60 kDa chaperonin) (CPN60) (Heat shock protein 60) (HSP-60) (Mitochondrial matrix protein P1) |
| CUP_DROME | [32.9](../orb_02-updated_search.html#CUP_DROME) | [9.7](../dorsal_02-updated_search.html#CUP_DROME) | [28.5](../orb_03-phos.html#CUP_DROME) | [9.7](../dorsal_03-phos.html#CUP_DROME) | [36.3](http://gemini.scripps.edu/cgi-bin/SeqCov?/scratch/yates/FlyBase_Drosophila_na_02-27-2005_con_reversed.fasta&CUP_DROME&65+22^181+24^352+10^401+22^425+32^496+32^552+13^578+31^730+21^766+17^805+36^884+11^901+80^1057+22^1095+38&65+22^181+24^352+10^401+22^425+32^496+32^552+13^578+31^730+21^766+17^805+36^884+11^921+31^1057+22^1095+38&65+22^496+19^553+12^730+21^814+27^884+11^921+31^1057+22^1119+14&65+22^496+19^553+12^730+21^814+27^884+11^921+31^1057+22^1119+14&67+20^931+21*) | (Q9VMA3) Cup protein (Oskar ribonucleoprotein complex 147 kDa ! subunit) |
| EF2_DROME | [15.3](../orb_02-updated_search.html#EF2_DROME) | [12.9](../dorsal_02-updated_search.html#EF2_DROME) | [13.0](../orb_03-phos.html#EF2_DROME) | [7.0](../dorsal_03-phos.html#EF2_DROME) | [25.1](http://gemini.scripps.edu/cgi-bin/SeqCov?/scratch/yates/FlyBase_Drosophila_na_02-27-2005_con_reversed.fasta&EF2_DROME&154+9^166+41^327+25^442+25^545+39^591+20^634+19^753+34&154+9^166+18^327+25^442+25^545+13^591+20^753+34&339+13^442+25^557+1&339+13^442+25*) | (P13060) Elongation factor 2 (EF-2) |
| FKB4_DROME | [24.4](../orb_02-updated_search.html#FKB4_DROME) | [20.4](../dorsal_02-updated_search.html#FKB4_DROME) | [22.4](../orb_03-phos.html#FKB4_DROME) | [20.4](../dorsal_03-phos.html#FKB4_DROME) | [34.5](http://gemini.scripps.edu/cgi-bin/SeqCov?/scratch/yates/FlyBase_Drosophila_na_02-27-2005_con_reversed.fasta&FKB4_DROME&22+11^55+13^75+36^271+9^286+7^298+20^323+13^341+14&22+11^55+13^75+36^271+9^298+20^323+13^341+14&22+11^55+13^298+20^341+14&22+11^55+13^298+20^341+14&298+10*) | (P54397) 39 kDa FK506-binding nuclear protein (EC 5.2.1.8) (Peptidyl-prolyl cis-trans isomerase) (PPIase) (Rotamase) |
| HS7C_DROME | [32.6](../orb_02-updated_search.html#HS7C_DROME) | [10.1](../dorsal_02-updated_search.html#HS7C_DROME) | [32.6](../orb_03-phos.html#HS7C_DROME) | [10.1](../dorsal_03-phos.html#HS7C_DROME) | [37.8](http://gemini.scripps.edu/cgi-bin/SeqCov?/scratch/yates/FlyBase_Drosophila_na_02-27-2005_con_reversed.fasta&HS7C_DROME&27+21^62+14^103+12^124+29^164+18^307+30^355+13^448+27^482+11^563+11^592+27^622+35&27+21^62+14^103+12^124+29^164+18^307+30^355+13^448+27^482+11^563+11^592+27^622+35&62+14^127+12^164+18^307+18^327+10^563+11^592+12^622+12&62+14^127+12^164+18^307+18^327+10^563+11^592+12^622+12&165+17^563+8^594+10*) | (P29844) Heat shock 70 kDa protein cognate 3 precursor (78 kDa glucose regulated protein homolog) (GRP 78) (Heat ! shock protein cognate 72) |
| HS7D_DROME | [47.0](../orb_02-updated_search.html#HS7D_DROME) | [31.6](../dorsal_02-updated_search.html#HS7D_DROME) | [47.0](../orb_03-phos.html#HS7D_DROME) | [31.6](../dorsal_03-phos.html#HS7D_DROME) | 53.5 | (P11147) Heat shock 70 kDa protein cognate 4 (Heat shock 70 kDa protein 88E) |
| HS83_DROME | [12.3](../orb_02-updated_search.html#HS83_DROME) | [7.9](../dorsal_02-updated_search.html#HS83_DROME) | [13.5](../orb_03-phos.html#HS83_DROME) | [7.9](../dorsal_03-phos.html#HS83_DROME) | [21.5](http://gemini.scripps.edu/cgi-bin/SeqCov?/scratch/yates/FlyBase_Drosophila_na_02-27-2005_con_reversed.fasta&HS83_DROME&73+28^142+20^198+10^285+28^353+19^450+19^487+9^577+21&73+28^142+20^198+10^285+28^353+19^450+19^577+21*) | (P02828) Heat shock protein 83 (HSP 82) |
| IF6_DROME | [13.1](../orb_02-updated_search.html#IF6_DROME) | [15.5](../dorsal_02-updated_search.html#IF6_DROME) | [13.1](../orb_03-phos.html#IF6_DROME) | [15.5](../dorsal_03-phos.html#IF6_DROME) | [18.8](http://gemini.scripps.edu/cgi-bin/SeqCov?/scratch/yates/FlyBase_Drosophila_na_02-27-2005_con_reversed.fasta&IF6_DROME&5+14^165+24^238+8&5+14^165+24^238+8&165+24&165+24&165+24*) | (P56538) Probable eukaryotic translation initiation factor 6 (eIF-6) |
| KPYK_DROME | [16.5](../orb_02-updated_search.html#KPYK_DROME) | [12.9](../dorsal_02-updated_search.html#KPYK_DROME) | [16.5](../orb_03-phos.html#KPYK_DROME) | [12.9](../dorsal_03-phos.html#KPYK_DROME) | [24.8](http://gemini.scripps.edu/cgi-bin/SeqCov?/scratch/yates/FlyBase_Drosophila_na_02-27-2005_con_reversed.fasta&KPYK_DROME&36+11^124+15^177+13^194+34^323+17^346+25^387+17&36+11^124+15^177+13^194+34^323+17^346+25^387+17&346+25&346+25*) | (O62619) Pyruvate kinase (EC 2.7.1.40) (PK) |
| ME31_DROME | [42.0](../orb_02-updated_search.html#ME31_DROME) | [44.0](../dorsal_02-updated_search.html#ME31_DROME) | [42.0](../orb_03-phos.html#ME31_DROME) | [44.0](../dorsal_03-phos.html#ME31_DROME) | [61.0](http://gemini.scripps.edu/cgi-bin/SeqCov?/scratch/yates/FlyBase_Drosophila_na_02-27-2005_con_reversed.fasta&ME31_DROME&17+19^57+40^109+43^212+16^231+16^255+53^315+14^348+27^389+16^424+36&17+19^57+40^109+43^212+16^231+16^255+53^315+14^348+27^389+16^424+36&17+19^57+10^77+20^125+12^212+16^231+16^283+10^389+16^424+36&17+19^57+10^77+20^125+12^212+16^231+16^283+10^389+16^424+36&17+19^81+16^212+16^238+9^283+10^389+16*) | (P23128) Putative ATP-dependent RNA helicase me31b (Maternal expression at 31B) |
| MYSN_DROME | [6.1](../orb_02-updated_search.html#MYSN_DROME) | [3.7](../dorsal_02-updated_search.html#MYSN_DROME) | [5.3](../orb_03-phos.html#MYSN_DROME) | [3.7](../dorsal_03-phos.html#MYSN_DROME) | [8.8](http://gemini.scripps.edu/cgi-bin/SeqCov?/scratch/yates/FlyBase_Drosophila_na_02-27-2005_con_reversed.fasta&MYSN_DROME&252+33^300+21^433+14^1159+17^1353+21^1469+16^1590+17^1728+18^1782+21&252+33^300+21^433+14^1353+21^1469+16^1590+17^1728+18^1782+21&1782+21&1782+21*) | (Q99323) Myosin heavy chain, non-muscle (Zipper protein) (Myosin II) |
| NADE_DROME | [5.3](../orb_02-updated_search.html#NADE_DROME) | [10.5](../dorsal_02-updated_search.html#NADE_DROME) | [5.3](../orb_03-phos.html#NADE_DROME) | [10.5](../dorsal_03-phos.html#NADE_DROME) | [15.9](http://gemini.scripps.edu/cgi-bin/SeqCov?/scratch/yates/FlyBase_Drosophila_na_02-27-2005_con_reversed.fasta&NADE_DROME&5+21^82+12^167+16^258+22^347+24^393+19^615+11&5+21^82+12^167+16^258+22^347+24^393+19^615+11*) | (Q9VYA0) Putative glutamine-dependent NAD(+) synthetase (EC 6.3.5.1) (NAD(+) synthase [glutamine-hydrolyzing]) |
| NO60_DROME | [20.7](../orb_02-updated_search.html#NO60_DROME) | [5.5](../dorsal_02-updated_search.html#NO60_DROME) | [20.7](../orb_03-phos.html#NO60_DROME) | [5.5](../dorsal_03-phos.html#NO60_DROME) | [23.0](http://gemini.scripps.edu/cgi-bin/SeqCov?/scratch/yates/FlyBase_Drosophila_na_02-27-2005_con_reversed.fasta&NO60_DROME&42+14^87+22^176+14^210+16^283+13^409+12^442+26&42+14^87+22^176+14^210+16^283+13^409+12^442+26&95+14^176+14^210+16^283+13&95+14^176+14^210+16^283+13&95+14^179+11*) | (O44081) Nucleolar protein AT band 60B (Minifly protein) |
| NOG1_DROME | [2.1](../orb_02-updated_search.html#NOG1_DROME) | [6.3](../dorsal_02-updated_search.html#NOG1_DROME) | [2.1](../orb_03-phos.html#NOG1_DROME) | [6.3](../dorsal_03-phos.html#NOG1_DROME) | [8.4](http://gemini.scripps.edu/cgi-bin/SeqCov?/scratch/yates/FlyBase_Drosophila_na_02-27-2005_con_reversed.fasta&NOG1_DROME&277+14^315+19^527+22&277+14^315+19^527+22&277+14&277+14*) | (Q9V411) Probable nucleolar GTP-binding protein 1 |
| O61380 | [9.7](../orb_02-updated_search.html#O61380) | [5.8](../dorsal_02-updated_search.html#O61380) | [10.3](../orb_03-phos.html#O61380) | [4.9](../dorsal_03-phos.html#O61380) | [16.5](http://gemini.scripps.edu/cgi-bin/SeqCov?/scratch/yates/FlyBase_Drosophila_na_02-27-2005_con_reversed.fasta&O61380&113+17^296+41^339+34^565+21^599+20^665+12^876+12^1020+26^1059+12^1259+17^1291+17^1343+19^1526+21^1559+6&113+17^296+41^349+24^565+21^599+20^665+12^876+12^1020+26^1059+12^1259+17^1291+17^1343+19&349+24*) | (O61380) EIF-4G protein |
| Q7PL86  RL15_DROME | [25.0](../orb_02-updated_search.html#Q7PL86) | [14.2](../dorsal_02-updated_search.html#Q7PL86) | [25.0](../orb_03-phos.html#Q7PL86) | [14.2](../dorsal_03-phos.html#Q7PL86) | [25.0](http://gemini.scripps.edu/cgi-bin/SeqCov?/scratch/yates/FlyBase_Drosophila_na_02-27-2005_con_reversed.fasta&Q7PL86&25+7^57+7^78+8^115+29&25+7^57+7^78+8^115+29&115+29&115+29&129+15*)  [25.0](http://gemini.scripps.edu/cgi-bin/SeqCov?/scratch/yates/FlyBase_Drosophila_na_02-27-2005_con_reversed.fasta&RL15_DROME&25+7^57+7^78+8^115+29&25+7^57+7^78+8^115+29&115+29&115+29&129+15*) | (Q7PL86) CG17420-PA  (O17445) 60S ribosomal protein L15 |
| Q8IMK2  Q9VAF1  Q8MLY8 | [21.2](../orb_02-updated_search.html#Q8IMK2) | [24.0](../dorsal_02-updated_search.html#Q8IMK2) | [21.2](../orb_03-phos.html#Q8IMK2) | [24.0](../dorsal_03-phos.html#Q8IMK2) | [38.0](http://gemini.scripps.edu/cgi-bin/SeqCov?/scratch/yates/FlyBase_Drosophila_na_02-27-2005_con_reversed.fasta&Q8IMK2&60+15^78+15^99+25^160+13&60+15^78+15^99+25^160+13&111+13^160+13&111+13^160+13*)  [32.5](http://gemini.scripps.edu/cgi-bin/SeqCov?/scratch/yates/FlyBase_Drosophila_na_02-27-2005_con_reversed.fasta&Q9VAF1&61+15^79+15^100+25^161+13&61+15^79+15^100+25^161+13&112+13^161+13&112+13^161+13*)  [32.7](http://gemini.scripps.edu/cgi-bin/SeqCov?/scratch/yates/FlyBase_Drosophila_na_02-27! -2005_con_reversed.fasta&Q8MLY8&60+15^78+15^99+25^160+13&60+15^78+15^99+25^160+13&111+13^160+13&111+13^160+13*) | (Q8IMK2) CG7808-PD  (Q9VAF1) CG7808-PB (Fragment)  (Q8MLY8) RH06886p (SD17528p) (CG7808-PC) |
| Q8MKQ2  Q9V358 | [41.3](../orb_02-updated_search.html#Q8MKQ2) | [5.7](../dorsal_02-updated_search.html#Q8MKQ2) | [42.6](../orb_03-phos.html#Q8MKQ2) | [3.6](../dorsal_03-phos.html#Q8MKQ2) | 43.3  43.0 | (Q8MKQ2) CG8715-PB  (Q9V358) CG8715 protein |
| Q8T3U2 | [21.7](../orb_02-updated_search.html#Q8T3U2) | [28.7](../dorsal_02-updated_search.html#Q8T3U2) | [28.7](../orb_03-phos.html#Q8T3U2) | [28.7](../dorsal_03-phos.html#Q8T3U2) | [28.7](http://gemini.scripps.edu/cgi-bin/SeqCov?/scratch/yates/FlyBase_Drosophila_na_02-27-2005_con_reversed.fasta&Q8T3U2&78+30^125+11&78+30^125+11&78+30^125+11&88+20^125+11&88+20*) | (Q8T3U2) GM14585p (CG8415 protein) |
| Q9I7D3 | [61.6](../orb_02-updated_search.html#Q9I7D3) | [3.6](../dorsal_02-updated_search.html#Q9I7D3) | [61.2](../orb_03-phos.html#Q9I7D3) | [3.6](../dorsal_03-phos.html#Q9I7D3) | 61.6 | (Q9I7D3) CG18811 protein |
| Q9V3B2 | [29.5](../orb_02-updated_search.html#Q9V3B2) | [17.2](../dorsal_02-updated_search.html#Q9V3B2) | [26.1](../orb_03-phos.html#Q9V3B2) | [17.2](../dorsal_03-phos.html#Q9V3B2) | [40.6](http://gemini.scripps.edu/cgi-bin/SeqCov?/scratch/yates/FlyBase_Drosophila_na_02-27-2005_con_reversed.fasta&Q9V3B2&33+13^77+17^207+31^253+10^291+47^345+31^381+37^440+19&33+13^207+31^253+10^291+47^345+31^381+37^440+19&291+39^345+31&291+39^345+31*) | (Q9V3B2) GBP protein |
| Q9V3C3 | [28.0](../orb_02-updated_search.html#Q9V3C3) | [14.9](../dorsal_02-updated_search.html#Q9V3C3) | [28.0](../orb_03-phos.html#Q9V3C3) | [14.1](../dorsal_03-phos.html#Q9V3C3) | [38.0](http://gemini.scripps.edu/cgi-bin/SeqCov?/scratch/yates/FlyBase_Drosophila_na_02-27-2005_con_reversed.fasta&Q9V3C3&17+26^113+40^171+37^254+21^335+21^363+12^463+21^485+15^527+14^565+17^597+15^613+36^675+16^752+13&23+20^113+40^171+37^254+21^335+21^363+12^463+21^485+15^527+14^565+17^597+15^613+36^675+16^752+13&23+13^113+23^335+21^675+16&113+23^335+21^675+16&113+21*) | (Q9V3C3) TER94 protein |
| Q9V5R0 | [7.8](../orb_02-updated_search.html#Q9V5R0) | [8.3](../dorsal_02-updated_search.html#Q9V5R0) | [7.8](../orb_03-phos.html#Q9V5R0) | [8.3](../dorsal_03-phos.html#Q9V5R0) | [10.7](http://gemini.scripps.edu/cgi-bin/SeqCov?/scratch/yates/FlyBase_Drosophila_na_02-27-2005_con_reversed.fasta&Q9V5R0&309+13^329+36&309+13^329+36&329+25&329+25*) | (Q9V5R0) CG6751 protein |
| Q9V6C4 | [10.8](../orb_02-updated_search.html#Q9V6C4) | [11.1](../dorsal_02-updated_search.html#Q9V6C4) | [8.9](../orb_03-phos.html#Q9V6C4) | [11.1](../dorsal_03-phos.html#Q9V6C4) | [19.6](http://gemini.scripps.edu/cgi-bin/SeqCov?/scratch/yates/FlyBase_Drosophila_na_02-27-2005_con_reversed.fasta&Q9V6C4&4+24^251+14^269+15^324+36^483+15^545+12^590+15^610+9^655+28^749+7&251+14^269+15^324+36^483+15^545+12^590+15^610+9^655+28&545+12^590+15&545+12^590+15*) | (Q9V6C4) CG8545 protein |
| Q9VAX8 | [3.1](../orb_02-updated_search.html#Q9VAX8) | [5.3](../dorsal_02-updated_search.html#Q9VAX8) | [3.1](../orb_03-phos.html#Q9VAX8) | [5.3](../dorsal_03-phos.html#Q9VAX8) | [8.4](http://gemini.scripps.edu/cgi-bin/SeqCov?/scratch/yates/FlyBase_Drosophila_na_02-27-2005_con_reversed.fasta&Q9VAX8&499+13^658+19^717+17^876+16^918+17&499+13^658+19^717+17^876+16^918+17*) | (Q9VAX8) CG4849 protein (LD28793p) |
| Q9VEN7 | [6.2](../orb_02-updated_search.html#Q9VEN7) | [9.1](../dorsal_02-updated_search.html#Q9VEN7) | [6.2](../orb_03-phos.html#Q9VEN7) | [9.1](../dorsal_03-phos.html#Q9VEN7) | [12.7](http://gemini.scripps.edu/cgi-bin/SeqCov?/scratch/yates/FlyBase_Drosophila_na_02-27-2005_con_reversed.fasta&Q9VEN7&22+10^147+15^346+11^361+21^514+17&22+10^147+15^346+11^361+21^514+17&516+15&516+15*) | (Q9VEN7) CG3983-PB (CG3983-PA) |
| Q9VH07 | [16.2](../orb_02-updated_search.html#Q9VH07) | [14.9](../dorsal_02-updated_search.html#Q9VH07) | [10.7](../orb_03-phos.html#Q9VH07) | [14.9](../dorsal_03-phos.html#Q9VH07) | [28.1](http://gemini.scripps.edu/cgi-bin/SeqCov?/scratch/yates/FlyBase_Drosophila_na_02-27-2005_con_reversed.fasta&Q9VH07&77+14^118+35^172+30^206+20^379+15^405+14&77+14^129+24^172+30^206+20^379+15^405+14&77+14^129+24*) | (Q9VH07) Putative pontin protein (LD08555p) |
| Q9VJ19 | [45.0](../orb_02-updated_search.html#Q9VJ19) | [27.9](../dorsal_02-updated_search.html#Q9VJ19) | [45.0](../orb_03-phos.html#Q9VJ19) | [27.9](../dorsal_03-phos.html#Q9VJ19) | [45.0](http://gemini.scripps.edu/cgi-bin/SeqCov?/scratch/yates/FlyBase_Drosophila_na_02-27-2005_con_reversed.fasta&Q9VJ19&43+15^69+19^91+16&43+15^69+19^91+16&45+12^69+19&45+12^69+19&45+12*) | (Q9VJ19) CG10652 protein (RE25263p) |
| Q9VN21 | [12.3](../orb_02-updated_search.html#Q9VN21) | [14.7](../dorsal_02-updated_search.html#Q9VN21) | [12.3](../orb_03-phos.html#Q9VN21) | [10.8](../dorsal_03-phos.html#Q9VN21) | [22.0](http://gemini.scripps.edu/cgi-bin/SeqCov?/scratch/yates/FlyBase_Drosophila_na_02-27-2005_con_reversed.fasta&Q9VN21&178+21^222+16^422+8^434+38^500+37&222+16^422+8^434+38^500+37&422+8^445+27&422+8^445+27&445+27*) | (Q9VN21) CG14648 protein (LD30155P) |
| Q9VS34 | [30.3](../orb_02-updated_search.html#Q9VS34) | [25.0](../dorsal_02-updated_search.html#Q9VS34) | [30.3](../orb_03-phos.html#Q9VS34) | [25.0](../dorsal_03-phos.html#Q9VS34) | [30.3](http://gemini.scripps.edu/cgi-bin/SeqCov?/scratch/yates/FlyBase_Drosophila_na_02-27-2005_con_reversed.fasta&Q9VS34&56+10^72+20^98+13^116+14&56+10^72+20^98+13^116+14&72+20^98+13^116+14&72+20^98+13^116+14&72+20^122+8*) | (Q9VS34) CG8615 protein (Ribosomal protein L18) (RH01814p) |
| Q9VV75 | [13.9](../orb_02-updated_search.html#Q9VV75) | [15.7](../dorsal_02-updated_search.html#Q9VV75) | [9.3](../orb_03-phos.html#Q9VV75) | [15.7](../dorsal_03-phos.html#Q9VV75) | [20.2](http://gemini.scripps.edu/cgi-bin/SeqCov?/scratch/yates/FlyBase_Drosophila_na_02-27-2005_con_reversed.fasta&Q9VV75&130+20^209+28^256+16^391+25&209+28^256+16^391+25&256+16^391+25&256+16^391+25&261+11*) | (Q9VV75) CG4169 protein (AT02348p) |
| Q9W1B9 | [44.8](../orb_02-updated_search.html#Q9W1B9) | [39.4](../dorsal_02-updated_search.html#Q9W1B9) | [44.8](../orb_03-phos.html#Q9W1B9) | [39.4](../dorsal_03-phos.html#Q9W1B9) | [44.8](http://gemini.scripps.edu/cgi-bin/SeqCov?/scratch/yates/FlyBase_Drosophila_na_02-27-2005_con_reversed.fasta&Q9W1B9&17+24^100+15^131+35&17+24^100+15^131+35&17+24^100+15^131+35&17+24^100+15^131+35&34+7^100+15*) | (Q9W1B9) RE28824p (CG3195-PA) (CG3195-PB) |
| Q9W1V3 | [18.6](../orb_02-updated_search.html#Q9W1V3) | [11.3](../dorsal_02-updated_search.html#Q9W1V3) | [24.1](../orb_03-phos.html#Q9W1V3) | [11.3](../dorsal_03-phos.html#Q9W1V3) | [29.9](http://gemini.scripps.edu/cgi-bin/SeqCov?/scratch/yates/FlyBase_Drosophila_na_02-27-2005_con_reversed.fasta&Q9W1V3&22+19^169+19^250+31^291+19^330+15&169+19^250+31^291+19^330+15&270+11^291+19^330+14&270+11^291+19^330+14*) | (Q9W1V3) FIB protein |
| Q9W229 | [37.4](../orb_02-updated_search.html#Q9W229) | [25.2](../dorsal_02-updated_search.html#Q9W229) | [20.6](../orb_03-phos.html#Q9W229) | [25.2](../dorsal_03-phos.html#Q9W229) | [37.4](http://gemini.scripps.edu/cgi-bin/SeqCov?/scratch/yates/FlyBase_Drosophila_na_02-27-2005_con_reversed.fasta&Q9W229&23+16^45+18^70+15&45+18^70+15&45+18^70+15&51+12^70+15&51+12*) | (Q9W229) CG3751 protein (RE59324p) |
| Q9W5R8 | [51.5](../orb_02-updated_search.html#Q9W5R8) | [32.8](../dorsal_02-updated_search.html#Q9W5R8) | [51.5](../orb_03-phos.html#Q9W5R8) | [32.8](../dorsal_03-phos.html#Q9W5R8) | [51.5](http://gemini.scripps.edu/cgi-bin/SeqCov?/scratch/yates/FlyBase_Drosophila_na_02-27-2005_con_reversed.fasta&Q9W5R8&55+14^74+34^113+40^159+20^197+25^233+10^282+11&55+14^74+34^113+40^159+20^197+25^233+10^282+11&55+14^74+34^113+40^159+20^197+24^233+10^282+11&55+14^74+34^113+40^159+20^197+24^233+10^282+11&59+10^86+22^133+20^165+14^197+24^233+9*) | (Q9W5R8) RE33114p (CG17489-PA.3) |
| RFA1_DROME | [14.3](../orb_02-updated_search.html#RFA1_DROME) | [7.3](../dorsal_02-updated_search.html#RFA1_DROME) | [14.3](../orb_03-phos.html#RFA1_DROME) | [7.3](../dorsal_03-phos.html#RFA1_DROME) | [21.6](http://gemini.scripps.edu/cgi-bin/SeqCov?/scratch/yates/FlyBase_Drosophila_na_02-27-2005_con_reversed.fasta&RFA1_DROME&2+12^96+17^115+12^145+34^227+15^416+14^435+14^572+12&2+12^96+17^115+12^145+34^227+15^416+14^435+14^572+12&146+33&146+33*) | (Q24492) Replication protein A 70 kDa DNA-binding subunit (RP-A) (RF-A) (Replication factor-A protein 1) (Single-stranded DNA-binding protein) (DmRPA1) |
| RL11_DROME | [12.5](../orb_02-updated_search.html#RL11_DROME) | [16.8](../dorsal_02-updated_search.html#RL11_DROME) | [12.5](../orb_03-phos.html#RL11_DROME) | [16.8](../dorsal_03-phos.html#RL11_DROME) | [29.3](http://gemini.scripps.edu/cgi-bin/SeqCov?/scratch/yates/FlyBase_Drosophila_na_02-27-2005_con_reversed.fasta&RL11_DROME&25+13^44+14^124+18^175+9&25+13^44+14^124+18^175+9&175+9&175+9*) | (P46222) 60S ribosomal protein L11 |
| RL22_DROME | [29.8](../orb_02-updated_search.html#RL22_DROME) | [14.7](../dorsal_02-updated_search.html#RL22_DROME) | [29.8](../orb_03-phos.html#RL22_DROME) | [14.7](../dorsal_03-phos.html#RL22_DROME) | [29.8](http://gemini.scripps.edu/cgi-bin/SeqCov?/scratch/yates/FlyBase_Drosophila_na_02-27-2005_con_reversed.fasta&RL22_DROME&24+9^65+23^138+24^193+21^225+12&24+9^65+23^138+24^193+21^225+12&65+23^138+24^193+21&65+23^138+24^193+21&138+24*) | (P50887) 60S ribosomal protein L22 |
| RL3_DROME | [28.2](../orb_02-updated_search.html#RL3_DROME) | [18.8](../dorsal_02-updated_search.html#RL3_DROME) | [28.0](../orb_03-phos.html#RL3_DROME) | [13.5](../dorsal_03-phos.html#RL3_DROME) | [28.2](http://gemini.scripps.edu/cgi-bin/SeqCov?/scratch/yates/FlyBase_Drosophila_na_02-27-2005_con_reversed.fasta&RL3_DROME&39+11^100+16^203+21^250+11^312+29^385+9^396+20&39+11^100+15^203+21^250+11^312+29^385+9^396+20&39+11^100+15^203+21^250+11^312+22^385+8^396+20&39+11^100+15^203+21^250+11^312+22^385+8^396+20&39+11^100+15^312+22^396+20*) | (O16797) 60S ribosomal protein L3 |
| RL4_DROME | [23.6](../orb_02-updated_search.html#RL4_DROME) | [10.1](../dorsal_02-updated_search.html#RL4_DROME) | [23.6](../orb_03-phos.html#RL4_DROME) | [10.1](../dorsal_03-phos.html#RL4_DROME) | [29.0](http://gemini.scripps.edu/cgi-bin/SeqCov?/scratch/yates/FlyBase_Drosophila_na_02-27-2005_con_reversed.fasta&RL4_DROME&24+25^53+22^125+39^226+12^252+20&24+25^53+22^125+39^226+12^252+20&24+25^125+39^263+9&33+16^125+39^263+9*) | (P09180) 60S ribosomal protein L4 (L1) |
| RL9_DROME | [75.8](../orb_02-updated_search.html#RL9_DROME) | [44.2](../dorsal_02-updated_search.html#RL9_DROME) | [75.8](../orb_03-phos.html#RL9_DROME) | [44.2](../dorsal_03-phos.html#RL9_DROME) | [90.0](http://gemini.scripps.edu/cgi-bin/SeqCov?/scratch/yates/FlyBase_Drosophila_na_02-27-2005_con_reversed.fasta&RL9_DROME&3+19^24+66^92+75^172+11&3+19^24+66^92+75^172+11&12+6^37+53^92+22^123+44^173+10&12+6^37+53^92+22^123+44^173+10&40+12^58+5^70+20^92+22^124+16^173+10*) | (P50882) 60S ribosomal protein L9 |
| RLA0_DROME | [70.0](../orb_02-updated_search.html#RLA0_DROME) | [59.6](../dorsal_02-updated_search.html#RLA0_DROME) | [71.9](../orb_03-phos.html#RLA0_DROME) | [56.8](../dorsal_03-phos.html#RLA0_DROME) | [79.5](http://gemini.scripps.edu/cgi-bin/SeqCov?/scratch/yates/FlyBase_Drosophila_na_02-27-2005_con_reversed.fasta&RLA0_DROME&11+28^45+48^109+104^215+46^275+26&11+28^45+33^84+9^109+104^215+46^275+26&17+22^49+9^63+15^84+9^113+100^215+46^275+19&17+22^49+9^63+15^84+2^113+100^215+46^275+19&17+22^49+9^63+15^113+54^184+29^215+25^247+14^275+19*) | (P19889) 60S acidic ribosomal protein P0 (DNA-(apurinic or apyrimidinic site) lyase) (EC 4.2.99.18) (Apurinic-apyrimidinic endonuclease) |
| RLA1_DROME | [70.5](../orb_02-updated_search.html#RLA1_DROME) | [70.5](../dorsal_02-updated_search.html#RLA1_DROME) | [70.5](../orb_03-phos.html#RLA1_DROME) | [70.5](../dorsal_03-phos.html#RLA1_DROME) | [70.5](http://gemini.scripps.edu/cgi-bin/SeqCov?/scratch/yates/FlyBase_Drosophila_na_02-27-2005_con_reversed.fasta&RLA1_DROME&5+23^34+56&5+23^34+56&5+23^34+56&5+23^34+56&5+23^39+11^58+32*) | (P08570) 60S acidic ribosomal protein P1 (RP21C) (Acidic ribosomal protein RPA2) |
| RLA2_DROME | [44.2](../orb_02-updated_search.html#RLA2_DROME) | [28.3](../dorsal_02-updated_search.html#RLA2_DROME) | [44.2](../orb_03-phos.html#RLA2_DROME) | [28.3](../dorsal_03-phos.html#RLA2_DROME) | [44.2](http://gemini.scripps.edu/cgi-bin/SeqCov?/scratch/yates/FlyBase_Drosophila_na_02-27-2005_con_reversed.fasta&RLA2_DROME&3+13^20+19^42+18&3+13^20+19^42+18&3+13^20+19&3+13^20+19&3+13^26+13*) | (P05389) 60S acidic ribosomal protein P2 (Acidic ribosomal protein RPA1) |
| RS17_DROME | [62.3](../orb_02-updated_search.html#RS17_DROME) | [16.9](../dorsal_02-updated_search.html#RS17_DROME) | [62.3](../orb_03-phos.html#RS17_DROME) | [16.9](../dorsal_03-phos.html#RS17_DROME) | [62.3](http://gemini.scripps.edu/cgi-bin/SeqCov?/scratch/yates/FlyBase_Drosophila_na_02-27-2005_con_reversed.fasta&RS17_DROME&23+37^81+22^107+22&23+37^81+22^107+22&33+14^49+11^81+22&33+14^49+11^81+22&44+3^49+11^81+22*) | (P17704) 40S ribosomal protein S17 |
| RS2_DROME | [30.0](../orb_02-updated_search.html#RS2_DROME) | [12.7](../dorsal_02-updated_search.html#RS2_DROME) | [30.0](../orb_03-phos.html#RS2_DROME) | [12.7](../dorsal_03-phos.html#RS2_DROME) | [30.0](http://gemini.scripps.edu/cgi-bin/SeqCov?/scratch/yates/FlyBase_Drosophila_na_02-27-2005_con_reversed.fasta&RS2_DROME&60+32^196+15^230+33&60+32^196+15^230+33&73+19^196+15^230+11&73+19^196+15^230+11&73+19*) | (P31009) 40S ribosomal protein S2 (Strings of pearls protein) |
| RS4_DROME | [36.4](../orb_02-updated_search.html#RS4_DROME) | [17.6](../dorsal_02-updated_search.html#RS4_DROME) | [36.4](../orb_03-phos.html#RS4_DROME) | [17.6](../dorsal_03-phos.html#RS4_DROME) | [36.4](http://gemini.scripps.edu/cgi-bin/SeqCov?/scratch/yates/FlyBase_Drosophila_na_02-27-2005_con_reversed.fasta&RS4_DROME&76+19^135+11^149+20^175+13^199+13^222+19&76+19^135+11^149+20^175+13^199+13^222+19&78+17^135+11^149+20^199+13^222+19&78+17^135+11^149+20^199+13^222+19&78+17^135+11^199+13^222+9*) | (P41042) 40S ribosomal protein S4 |
| RS6_DROME | [25.0](../orb_02-updated_search.html#RS6_DROME) | [12.9](../dorsal_02-updated_search.html#RS6_DROME) | [19.4](../orb_03-phos.html#RS6_DROME) | [12.9](../dorsal_03-phos.html#RS6_DROME) | [31.9](http://gemini.scripps.edu/cgi-bin/SeqCov?/scratch/yates/FlyBase_Drosophila_na_02-27-2005_con_reversed.fasta&RS6_DROME&3+21^31+21^99+17^120+12^205+8&3+21^32+15^99+17^120+12&3+21^32+15&3+21^32+15&32+15*) | (P29327) 40S ribosomal protein S6 |
| TBA1_DROME  TBA3_DROME | [35.6](../orb_02-updated_search.html#TBA1_DROME) | [24.7](../dorsal_02-updated_search.html#TBA1_DROME) | [31.1](../orb_03-phos.html#TBA1_DROME) | [24.7](../dorsal_03-phos.html#TBA1_DROME) | [44.7](http://gemini.scripps.edu/cgi-bin/SeqCov?/scratch/yates/FlyBase_Drosophila_na_02-27-2005_con_reversed.fasta&TBA1_DROME&41+20^65+15^85+12^113+9^230+75^327+26^374+17^395+7^403+20&41+20^65+15^85+12^113+9^230+75^327+26^374+17^395+7^403+20&41+20^65+15^85+12^230+51^333+4^340+13&65+15^230+51^333+4^340+13*)  [44.7](http://gemini.scripps.edu/cgi-bin/SeqCov?/scratch/yates/FlyBase_Drosophila_na_02-27-2005_con_reversed.fasta&TBA3_DROME&41+20^65+15^85+12^113+9^230+75^327+26^374+17^395+7^403+20&41+20^65+15^85+12^! 113+9^230+75^327+26^374+17^395+7^403+20&41+20^65+15^85+12^230+51^333+4^340+13&65+15^230+51^333+4^340+13*) | (P06603) Tubulin alpha-1 chain  (P06605) Tubulin alpha-3 chain |
| TBA4_DROME | [16.7](../orb_02-updated_search.html#TBA4_DROME) | [12.1](../dorsal_02-updated_search.html#TBA4_DROME) | [16.7](../orb_03-phos.html#TBA4_DROME) | [12.1](../dorsal_03-phos.html#TBA4_DROME) | [28.8](http://gemini.scripps.edu/cgi-bin/SeqCov?/scratch/yates/FlyBase_Drosophila_na_02-27-2005_con_reversed.fasta&TBA4_DROME&40+22^76+15^178+10^241+51^350+14^442+21&40+22^76+15^178+10^241+51^350+14^442+21&351+13&351+13&351+13*) | (P06606) Tubulin alpha-4 chain |
| TBB1_DROME | [52.6](../orb_02-updated_search.html#TBB1_DROME) | [23.5](../dorsal_02-updated_search.html#TBB1_DROME) | [52.6](../orb_03-phos.html#TBB1_DROME) | [23.5](../dorsal_03-phos.html#TBB1_DROME) | [55.9](http://gemini.scripps.edu/cgi-bin/SeqCov?/scratch/yates/FlyBase_Drosophila_na_02-27-2005_con_reversed.fasta&TBB1_DROME&20+39^63+92^163+12^217+35^253+24^283+15^310+9^337+14^381+10&20+39^63+92^163+12^217+35^253+24^283+15^310+9^337+14^381+10&20+39^78+44^123+32^263+14^310+9^381+10&20+39^78+44^123+32^263+14^310+9^381+10&47+12^84+38^123+32*) | (Q24560) Tubulin beta-1 chain |
| TBG2_DROME | [16.2](../orb_02-updated_search.html#TBG2_DROME) | [15.8](../dorsal_02-updated_search.html#TBG2_DROME) | [16.2](../orb_03-phos.html#TBG2_DROME) | [15.8](../dorsal_03-phos.html#TBG2_DROME) | [28.2](http://gemini.scripps.edu/cgi-bin/SeqCov?/scratch/yates/FlyBase_Drosophila_na_02-27-2005_con_reversed.fasta&TBG2_DROME&49+15^85+29^170+24^198+15^374+17^427+29&49+15^85+29^170+24^198+15^374+17^427+29&374+17&374+17*) | (P42271) Tubulin gamma-2 chain (Gamma-2 tubulin) |
| TCPA_DROME | [2.0](../orb_02-updated_search.html#TCPA_DROME) | [9.7](../dorsal_02-updated_search.html#TCPA_DROME) | [2.0](../orb_03-phos.html#TCPA_DROME) | [9.7](../dorsal_03-phos.html#TCPA_DROME) | [11.7](http://gemini.scripps.edu/cgi-bin/SeqCov?/scratch/yates/FlyBase_Drosophila_na_02-27-2005_con_reversed.fasta&TCPA_DROME&47+20^115+11^163+21^545+13&47+20^115+11^163+21^545+13&115+11&115+11*) | (P12613) T-complex protein 1, alpha subunit (TCP-1-alpha) (CCT-alpha) |
| VIT1_DROME | [58.1](../orb_02-updated_search.html#VIT1_DROME) | [24.4](../dorsal_02-updated_search.html#VIT1_DROME) | [58.1](../orb_03-phos.html#VIT1_DROME) | [24.4](../dorsal_03-phos.html#VIT1_DROME) | [58.1](http://gemini.scripps.edu/cgi-bin/SeqCov?/scratch/yates/FlyBase_Drosophila_na_02-27-2005_con_reversed.fasta&VIT1_DROME&25+33^66+49^119+23^199+14^219+8^234+37^301+48^364+21^386+22&25+33^66+49^119+23^199+14^219+8^234+37^301+48^364+21^386+22&35+23^66+38^119+23^199+14^219+8^245+26^301+48^386+22&35+23^66+38^119+23^199+14^219+8^245+26^301+48^386+22&66+38^119+23^201+12^260+11*) | (P02843) Vitellogenin I precursor (Yolk protein 1) |
| VIT3_DROME | [48.8](../orb_02-updated_search.html#VIT3_DROME) | [16.9](../dorsal_02-updated_search.html#VIT3_DROME) | [46.2](../orb_03-phos.html#VIT3_DROME) | [16.9](../dorsal_03-phos.html#VIT3_DROME) | [51.7](http://gemini.scripps.edu/cgi-bin/SeqCov?/scratch/yates/FlyBase_Drosophila_na_02-27-2005_con_reversed.fasta&VIT3_DROME&33+20^76+20^114+19^144+16^190+76^281+31^327+12^354+11^376+12&33+20^76+20^114+19^144+16^190+76^281+31^327+12^376+12&33+20^76+20^114+19^152+8^190+68^291+21&33+20^76+20^114+19^152+8^190+68^291+21&84+12^210+40*) | (P06607) Vitellogenin III precursor (Yolk protein 3) |
| 68 | X | X | X | X |  |  |

**Legend**: Ovary extracts were immunoprecipiated with Orb and Dorsal antibodies as previously described [26]. The immunopreciiptated samples were then analyzed as described in [24] and [24].
